# Proceedings of the 23^rd^ Annual Meeting of the Portuguese Society of Human Genetics

**DOI:** 10.1097/MD.0000000000019291

**Published:** 2020-02-28

**Coimbra, 14–16 November 2019**

## 1 Oral communications

**Basic Research**

**OC1-The prognostic significance of E-cadherin in Gastric Cancer: an integrative approach based on patients’ cohort and CRISPR-Cas9 engineered cell models**

Carla Pereira^1,2^, Marta Ferreira^1,2^, Patrícia Oliveira^1,2^, Fátima Carneiro^2,3^, Gabriela M. Almeida^1,2,3^, Carla Oliveira^1,2,3^

1- Institute of Molecular Pathology and Immunology of the University of Porto (IPATIMUP), 4200-135 Porto, Portugal; 2- Instituto de Investigação e Inovação em Saúde (i3S), University of Porto, 4200-135 Porto, Portugal; 3- Dept. Pathology and Oncology, Faculty of Medicine, University of Porto, 4200 - 319 Porto, Portugal

E-cadherin/*CDH1* dysfunction is a well-established event in GC initiation and progression in nearly 80% of gastric cancers (GC), independently of histological type. While E-cadherin permanent loss is the trigger for diffuse GC (DGC), transient aberrant expression is common along progression in intestinal GC (IGC). DGC has poorer prognosis than IGC and it spreads to the peritoneum, while IGC metastasizes to distant organs. We hypothesize that the timing (initiation vs progression) and mode of E-cadherin loss of function (permanent vs persistent; complete loss vs aberrant) determine the GC pattern of tumour spreading and prognosis and therefore explored the underlying mechanisms. The pattern of E-cadherin expression was analyzed by immunohistochemistry and correlated with clinicopathological features and overall survival (OS) in 284 patients. Permanent (CRISPR-Cas9) and transient (RNAi) E-cadherin depleted cell models representative of DGC and IGC were established and characterized by RNA-sequencing and label-free quantitative proteomics profiling followed by bioinformatics analysis. GC presenting aberrant E-cadherin expression were more often IGC, more advanced, more often spread to distant organs, and displayed poorer prognosis than GC with complete E-cadherin loss or normal E-cadherin expression. Remarkably, GCs with absent/residual E-cadherin expression were more often DGC. Proteomics and transcriptomic profiling revealed that transient and permanent E-cadherin depletion in the DGC model dramatically impairs cell-cell (adherens, tight juntions and desmosomes), and cell-matrix adhesion. The same manipulations in the IGC model led to cadherin-switch and downregulation of adherens junction and cell motility proteins. Our study demonstrates that E-cadherin dysfunction is associated with poor prognosis. Our data supports the hypothesis that E-cadherin transient loss in DGC generates an acute phenotype of cell-cell and cell-matrix adhesion loss that persists and likely prevents spreading to distant sites; while transient or permanent E-cadherin loss in IGC likely triggers cell detachment and expression of alternative cadherins allowing spreading to distant organs.

**Financial support:** (1) FCT Fellowships SFRH/BD/113031/2015 to CP; (2) iFCT Program 2013 (IF/00615/2013) to GMA; (3) GenomePT project POCI-01-0145-FEDER-022184 to MF

**OC2-*CYP46A1*- gene therapy improves Machado-Joseph disease in mouse models**

Clévio Nóbrega^1,2,3#^, Liliana S. Mendonça^3#^, Adriana Marcelo^1,2^, Antonin Lamazière^4^, Sandra Tomé^3^, Gaëtan Déspres^4^, Carlos Matos^3^, Fatich Mechmet^1,2^, Dominique Langui^5^, Wilfred den Dunnen^6^, Luís Pereira de Almeida^3,7∗^, Nathalie Cartier^8∗^ and Sandro Alves^9∗^

1- Department of Biomedical Sciences and Medicine; 2- Centre for Biomedical Research, University of Algarve, Faro, Portugal; 3- Center for Neuroscience and Cell Biology, University of Coimbra, Portugal; 4- Laboratory of Mass Spectrometry, INSERM, Sorbonne Universités, Paris, France; 5- UMR S1127, and INSERM U1127, and CNRS UMR7225, and ICM, Sorbonne Universités, UPMC University of Paris, Paris, France; 6- University Medical Center Groningen, Department of Pathology and Medical Biology, Groningen, The Netherlands; 7- Faculty of Pharmacy, University of Coimbra, Portugal; 8- INSERM U1169 92265 Fontenay aux Roses and Université Paris-Sud, Université Paris Saclay, France; 9- Brainvectis Therapeutics, Paris, France; #, ∗ These authors contributed equally.

Aims/Context: Machado-Joseph Disease (MJD) is a neurodegenerative disease associated with extensive neuronal death. Defects in brain cholesterol metabolism may contribute to neurodegenerative diseases. Brain cholesterol is almost exclusively synthesized *in situ* and cannot cross the blood-brain-barrier. To maintain the cholesterol homeostasis, superfluous cholesterol is converted into 24S-hydroxycholesterol by the neuronal enzyme cholesterol 24-hydroxylase (*CYP46A1*). The present work evaluated i) whether *CYP46A1* levels are reduced in MJD, ii) if *CYP46A1* overexpression could improve MJD, and iii) the mechanisms behind the observed recovery. Methods: *CYP46A1* levels were evaluated in cerebellar extracts of MJD patients and in transgenic MJD mice cerebella. *CYP46A1* overexpression effect was assessed in two MJD mouse models. In the lentiviral-based mouse model, AAVrh10 encoding *CYP46A1* or GFP (control) were injected into the striatum of C57BL6/J mice, and 2 months post-injection the neuronal marker DARPP32 levels and mutant ataxin-3 (mutAtxn3) inclusions’ size and number were measured. Transgenic MJD mice were injected into the cerebellum with AAVrh10 encoding *CYP46A1* or GFP and motor performance was evaluated. Then mice's cerebella were analyzed for mutAtxn3 inclusions, Purkinje cell numbers, and cerebellar atrophy. Moreover, *CYP46A1* potential activation of autophagy was evaluated in Neuro2A cells and *in vivo.* Results: Our data indicate that *CYP46A1* cerebellar levels are decreased by 46% in MJD patients and by 29% in MJD mice. *CYP46A1* overexpression reduced DARPP32 loss (48%), mutAtxn3 inclusion number by 59% and their size by 47%. Significant alleviation of motor behavior impairments correlated with mitigation of MJD-associated neuropathology, namely, reduction of Purkinje cell loss (34%) and of cerebellar atrophy (25.40% in lobule X) was observed. Finally, our work demonstrated that *CYP46A1* overexpression induces autophagy (LC3B-II increase and p62 decrease) both in *in vitro* and *in vivo* MJD models. Conclusions: Overall our results demonstrate that *CYP46A1* overexpression improves MJD-associated motor coordination and neuropathology through autophagy enhancement.

**Financial support:** Work funded through the Regional Operational Program Center 2020, COMPETE 2020 and National Funds through FCT, PTDC/BTM-ORG/30737/2017 (POCI-01-0145-FEDER-030737), and by Brainvectis Therapeutics.

**OC3-The role of *CDH1* regulatory noncoding elements for E-cadherin expression**

São José Celina^∗1,2,3^, Monteiro R. Ana^∗1^, Qamra Aditi^4^, Acuna-Hidalgo R.^3^, Tan Patrick^4^, Mundlos S.^3,5,6^, Oliveira Carla^1,2^

1- i3S/Ipatimup, Institute of Molecular Pathology and Immunology at the University of Porto (Ipatimup), Porto, Portugal & Instituto de Investigação e Inovação em Saúde (i3S), University of Porto, Porto, Portugal; 2- Faculty of Medicine of the University of Porto, Portugal; 3- Max Planck Institute for Molecular Genetics, Berlin, Germany; 4- Cancer Science Institute of Singapore, NUS; 5- Institute for Medical and Human Genetics, Charité Universitätsmedizin, Berlin, Germany; 6- Berlin-Brandenburg Center for Regenerative Therapies (BCRT), Charité Universitätsmedizin, Berlin, Germany; ∗Equal Contribution

Introduction: *CDH1* pathogenic germline variants cause Hereditary Diffuse Gastric Cancer (HDGC), in less than half of patients/families fulfilling clinical criteria. *CDH1*-negative cases often display germline *CDH1* monoallelic expression and somatic E-cadherin loss of function. We hypothesized that *CDH1*-negative HDGC may arise due to germline defects in *CDH1* regulatory regions. Therefore, we explored the *CDH1* regulatory network to find expression modifier sequences, controlling *CDH1* expression, that could potentially explain E-Cadherin loss of function phenotypes. Materials and Methods: Capture Hi-C (cHi-C) with a viewpoint in *CDH1* promoter was performed in 5 gastric cancer cell lines, either positive or negative for E-cadherin expression. Mouse embryo reporter assays were used to test a candidate regulatory region by cloning in LacZ-reporter, integration into ColA1 locus of mouse embryonic stem cells, and generation of transgenic mice. Empty-vector mice were used as control for ColA1-driven expression. Tissue-specific β-galactosidase expression was tested in dissected tissues: stomach, esophagus, duodenum, liver (endoderm), heart (ectoderm) and skin (mesoderm). Results: We found evidence that *CDH1* promotor interacts simultaneously with an intergenic region in the short arm of chromosome 2 and an intronic region within *CDH1* intron 2. These interactions were specific of *CDH1*-negative cell lines, highlighting a potential negative regulatory network. We so far tested the regulatory potential of the *CDH1* intronic region and found tissue-specific β-galactosidase expression in endodermal-derived tissues (stomach, esophagus and duodenum), where E-cadherin exerts a primordial function. Conclusion: We found a potential negative regulatory network in gastric cancer cell lines through cis and trans interactions of the CDH1 promoter, and evidence for its tissue-specific regulation in the stomach. These findings suggest a novel mechanism triggering E-cadherin loss of function, worth to be tested in HDGC patients negative for CDH1 germline coding variants.

**Financial support:** 1) Solve-RD project, grant agreement No 779257 (European Union's Horizon 2020 research and innovation programme); 2) FEDER/COMPETE,“POCI-01-0145-FEDER-030164”; 3) FCT Fellowship, “SFRH/BD/140796/2018”

**OC4-Centrosome positioning and development of ciliopathies: role of the human centrosomal protein TBCCD1**

Bruno Carmona^1,2,3^, Carolina Camelo^2#^, Mehraz M^4^, Lemullois M^4^, David C. Ferreira^1,2^, Sofia Nolasco^3,5^, Lince-Faria M^6^, H. Susana Marinho^1,2^, Mónica Bettencourt-Dias^6^, Tassin AM^4^, Koll F^4^, Helena Soares^1,2,3^

1- Centro de Química Estrutural, Faculdade de Ciências da Universidade de Lisboa; 2- Centro de Química e Bioquímica, Faculdade de Ciências da Universidade de Lisboa; 3- Escola Superior de Tecnologia da Saúde de Lisboa, Instituto Politécnico de Lisboa; 4- Institute for Integrative Biology of the Cell (I2BC), CEA, CNRS, Université Paris Sud, Université Paris-Saclay; 5- Centro de Investigação Interdisciplinar em Sanidade Animal (CIISA), Faculdade de Medicina Veterinária, Universidade de Lisboa; 6- Instituto Gulbenkian de Ciência

Aims/Context: Primary cilia are specialized microtubule-based signaling organelles that convey extracellular signaling and cellular polarity into a cellular response. Defects in primary cilia assembly/function cause severe diseases known as ciliopathies, typified by clinical manifestations, as infertility, obesity, brain problems, blindness and kidney cysts. Primary cilia assembly entails centrosome migration to the plasma membrane where a centriole docks, maturates into a basal body (BB), and assembles the cilia axoneme. The human centrosomal TBCCD1 is a critical factor in centrosome positioning previously identified by us. Our aim is to discover the mechanisms/signals required for the correct positioning of the centrosome during cilia assembly, and how these mechanisms, when compromised, are related to ciliopathies. Methods: The proximity-dependent identification (BioID) assay was used to screen for TBCCD1 interactors. Immunofluorescent and super resolution microscopy, as well as Western blot, were used to study the levels and cellular localization of the identified TBCCD1 interactors in human RPE1 cells overexpressing or depleted of TBCCD1. To study the impact of TBCCD1 knockdown in motile cilia the ciliate *Paramecium*, containing ∼3,000 motile cilia, was used. Results: Our BioID screen for TBCCD1 interactors identified several well-known proteins encoded by ciliopathy genes, e.g. the centrosomal protein OFD1 involved in the Orofacio-Digital Syndrome. We show that TBCCD1 knockdown and overexpression in RPE1 cells affects OFD1 distribution. Super resolution microscopy shows TBCCD1 is localized at the distal region of the centrosome and that its depletion dramatically affects the centrosome subdistal protein CEP170, a component of cilia basal feet. In *Paramecium*, TBCCD1 knockdown causes abnormal BB-associated structures organization and anomalous BB positioning/anchoring defects. Conclusions: Our data support a role for TBCCD1 in the maintenance of centrosome structure and in BB anchoring at the cell membrane during ciliogenesis. TBCCD1 is emerging as a novel protein with a role in human ciliopathies.

**Financial support:** PEst-OE/QUI/UI0612/2013 and UID/MULTI/00612/2013 FCT; IPL/2019/MOONOFCILI/ESTeSL.

**OC5- Altered expression of imprinted genes and epigenetic regulators in placental tissue from intrauterine growth restriction**

Carla Caniçais^1,2,3^, Carla Ramalho^2,4^, C. Joana Marques^1,2^, Sofia Dória^1,2^

1- Department of Genetics, Faculty of Medicine, University of Porto, Porto, Portugal; 2- i3S – Instituto de Investigação e Inovação em Saúde, Porto, Portugal; 3- PhD Programme of Patology and Molecular Genetics, Instituto de Ciências Biomédicas Abel Salazar, University of Porto, Porto, Portugal; 4- Department of Obstetrics and Gynecology, Hospital São João, Faculty of Medicine, Porto, Portugal

Intrauterine growth restriction (IUGR) is a fetal growth condition characterized by the inability of the fetus to achieve its growth potential, which is dependent on normal placental function and development. Abnormal imprinted gene expression has been associated with abnormal placental development and function. The expression of these genes is controlled by epigenetic modifications, such as DNA methylation, at Imprinting Control Regions (ICRs). DNA hydroxymethylation was recently described and arises from the conversion of 5-methylcytosine into 5-hydroxymethylcytosine by the action of TET enzymes. The aim of this study was to evaluate the expression levels of imprinted genes and epigenetic regulators, DNA methylation at ICRs and global levels of DNA hydroxymethylation in placentas from IUGR pregnancies. Quantitative Real-Time PCR was performed in term placentas from 21 IUGR samples and 9 non-IUGR samples to evaluate the expression levels of seven imprinted genes (*PHLDA2, CDKN1C, KCNQ1, H19, IGF2, PEG10,* and *MEST*) and five epigenetic regulators (*DNMT1, DNMT3A, TET1, TET2* and *TET3*).

Additionally, standard bisulfite genomic sequencing and Combined Bisulfite and Restriction Analysis (COBRA) were performed to evaluate ICR2 (or KvDMR1) methylation. Global 5-hydroxymethylcytosine (5-hmC) was measured using the ELISA-based assay. *CDKN1C, PHLDA2*, and *PEG10* expression were significantly upregulated in placentas from IUGR, which also showed concomitant KvDMR1 hypermethylation. 5-hmC was present in both groups of placental tissue, with no significant changes between the two groups (IUGR vs controls). Overexpression of *CDKN1C* and *PHLDA2* genes, concomitantly with KvDMR1 hypermethylation, are consistent with fetal growth restriction since these genes negatively regulate growth. The global DNA hydroxymethylation analysis confirmed the presence of 5-hmC in the placenta, although levels did not differ in fetal growth restriction placentas. Our results suggest an important role for epigenetic modifications and modifiers in the control of human fetal growth.

**Clinical Research**

**OC6- Implementing mainstreaming genetic testing of *BRCA1/2* for cancer patients in a Portuguese tertiary hospital: preliminary results**

Mariana Soeiro e Sá^1^, Juliette Dupont^1^, Ana Berta Sousa^1^

1- Serviço de Genética Médica, Departamento de Pediatria, Hospital de Santa Maria, Centro Hospitalar Lisboa Norte, Centro Académico de Medicina de Lisboa, Lisbon, Portugal

Context: “Mainstreaming Cancer Genetics” is a clinical research programme first created at the Royal Marsden NHS Foundation Trust to provide a faster and less costly pathway to bring *BRCA1/2* testing directly to cancer patients through oncology appointments. Our aim was to implement a similar programme in our hospital. Methods: We defined patient selection criteria and designed a protocol for patient referral, informed consent, sample collection, and genetic testing. We centralized the process through Genetics, thus making sure we have knowledge of all patients included and their results. We promoted educational sessions for oncologists, prepared brochures for patients explaining the principles of genetic testing and possible results, and produced standard letters and clinical reports to accompany laboratory test results. Statistical analysis was done after codification.

Results: Since September 2018, 106 patients were referred, of whom 94 were included and 12 were excluded due to not meeting criteria. The most frequent inclusion criteria was breast cancer (BC) < 40 years (n = 32), followed by ovarian cancer (n = 18), and triple negative BC (n = 15). Of 86 available results, we identified 9 (10.5%) pathogenic variants (8 germinal/1 somatic) and 6 variants of unknown significance (VUS) (5 germinal/1 somatic). All patients in whom a variant was identified had a genetics appointment (to provide counseling and/or try to clarify the significance of VUS) within a mean time of 17.9 ± 15.8 days. The mean response time of genetic testing was 51.7 ± 9.8 days. The mean time from patient referral to final results (sent to the oncologist) was 67.7 ± 18.8 days. Conclusions: We significantly decreased our waiting time for urgent patients and lessened the burden of extra urgent appointments in our clinic, while providing patients with faster testing that might impact their treatment. In the future we will assess exactly what this impact was in patients and their families, the degree of satisfaction with the protocol (both from patients and oncologists) and the current classification of previously identified VUS

**OC7- The first genotype-phenotype study of European carriers of CDH1 germline mutations**

José Garcia-Pelaez^1^, Ana R. Monteiro^1^, Liliana Sousa^1^, Hugo Pinheiro^1^, Sergio Castedo^1,2,3^, Luzia Garrido^2^, Manuel Teixeira^4^, Geneviève Michils^4^, Vicent Bours^4^, Robin de Putter^4^, Lisa Golmard^4^, Maud Blanluet^4^, Chrystelle Colas^4^, Patrick Benusiglio^4^, Stefan Aretz^4^, Isabel Spier^4^, Robert Hüneburg^4^, Laura Gieldon^4^, Evelyn Schröck^4^, Elke Holinski-Feder^4^, Verena Steinke^4^, Daniele Calistri^4^, Gianluca Tedaldi^4^, Guglielmina N. Ranzani^4^, Maurizio Genuardi^4^, Catarina Silveira^4^, Inês Silva^4^, Mateja Krajc^4^, Ana Blatnik^4^, Srdjan Novakovic^4^, Ana Patiño-García^4^, José L. Soto^4^, Conxi Lázaro^4^, Gabriel Capellá^4^, Joan Brunet-Vidal^4^, Judith Balmaña^4^, Elena Domínguez-Garrido^4^, Marjolijn Ligtenberg^4^, Eleanor Fewings^4^, Rebecca C. Fitzgerald^4^, Emma R. Woodward^4^, Gareth Evans^4^, Helen Hanson^4^, Kristina Lagerstedt-Robinson^4^, Svetlana Bajalica-Lagercrantz^4^, Conceição Egas^4^, María I. Tejada^4^, Cristina Martínez-Bouzas^4^, Sonia Merino^4^, Sergio Carrera^4^, María García-Bercina^4^, Karin Dahan^4^, Damien Feret^4^, Nicoline Hoogerbrugge^4^, Marc Tischkowitz^4^, Carla Oliveira^1,3^

1- Ipatimup/i3 s, Institute of Molecular Pathology and Immunology at the University of Porto (Ipatimup), Porto, Portugal & Instituto de Investigação e Inovação em Saúde (i3S), University of Porto, Porto, Portugal; 2- Centro Hospitalar Universitário São João (CHSJ), Porto, Portugal; 3- Faculty of Medicine of the University of Porto (FMUP), Porto, Portugal; 4- ERN GENTURIS: The European Reference Network Genetic Tumour Risk Syndromes (ERN GENTURIS) consists of the following participating representatives and associated collaborators.

Introduction: Hereditary Diffuse Gastric Cancer-HDGC is caused by *CDH1* Pathogenic-P and Likely Pathogenic-LP germline variants, predisposing for diffuse gastric cancer-DGC and lobular breast cancer- LBC. *CDH1* variant carriers fulfilling HDGC criteria undergo intensive screening and/or preventive risk reduction gastrectomy/mastectomy while asymptomatic. We explored genotype-phenotype correlations to clarify the *CDH1*-associated disease spectrum and demonstrate the value of genetic-testing driven by phenotype and clinical-criteria. Materials and Methods: We collected/curated clinical-criteria and variant classification from 506 probands carrying coding and splice-site *CDH1* variants, from 10 EU Countries belonging to ERNGENTURIS. We registered 1361 phenotypes from 1302 family members. Results: 160/506 (31.6%) families fulfilled HDGC clinical criteria. While 87.5% of HDGC families carried P/LP actionable variants (96.4% truncating), only 13.3% (100% truncating) of those lacking criteria carried such variants (p = 10–5). The preferential phenotype in LP/P variant carriers, independently of fulfilling HDGC criteria, was GC (23% GC, 39% DGC). In contrast, the preferential phenotype in VUS carriers (94.4% missense) was BC (59% BC, 2% LBC). Among families carrying P/LP variants, DGC was always the most prevalent phenotype in all age ranges. Among LP/P variants carriers, there was an excess of DGC<40, and LBC>40 years old (p = 0.0003). In VUS and LB/B variant carriers, BC of undefined histotype was the major phenotype. No other relevant phenotypes were found in probands carrying LP/P variants and their relatives. Conclusion: This is the first genotype-phenotype and *CDH1* variant-driven study performed to date. It demonstrates that most carriers of truncating and clinically actionable *CDH1* variants fulfill HDGC clinical criteria, reinforcing their use in clinical practice. It establishes early onset DGC and LBC as the phenotypes associated with *CDH1* deficiency, and castoffs colorectal, ovarian and other GC and BC histotypes as part of the HDGC disease spectrum, supporting the need to expand clinical criteria to accommodate LBC.

**Financial support:** SolveRD/H2020-Grant/Ref. 779257; ERN-GENTURIS; Portuguese FCT/FEDER-COMPETE-POCI-01-0145-FEDER-030164.

**OC8-Management of Patients with Low-Penetrance Copy Number Variants – do they all need to see a Clinical Geneticist?**

Ana R Soares^1^, Simon Zwolinski^2^, Ana M Fortuna^1^, Miranda Splitt^2^

1- Genetics Department, Centro de Genética Médica Dr. Jacinto Magalhães, Centro Hospitalar Universitário do Porto, Portugal; 2- Northern Genetics Service, Institute of Genetic Medicine, Newcastle upon Tyne Hospital Foundation Trust, UK

Introduction: Since the introduction of Chromosomal Microarray as a first line test for children being investigated for neurodevelopment delay the number of patients referred to the Genetics Clinic with Low penetrance copy number variants (LP-CNVs) has increased dramatically. The aim of this study was to determine the outcome of clinical genetics assessment in children referred because of a LP-CNV with a view to developing best practice guidelines for referral to Genetics clinic. Methods: A retrospective analysis of the 10 most common LP-CNV referrals from 2016–2019. LP-CNVs were classified as inherited or de novo, not done or not available. In each case the LP-CNV was assessed in relation to the presenting phenotype as possibly causative, partially causative, not causative, incidental finding or carrier. Results: A total of 163 patients with LP-CNVs were included: 1q21.1q21.2 deletion -10; 1q21.1q21.2 duplication -23; 15q11.2 deletion -52; 15q13.2q13.3 duplication -10; 16p11.2 deletion -5; 16p11.2 duplication -8; 16p12.1 deletion -6; 16p13.11 deletion -14; 16p13.11 duplication -29; 22q11.21 duplication -10. Parental study was performed in 51 cases, not requested in 94 and not possible in 18 cases. The LP-CNV was considered to be a possible or partial cause in 128 (78.5%) and was not thought to be the cause in 27 cases (16.6%). In 18 cases (11%) a confirmed or suspected alternative diagnosis for the child's condition was made by the geneticist. Conclusion: Patients in which the LP-CNV was classified as possibly or partially causative had mild neurodevelopmental delay with or without a family history of mild learning difficulties. We propose that these patients could be managed by a ND Paediatrician and do not need referral to Clinical Genetics. The 18 children found have an additional genetic diagnosis all had various combinations of: moderate or severe neurodevelopmental delay, dysmorphic features, malformations, neurological or cutaneous signs. These clinical features should prompt referral to Clinical Genetics. These proposals would require specific training for Paediatricians, and good communication and teamwork between Paediatricians and Geneticists.

**OC9-Genome-wide characterization of a cohort of Alzheimer's patients from Iberia: a focus on rare variants**

Ana M Macedo^1,2,3^, Iva Gomes^1,2^, Sandra Martins^1,2^, Luis Durães^4^, Patrícia Sousa^4^, Manuel Figueruelo^5^, María Rodríguez^5^, Carmen Pita^5^, Miguel Rebelo^1,2^, Miguel Arenas^6^, Luis Álvarez^7^, Roberto Hornero^8^, Carlos Gómez^8^, Nádia Pinto^1,2,9^, Alexandra M Lopes^1,2^

1- Instituto de Patologia e Imunologia Molecular da Universidade do Porto (IPATIMUP), Porto, Portugal; 2- Instituto de Investigação e Inovação em Saúde (i3 s), Universidade do Porto, Porto, Portugal; 3- Faculdade de Ciências, Departamento de Matemática, Universidade do Porto, Porto, Portugal; 4- Associação Portuguesa de Familiares e Amigos de Doentes de Alzheimer, Porto, Portugal; 5- Asociación de Familiares y Amigos de Enfermos de Alzheimer y otras demencias de Zamora, Zamora, Spain; 6- Department of Biochemistry, Genetics and Immunology, University of Vigo; 7- TellmeGen, Valencia, Spain; 8- Biomedical Engineering Group, University of Valladolid, Valladolid, Spain; 9- Centro de Matemática da Universidade do Porto, Porto, Portugal

Aims/Context: To perform a genetic characterization of a cohort of late-onset Alzheimer's disease (LOAD) patients from Northern Portugal and Spain focusing on the spectrum of genome-wide rare variants (*MAF*). Methods: DNA was extracted from saliva and buccal swab samples and genotyped with Axiom Spain Biobank Array, which provides high coverage of whole-genome common and low frequency variation as well as Mendelian and functional alleles that are specific to Spanish. This analysis comprised 128 LOAD patients from Northern Portugal and from the Spanish autonomous community of Castile and León with a clinical diagnosis of AD. In addition, 59 controls (individuals over 65 years old with no signs of dementia or other brain disorders) from both regions were also analyzed. Rare variants in genes relevant for AD and with highly deleterious potential as assessed by CADD were prioritize for detailed annotation. Gene- based tests using SKAT-O and different models were performed to examine the aggregate effect of risk and protective variants. Results: In spite of the modest sample size, we detected a suggestive enrichment of rare variants in cases in several genes with functional links to AD. Overall, 16 AD genes harbored very rare pathogenic/likely pathogenic variants in our sample (cases and controls), thus making these variants much more frequent in our cohort than in control populations from GnomAD and ExAC. Conclusions: This study provides a characterization of genome-wide rare variation in AD in Castile and León and in Northern Portugal, the latter a region where dementing diseases are highly prevalent but still understudied. Even in a small sample of patients we were able to identify rare pathogenic variations and an excess of rare variants in cases in genes likely relevant to AD etiology, making this a valid approach to identify genes contributing to AD burden.

**Financial support:** Project 1317_AD-EEGWA, INTERREG, POCTEP (2014-2020). FCT (POCI-01-0145-FEDER-007274).FCT: CEECIND/00684/2017, IF/01262/2014, CEECIND/02609/2017, SFRH/BPD/97414/2013. RYC-2015-18241 (Spain)

**OC10- miRNA expression profile of plasma-derived extracellular vesicles distinguishes Machado-Joseph Disease patients from controls**

Magda M. Santana^1,2,4^, Patrick Silva^1,2,4^, Laetitia Gaspar^1,2,4^, Carina Henriques^1,4^, Joana Ribeiro^2^, Inês Cunha^2^, Rui Jorge Nobre^1,2,4^, Cristina Januário^3^, Luís Pereira de Almeida^1,4,5^, on behalf of European Spinocerebellar Ataxia Type 3/Machado-Joseph Disease Initiative (ESMI)

1- CNC - Centre for Neuroscience and Cell Biology, University of Coimbra, Coimbra, Portugal; 2- IIIUC - Instituto de Investigação Interdisciplinar, University of Coimbra, Portugal; 3- Coimbra Hospital and University Centre (CHUC), Coimbra, Portugal; 4- CIBB – Center for Innovative Biomedicine and Biotechnology, Coimbra, Portugal; 5 Faculty of Pharmacy,University of Coimbra, Coimbra, Portugal

Background: Machado–Joseph disease (MJD) is the most common dominantly-inherited ataxia worldwide. It is caused by a CAG over repetition in *ATXN3* gene, which translates into a mutated ataxin- 3 protein that accumulates in neurons, causing neuronal dysfunction and death. MJD leads to premature death and there is no therapy available. Potential therapeutic approaches have emerged, but the lack of large cohorts and biomarkers remain major barriers for the success of interventional studies. Extracellular vesicles (EVs) are enriched in specific small RNAs that are pointed out as promising biomarker candidates. Here, we established a Portuguese cohort of MJD patients that integrates the ESMI cohort and performed a transcriptomic analysis of EVs obtained from patients’ plasma to identify potential MJD biomarkers. Methods: The study was approved by Ethics Committee of Faculty of Medicine, University of Coimbra. Informed consent was obtained from all participants. Participants were characterized using clinical and functional tests. EVs were isolated by size exclusion chromatography. EVs size was characterized by nanoparticle tracking analysis; plasma contaminants by ELISA and RNA profile by automated electrophoresis. Small RNAseq was performed with an Illumina NextSeq system. Results: 48 patients were enrolled (26 males; 49.8 ± 13.6 years old). At baseline, mean score for Scale for the Assessment and Rating of Ataxia (SARA) was 13.6 ± 10.5. Follow-up data revealed a decrease in the performance of 8-meter walking test, but no variations from baseline in SARA. Obtained EVs exhibited sizes <150 nm, low levels of plasma contaminants and enrichment in small RNAs. MJD-EVs contained lower RNA levels than controls. Principal component analysis of the top 50 variable miRNAs present in plasma-derived EVs shows a segregated miRNA expressing profile between controls and patients. Conclusion: A Portuguese cohort of MJD patients was characterized and new biomarkers candidates were explored. miRNA expressing profile of plasma-derived EVs revealed novel blood-based candidates that distinguishes patients from controls having thus potential to be used as biomarkers for MJD.

**Financial support:** Co-financed by JPND and FCT (JPCOFUND/0001/2015; JPCOFUND/0005/2015), by COMPETE 2020 and Regional Operational Program Center 2020 national funds via FCT, under the projects CENTRO-07-ST24-FEDER-002006, BrainHealth2020 (CENTRO-01-0145-FEDER-000008), ViraVector (CENTRO-01-0145-FEDER-022095), POCI-01-0145-FEDER-007440, (POCI-01-0145-FEDER-029716), UID/NEU/04539/2013 and 01/BIM-ESMI/2016; by National Ataxia Foundation (USA), the American Portuguese Biomedical Research Fund (APBRF) and Richard Chin and Lily Lock Machado-Joseph Disease Research Fund.

**Clinical Cases**

**OC11- Koolen-de Vries syndrome – National Case Series with clinical and molecular characterization**

Marta P. Soares^1^, Márcia Rodrigues^1^, Juliette Dupont^1^, Ana Medeira^1^, João Freixo^2^, Sofia Nunes^2^, Isabel Cordeiro^1^, André Travessa^1^, Gabriela Soares^3^, Ana Fortuna^3^, Fabiana Ramos^4^, Joaquim Sá^4^, Susana Rocha^5^, Cristina Figueiredo^6^, Carla Mendonça^7^, Fernando Tapadinhas^7^, Rosário Silveira-Santos^1^, Sónia Custódio^1^, Ana Barreta^8^, Sílvia Serafim^9^, Hildeberto Correia^9^, Mariana Val^10^, Isabel M Carreira^10^, Paula Rendeiro^11^, Ana Sousa^1^, Ana Berta Sousa^1^

1- Serviço de Genética Médica, Departamento de Pediatria, Hospital de Santa Maria, Centro Hospitalar e Universitário Lisboa Norte, Centro Académico de Medicina de Lisboa, Lisboa; 2- Unidade de Genética Médica, Hospital de Dona Estefânia, Centro Hospitalar e Universitário de Lisboa Central, Lisboa; 3- Unidade de Genética Médica, Centro de Genética Médica Doutor Jacinto Magalhães, Centro Hospitalar e Universitário do Porto, Porto; 4- Serviço de Genética Médica, Hospital Pediátrico de Coimbra, Centro Hospitalar e Universitário de Coimbra, Coimbra; 5- Serviço de Pediatria, Centro Hospitalar Barreiro-Montijo, Barreiro; 6- Serviço de Pediatria, Centro Hospitalar de Setúbal, Setúbal; 7- Serviço de Pediatria, Unidade de Faro, Centro Hospitalar e Universitário do Algarve, Faro; 8- Serviço de Genética Médica, Laboratório de Análises Clínicas Dr. Joaquim Chaves, Miraflores; 9- Unidade de Citogenética, Departamento de Genética Humana, Instituto Nacional de Saúde Doutor Ricardo Jorge, Lisboa; 10- Laboratório de Citogenética e Genómica, Faculdade de Medicina, Universidade de Coimbra, Coimbra; 11- CGC Genetics, Centro de Genética Clínica, Porto

Introduction: Koolen-de Vries Syndrome (KdVS) is a rare genetic condition, caused by a 17q21.31 microdeletion, or a pathogenic variant in *KANSL1* gene. The clinical picture includes developmental delay (DD)/intellectual disability (ID) with expressive language particularly impaired, dysmorphisms, neonatal hypotonia, and friendly behaviour. Aim: To characterize at the molecular and clinical levels all patients in Portugal diagnosed with KdVS. Methods: 15 patients with a 17q21.31 deletion were identified in Portuguese Genetics Laboratories and Clinical Genetics Departments. Data were collected retrospectively by means of a questionnaire. Results: The deletion was detected in all cases by array-CGH, ranging from 431,7 to 987,4 Kb, and encompassed partially or completely *CRHR1, SPPL2C, MAPT, STH,* and *KANSL1* genes. Three patients had a maternally inherited deletion, including two siblings whose mother has low-grade somatic mosaicism. All individuals (age 3 to 37 years-old) had DD/ID and dysmorphisms. Hypotonia was reported in 8/11, motor delay in 6/8, and language/speech delay in 13/15. Brain malformations were present in 7/9 patients, and 4/6 had EEG abnormalities. Nine patients were overfriendly, and 6/11 had a neuropsychological disorder. The most common facial dysmorphisms were bulbous nose (9/10), long face (7/11), and narrow/short palpebral fissures, large/prominent ears, and broad chin (4/10). Congenital heart defects were present in 4/7, renal/urogenital anomalies in 7/12, visual and hearing problems in 8/10, musculoskeletal abnormalities in 7/10, and ectodermal anomalies in 7/11. Daily functioning was partially evaluated in 4/6 adult patients: all lived with family members, participated in family life, and were independent in daily living activities; 3 could read; 2 of them completed high-school, worked, used public transportation, could write a simple letter and read time. Conclusions: This study adds information to the molecular - one deletion was longer than typically reported in the literature - and clinical profile, including assessment of functioning in adults, of KdVS. It also points to the importance of parental segregation and mosaicism analysis.

**OC12- Pachydysostosis of the fibula in a case of Gardner Syndrome - a case report**

Daniela Oliveira^1^, Sofia Maia^1,2^, Inês Balacó^3^, Paulo Coelho^4^, Susana Almeida^5^, Margarida Venâncio^1,2^, Jorge Saraiva^1,6^, Gen Nishimura^7^, Sérgio B. Sousa^1,2^

1- Medical Genetics Unit, Hospital Pediátrico, Centro Hospitalar e Universitário de Coimbra, Portugal; 2- University Clinic of Genetics, Faculty of Medicine, Universidade de Coimbra, Portugal; 3- Paediatric Ortopedic Unit, Hospital Pediátrico, Centro Hospitalar e Universitário de Coimbra, Portugal; 4- Radiology Department, Centro Hospitalar e Universitário de Coimbra, Portugal; 5- Paediatric Gastroenterology Unit, Hospital Pediátrico, Centro Hospitalar e Universitário de Coimbra, Portugal; 6- University Clinic of Pediatrics, Faculty of Medicine, Universidade de Coimbra, Portugal; 7- Intractable Disease Center, Saitama Medical University Moroyama Campus, Japan

CONTEXT: Gardner Syndrome (GS) is a well-known variant of Familial Adenomatous Polyposis (FAP) caused by certain germline *APC* mutations. GS is characterised by prominent extra-colonic manifestations, namely skull and mandible osteomas, desmoid tumours, epidermoid cysts, fibromas on the scalp, shoulders, arms and back and dental abnormalities. Pachydysostosis of the fibula (PF) is a rare clinical entity characterised by unilateral bowing of the distal portion of the fibula and elongation of the entire bone, without affectation of the tibia. Unilateral bowing of the leg is relatively common, however, in most cases fibula and tibia are involved, being a clinical entity distinct from pachydysostosis. We describe a striking case of GS in which this life-saving diagnosis was unveiled by the multidisciplinary evaluation of skeletal findings: congenital PF and an osteoma. CASE REPORT: A 17-year-old male presented with a non-progressive bowing of the right leg detected at 18 months of age caused by a fibula malformation (later characterized as pachydysostosis) and a large exophytic osteoma of the left radius, noticed at the age of 15 years, without gastrointestinal symptoms. There was no relevant family history. Detailed clinical and radiological characterisation revealed multiple osteomas (of the left fibula, left ilium, metacarpals and mandible), skin lesions and dental abnormalities, raising the hypothesis of GS. This diagnosis was confirmed by genetic testing [c.4406_4409dup p.(Ala1471Serfs∗17) *de novo* mutation in the *APC* gene] and endoscopic investigation, which identified the presence of multiple adenomatous polyps throughout the colon, ileum and stomach. CONCLUSION: This case report expands the known phenotypic spectrum of skeletal manifestations of GS: this patient has a congenital fibula malformation, not previously associated with FAP, but which is likely to have been its first manifestation in this patient. This clinical case also illustrates the challenges in the early diagnosis of patients with GS, especially without family history, and highlights the importance of a multidisciplinary team and the adequate study of rare skeletal abnormalities.

**OC15- *MED13L*-related intellectual disability - National Case Series**

Raquel Gouveia Silva^1^, Oana Moldovan^1^, Joana Salgado^2^, Sérgio Sousa^2^, Célia Soares^3^, Gabriela Soares^3^, Patrícia Dias^1^, Diana Antunes^4^, Marta Amorim^4^, Fátima Lopes^5^, Patrícia Maciel^5^, Margarida Venâncio^2^, Joaquim Sá^2^, Ana Berta Sousa^1^

1- Serviço de Genética Médica, Departamento de Pediatria, Hospital de Santa Maria, Centro Hospitalar e Universitário Lisboa Norte, Centro Académico de Medicina de Lisboa, Lisboa, Portugal; 2- Serviço de Genética Médica, Hospital Pediátrico, Centro Hospitalar e Universitário de Coimbra, Coimbra, Portugal; 3- Unidade de Genética Médica, Centro de Genética Médica Doutor Jacinto Magalhães, Centro Hospitalar do Porto, Porto, Portugal; 4- Serviço de Genética Médica, Hospital Dona Estefânia, Centro Hospitalar e Universitário de Lisboa Central, Lisboa, Portugal; 5- Life and Health Sciences Research Institute (ICVS), School of Health Sciences, Universidade do Minho, Braga, Portugal

Introduction: *MED13L*-related intellectual disability syndrome is characterized by moderate to severe intellectual disability (ID), speech delay, hypotonia, behavioural issues, autism spectrum disorder, and dysmorphic features. To date, at least 70 cases have been reported. Methods: Clinical and molecular characterization of all patients from 4 Portuguese medical genetics departments with the proposed diagnosis of MED13L syndrome was performed retrospectively through a questionnaire completed by each referring clinician. Results: Twelve individuals from 9 families were included in this study. Mean age at diagnosis was 13.6 years (range 5–42). Neurodevelopmental features included moderate to severe ID (12/12), variable speech delay (6/12) or absent speech (6/12), motor delay (8/12), muscular hypotonia (3/12), behavioural problems (6/12), autistic features (2/12), and epilepsy (3/12). Other findings comprised hand/foot anomalies (5/12), cleft palate (4/12), structural heart defects (2/6), ophthalmological issues (6/11), and cerebral MRI findings (3/8). Skin and hair anomalies (2/12) and chronic constipation (2/12) were also reported and are not yet documented in the literature. All cases were isolated, except for four affected brothers. Molecular diagnosis was achieved by, exome sequencing in 5, ID-NGS panel in 1, arrayCGH in 3 patients and Sanger sequencing to look for a familial variant in the remaining 3. Five patients had heterozygous MED13L pathogenic SNVs (1 nonsense, 3 frameshift, 1 intronic deletion affecting splicing) and 3 had CNVs (1 deletion, 1 gene tetrasomy, 1 intragenic duplication). Six simplex cases underwent segregation analysis and were all *de novo*. In the familial case, the four affected individuals had a missense variant classified as a VUS. Both parents were clinically unaffected, only the mother was tested (negative); paternal analysis and further functional tests are necessary to assess pathogenicity. Discussion: The clinical picture of MED13L-related ID is relatively nonspecific and heterogeneous making the genomic approach with arrayCGH and WES/WGS the best option for diagnosis.

**Financial support:** One of the patients included was diagnosed through In2Genome project which is funded by Centro Portugal Regional Operational Programme (CENTRO-01-0247-FEDER-017800)

**OC17- Clinical and molecular characterization of neonatal inflammatory skin and bowel disease: the Portuguese panorama of a little-known syndrome**

Inês Margarido^1^, Oana Moldovan^2^, Teresa Kay^1^, Inês Carvalho^1^

1- Serviço de Genética Médica, Centro Hospitalar Universitário de Lisboa Central; 2- Serviço de Genética Médica, Centro Hospitalar Universitário de Lisboa Norte

AIMS: Epidermal growth factor receptor (EGFR) is a tyrosine kinase receptor important for cell proliferation and differentiation. The role of somatic *EGFR* mutations in some cancers is well known and led to the development of targeted therapies and improved survival. Germinative mutations are less well characterized. There are four patients described to date that developed severe neonatal skin and bowel inflammation. We aim to determine the current Portuguese prevalence and contribute to better portray the phenotype and prognosis of this syndrome. METHODS: We reviewed the literature and gathered data from the patients with germinative *EGFR* mutation described to date. We inquired all Portuguese medical genetics departments and collected phenotypic and molecular data from all cases with germinative *EGFR* mutation. RESULTS: There were 3 patients with homozygous c.1283G>A pathogenic variant in the *EGFR* gene in Portugal. The same variant was found in 3 patients in the literature. All our patients were female and descended from consanguineous parents. In the literature 2 patients were female, 2 were male, half of the total had a history of consanguinity. Pre-natal findings in both cohorts included polyhydramnios and intrauterine growth restriction; all patients were born pre-term with a median gestational age at birth of 30 weeks. The main clinical features in both groups were: severe ictiosis-like skin inflammation; alopecia; recurrent infections and sepsis; hypokalemia, hypomagnesemia and hypernatremia. No significant bowel inflammation was found in our patients. Median survival was 80 days and the most common cause of death was infection. CONCLUSION: By almost doubling the patients described we conclude that the main post-natal features of this syndrome are skin inflammation, recurrent infections, hypokalemia and hypomagnesemia. If a newborn presents with these symptoms, particularly with consanguineous parents, *EGFR* mutation should be considered. This is a potentially underdiagnosed entity and adequate molecular characterization might have a determinant role in predicting prognosis and for pre-natal counseling of future pregnancies.

**OC18-Spondyloepiphyseal dysplasia, Stanescu type: the clinical and molecular overlap of a very rare type II collagenopathy**

André M. Travessa^1^, Francisca Diaz-Gonzalez^2^, Teresa Mirco^3^, Filipa Ramos^4^, Karen E. Heath^2^, Ana Berta Sousa^1^.

1- Serviço de Genética Médica, Departamento de Pediatria, Hospital de Santa Maria, Centro Hospitalar Universitário Lisboa Norte, Centro Académico de Medicina de Lisboa, Lisbon, Portugal; 2- Institute of Medical & Molecular Genetics (INGEMM) and Skeletal dysplasia Multidisciplinary Unit (UMDE), IdiPAZ, Hospital Universitario La Paz, UAM, & CIBERER, ISCIII, Madrid, Spain; 3- Serviço de Medicina Física e de Reabilitação, Hospital de Santa Maria, Centro Hospitalar Universitário Lisboa Norte, Centro Académico de Medicina de Lisboa, Lisbon, Portugal; 4- Serviço de Reumatologia, Hospital de Santa Maria, Centro Hospitalar Universitário Lisboa Norte, Centro Académico de Medicina de Lisboa, Lisbon, Portugal.

Objectives/Context: Spondyloepiphyseal dysplasia, Stanescu type (SEDS), was first described in 1984. Until now, only 12 patients from four families have been reported, with some being first diagnosed with an unclassifiable spondyloepiphyseal dysplasia or progressive pseudorheumatoid dysplasia (PPD). SEDS was recently shown to be due to variants in COL2A1, classifying it as a type II collagenopathy. We aim to expand the spectrum of SEDS and to highlight the features that differentiate it from other overlapping skeletal dysplasias. Clinical case: We present an 8-year-old boy who was referred for genetic evaluation due to short trunk. Family history was unremarkable. Prenatal and neonatal periods were uneventful, and psychomotor development and growth were normal. He presented with limitation of cervical mobility due to C2-C3 fusion, and hand, foot, leg and thigh pain since 18 months of age. On physical examination, he had short trunk, stiffness and limited joint mobility, and waddling gait. Radiographs showed platyspondyly with anterior wedging and endplate irregularities, broad femoral necks with coxa valga, bulbous epiphyses of the short tubular bones, and large and flattened epiphyses of the long bones of the legs. Although the clinical picture suggested the diagnosis of PPD due to the absence of elongated femoral necks, a skeletal dysplasia multigenic NGS panel was performed and a heterozygous variant in COL2A1, c.620G>A, p.(Gly207Glu), was found. The diagnosis of SEDS was thus established. Conclusions: This case expands the clinical and mutational spectrum of SEDS. In fact, vertebral fusions were not previously described in SEDS. Moreover, this COL2A1 variant had not yet been associated with SEDS, but was interestingly reported in a family with other type II collagenopathy. The latter reinforces the importance of thorough phenotyping and clinical diagnosis in this group of disorders. SEDS clinically overlaps with PPD, thus multigenic skeletal dysplasia panels are a valuable resource. This case shows not all skeletal dysplasias present with short stature, thus any unexplained body disproportion warrants a comprehensive evaluation.

**Poster Presentations**

**Highlights**

**P1- Antisense transcription across the SCA37 locus and role in the disease**

Ana F. Castro^1,2^, Joana Loureiro^1,2^, Hugo Marcelino^2,3^, Joana Teixeira^2,3^, José Bessa^2,3,∗^, Isabel Silveira^1,2,∗,●^

1- Genetics of Cognitive Dysfunction Laboratory, IBMC - Instituto de Biologia Celular e Molecular; 2- i3S – Instituto de Investigação e Inovação em Saúde, Universidade do Porto; 3- Vertebrate Development and Regeneration Laboratory, IBMC - Instituto de Biologia Celular e Molecular; ^∗^ Equal contribution; ^●^ Corresponding author email: isilveir@ibmc.up.pt

Spinocerebellar ataxias (SCAs) are neurodegenerative diseases characterized by cerebellar atrophy and progressive motor impairment. In 2017, we established that SCA37 is caused by an (ATTTC)_n_ insertion into an (ATTTT)_n_ in a *DAB1 5’UTR* intron, resulting in RNA-mediated toxicity. Repeat expression in the *DAB1*-antisense strand, however, may contribute to SCA37 pathology as in other repeat diseases. In the *DAB1*-antisense strand, the (GAAAT)_n_ is in the middle poly(A) of an AluJb element, close to a CpG island and a cluster of transcription factor binding sites (TFBS). To investigate bidirectional transcription in SCA37, we performed *in vivo* reporter assays in zebrafish embryos, using a Tol2 transposon system. After injecting the normal sequence containing (AAAAT)_14_, CpG island and TFBS (FragT) upstream *EGFP*, we detected EGFP expression in zebrafish muscle and cerebellum. To map the position of the promoter(s), the FragT was divided in Frag1 (upstream of the repeat), Frag2 (AluJb) and Frag3 (downstream of the AluJb). Frag1 and Frag3 triggered EGFP expression in horizontal myosepta and muscle fibers, respectively. We detected the two transcription start sites by 5’RACE PCR, using the zebrafish transgenic lines. We, then, investigated interaction of FragT, 1, 2 and 3 with brain enhancers, cloning them upstream *EGFP* and a strong midbrain enhancer (*Z48*). The promoters in FragT, 1 and 3 enhanced EGFP expression in zebrafish midbrain, demonstrating interaction with Z48. To investigate whether the antisense repeat RNA is toxic, we injected (AAAAU)_7_, (AAAAU)_139_ and the insertion (GAAAU)_54_ RNAs in zebrafish embryos and assessed lethality rate. No significant increase in the lethality rate of embryos injected with the (GAAAU)_54_ RNA was observed compared with the other RNAs, 24 hr postfertilization. In summary, we identified two promoters that can be regulated by brain enhancers, in *DAB1*-antisense strand. Although the (GAAAU)_54_ insertion is not toxic *in vivo*, the antisense (AAAAT)_n_ is transcribed, suggesting that bidirectional transcription is involved in SCA37 pathology by an unknown mechanism.

**P2-Modeling the neurogenetics of neurodevelopmental disorders - hints from brain organoids**

Catarina M. Seabra^1,2,∗^, Ana Rafaela Oliveira^1,3,∗^, Joana R. Guedes^1,2^, Ana Luísa Cardoso^1,2^, Diana B. Sequeira^1,2,4^, Paulo J. Palma^4^, João Miguel Santos^4^, Frederico Duque^5,6^, Guiomar Oliveira^5,6^, João Peça^1,7^.

1- Center for Neuroscience and Cell Biology (CNC), Coimbra, Portugal; 2- Institute for Interdisciplinary Research (IIIUC), University of Coimbra, Portugal; 3- MIT Portugal PhD Program - NOVA University, Lisbon, Portugal; 4- Institute of Endodontics, Faculty of Medicine, University of Coimbra, Portugal; 5- Autism Unit of the Child Developmental Center and Clinical Research and Training Center, Pediatric Hospital, Coimbra Hospital and University Center, Coimbra, Portugal; 6- University Clinic of Pediatrics, Faculty of Medicine, University of Coimbra, Coimbra, Portugal; 7- Department of Life Sciences, Faculty of Science and Technology, University of Coimbra, Portugal

Background: Neurodevelopmental disorders (NDDs), such as autism and intellectual disability, affect more than 3% of children worldwide. Next generation sequencing has uncovered mutations at over 1000 loci, highlighting the extensive etiological variability of NDDs. This fact, combined with a heterogeneous clinical presentation, presents great challenges that range from delineating disease-associated molecular processes, to identifying effective therapies. 3D human brain organoids have revolutionized this field as they replicate key aspects of organogenesis. Brain organoids can be generated using patient's dental stem cells found in exfoliated teeth, presenting an opportunity for personalized approaches. In addition, organoids are amenable to gene editing which can be used to introduce rare mutations or test if their reversal restores neuronal function. Objectives: Our goal is to generate 3D brain organoids, derived non-invasively from human dental stem cells, to explore brain development in the context of specific gene mutations. Methods and Results: In collaboration with the Pediatric Hospital of Coimbra, we established the first biobank of dental stem cells to study NDDs in Portugal. Dental stem cells were analyzed using flow cytometry, qRT-PCR and immunocytochemistry and showed expression of Stro-1, Oct4 and Nestin. We differentiated dental stem cell-derived iPSC into brain organoids using the Lancaster et al. method. At 3 months, brain organoids expressed neuronal markers such as β3-tubulin and NeuN while at 6 months we identified subtype-specific neurons, such as dopaminergic, glutamatergic and GABAergic. We characterized specific time-points at which organoids show synaptic and neuronal network activity, using electrophysiology and calcium imaging, with reliable network function beyond 6 months of development. In parallel, we are performing gene editing via CRISPR/Cas9 to induce mutations in high risk genes. Conclusions: We show that dental stem cells are a suitable source to generate brain organoids and that 3D brain cultures are realistic models that will allow to understand the mechanisms behind NDDs and test for personalized therapeutic approaches.

**Financial supports:** Marie Skłodowska-Curie Actions - ProTeAN Project (Grant 799164); POCI-01-0145-FEDER-007440; BrainHealth 2020 CENTRO-01-0145-FEDER-000008

**P3-Investigating the role of *CD44v6* in Gastric Cancer: development of exon v6 skipping models by CRISPR/Cas9**

Silvana Lobo^1,2,3^; Carla Pereira^1,2^; Gabriela M. Almeida^1,2,4^; Carla Oliveira^1,2,4^

1- Instituto de Investigação e Inovação em Saúde; 2- Instituto de Patologia e Imunologia Molecular da Universidade do Porto; 3- Instituto de Ciências Biomédicas Abel Salazar; 4- Faculdade de Medicina da Universidade do Porto

Context/objective: Gastric cancer (GC) is the 5^th^ most common cancer and the 3^rd^ with the highest mortality. The standard of care for advanced disease is conventional chemotherapy with or without radiotherapy. We have shown that *de novo* expression of *CD44v6* is a poor prognosis factor in GC, but also a likely predictor of response to conventional chemotherapy. The cell adhesion molecule/gene *CD44* undergoes extensive alternative splicing, generating multiple isoforms, being *CD44v6 de novo* expression often associated with cancer aggressiveness. We aimed to develop exon v6 skipping models by CRISPR/Cas9 in CG cell lines, to explore the role of *CD44* variable exon v6 in response to therapy. Methods: We designed and established a pioneer strategy to produce the pleiotropy of *CD44* isoforms lacking specifically exon v6. We edited, by CRISPR/Cas9, two GC cell lines endogenously expressing *CD44v6* to specifically delete exon v6 from *CD44v6*-contaning isoforms, whilst maintaining the reading frame. v6-edited cell lines and corresponding *wild-type* controls were genotyped and characterized for *CD44* expression patterns and then treated with cisplatin and 5-Fluorouracil. Cell survival was evaluated in short-term treatments by PrestoBlue and Sulforhodamine B assays and long-term treatments by clonogenic assay. Results: We obtained 10 independent clones of homozygously edited GC cell lines lacking exon v6, while maintaining expression of the remaining portions of *CD44* variant isoforms. These were characterized and all sequenced transcripts mimicked exon v6 skipping where exon v5 was in-frame with v7 in the mRNA. Preliminary results from drug treatments indicate that there are no differences in survival of cell lines without exon v6 when compared to wild type cells. Discussion/conclusion: We successfully designed a strategy that mimics exon v6 skipping and generated two cell lines expressing *CD44* isoforms lacking exon v6. Although preliminary data is not conclusive regarding response to conventional therapy, these models are crucial to disclose the role of exon v6, that is responsible for binding to *c-MET* and *VEGFR-2* oncogenes in human aggressive cancers.

**Financial support:** FEDER/COMPETE 2020/POCI/Portugal 2020/FCT project - POCI-01-0145-FEDER-007274); NORTE 2020/PORTUGAL 2020/ERDF project - NORTE-07-0124-FEDER-000029; PTDC/CTM-NAN/120958/2010, from FCT; CP supported by grant SFRH/BD/113031/2015 from FCT. GMA supported by iFCT/POPH-QREN Type 4.2, EDF and MCTES (IF/00615/2013).

**P5-*VCAM1* modulation on endothelial cells – implications for vasculopathy in sickle cell anemia**

Marisa Silva^1^, Sofia Vargas^1^, Andreia Coelho^1^, João Lavinha^1,2^, Paula Faustino^1,3^

1- Departamento de Genética Humana, Instituto Nacional de Saúde Doutor Ricardo Jorge, Lisboa; 2- BioISI, Faculdade de Ciências, Universidade de Lisboa, Lisboa; 3- ISAMB, Faculdade de Medicina, Universidade de Lisboa, Lisboa, Portugal.

Sickle cell anemia (SCA) is a highly heterogeneous and multifactorial-like monogenic disease that arises from homozygosity for the c.20A>T mutation in the *HBB* gene. Vascular disease is systemic in SCA, with profound effects in organs like the brain, where stroke is the most severe end of the cerebral vasculopathy (CVA) spectrum. Endothelial dysfunction plays a major role in vasculopathy with several adhesion molecules, such as VCAM-1, being produced by the endothelium altered as a response to inflammatory cytokine (e.g., TNF-α) signalling. In previous association studies, we found positive associations between the presence of four specific *VCAM1* gene promoter haplotypes and i) high blood flow velocities in the median cerebral artery, and ii) a chronic hemolysis biochemical marker. In this study, we aimed to assess the functional role of those *VCAM1* promoter haplotypes in endothelial cell response following endothelial activation through TNF-α stimulation. After molecular cloning of 3 haplotype constructs, using a pGL4 promoterless vector, haplotype sequence was confirmed, by Sanger sequencing, prior to transfection. We used EAhy926, HUVEC and HBEC as different endothelial cell models, and performed transfection experiments for each construct, with and without TNF-α stimulation. Differences in promoter activity were assessed by luciferase reporter assay. All haplotypes showed differences in promoter activity, after TNF-α stimulation, in all cell models. Haplotype 1 showed decreased promoter activity, while haplotypes 7 and 8 showed increased activity after TNF-α stimulation, in all cell models. These results are consistent with lower *VCAM1* expression due to haplotype 1, and therefore a protective effect. Conversely, a higher expression due to haplotypes 7 and 8, suggests an increased vasculopathy risk, in a pro-inflammatory milieu. The association between specific haplotypes and endothelial cell response further enhances the modifier effect of *VCAM1* on endothelial dysfunction and its impact in SCA pathophysiology, as well as its potential role as a biomarker of SCA vasculopathy risk, severity and prognosis.

**Financial support:** This work was partially supported by INSA_202DGH720 project.

**P6-Contribution of rare cryptic deletions to severe spermatogenic impairment – insights from a large cohort of azoospermic men (GEMINI)**

Alexandra M. Lopes^1,2∗,^ Liina Nagirnaja^3,4^, Filipa Carvalho^1,5^, João Gonçalves^6^, Susana Fernandes^1,5^, Kristian Almstrup^7,8^, Ewa Rajpert-De Meyts ^7,8^, Susana Seixas^1,2^, Brendan Houston^9^, Alberto Barros^1,6^, Moira O’Bryan^9^, Kenneth I. Aston^10^, Donald F. Conrad^3,4^ on Behalf of the GEMINI Consortium^11^

1- i3S - Instituto de Investigação e Inovação em Saúde, University of Porto, Portugal; 2- IPATIMUP - Institute of Molecular Pathology and Immunology, University of Porto, Portugal; 3- Dep. of Genetics, Washington University School of Medicine, St. Louis, USA; 4- Dep. of Pathology and Immunology, Washington University School of Medicine, St. Louis, USA; 5- Dep. de Genética, Faculdade de Medicina da Universidade do Porto, Portugal; 6- Dep. de Genética Humana, Instituto Nacional de Saúde Dr. Ricardo Jorge, Lisboa, Portugal; 7- Dep. of Growth & Reproduction, Rigshospitalet, Denmark; 8- University of Copenhagen, Denmark; 9- The School of Biological Sciences, Monash University, Australia; 10- Dep. of Surgery, University of Utah, USA; 11- GEMINI ∗email: alopes@ipatimup.pt

Aims/Context: Azoospermia, the most severe form of male infertility, affects approximately 1% of men worldwide and in the great majority of the cases the aetiology of the disease remains unidentified. As an ongoing effort to characterize the genetic architecture of male infertility, the Genetics of Male Infertility Initiative (GEMINI) consortium funded by the NIH has now sequenced the whole exome of 927 well phenotyped NOA (non-obstructive azoospermia) cases from 6 countries and 84 men with normal sperm parameters, of which 375 are Portuguese (299 patients and 76 controls). Methods: CNV calling was successfully performed in 693 NOA and 76 normozoospermic controls using XHMM (eXome-Hidden Markov Model) and following functional annotation the most interesting deletions were validated by other methods. Results: Likely causal deletions were found in 12 patients (overlapping genes previously associated with male infertility and not present in controls) and putative causal deletions in 29 (overlapping genes important for spermatogenesis in mouse). Most of the genes deleted only in NOA were patient-specific (∼60%). Conclusions: Although CNV calling from exome data has less power than from whole-genome it can contribute to the identification of genes affected in genetically heterogeneous diseases such as NOA.

**Financial support:** This study was funded by grant 1R01HD078641-01A1 to Don Conrad and Kenneth Aston, Washington University, St Louis, MO. AML is funded by FCT (research contract IF/01262/2014).

**P7-Protein and Copy Number Evaluation in Head and Neck Cancer patients**

Luísa Esteves^1^, Ilda P Ribeiro^1,2^, Sandra I. Anjo^3,4^, Leonor Barroso^5^, Francisco Marques^2,6,7^, Bruno Manadas^3^, Isabel M Carreira^1,2^, Joana Barbosa de Melo^1,2^

1- Cytogenetics and Genomics Laboratory, Faculty of Medicine, University of Coimbra, 3000–354 Coimbra, Portugal; 2- CIMAGO - Centre of Investigation on Environment Genetics and Oncobiology - Faculty of Medicine, University of Coimbra, 3000–354 Coimbra, Portugal; 3- CNC - Centre for Neuroscience and Cell Biology, University of Coimbra, Portugal; 4- Faculty Medicine, University of Coimbra, Portugal; 5- Maxillofacial Surgery Department, Coimbra Hospital and University Centre, CHUC, EPE, 3000–075 Coimbra, Portugal; 6- Department of Dentistry, Faculty of Medicine, University of Coimbra, 3000–075 Coimbra, Portugal; 7- Stomatology Unit, Coimbra Hospital and University Centre, CHUC, EPE, 3000–075 Coimbra, Portugal.

Context: Head and neck squamous cell carcinoma (HNSCC) is a cancer of the upper aero digestive tract that arises from several cumulative molecular and genomic alterations with aggressive clinical courses, poor prognosis and a high potential for metastization and relapse. The clinical use of a biomarker or set of biomarkers to predict the clinical outcomes of HNSCC patients could affect both the patient's prognosis and the type and duration of the treatment. Protein biomarker discovery and their correlation with genomic alterations for the early detection of metastasis or relapses is a crucial unmet need to improve the clinical outcome of these patients. Methods: Forty tumour samples from a previously studied cohort at the copy number variation (CNV) level, were analysed using information-dependent acquisition (IDA) of pooled samples for protein identification and SWATH-MS acquisition of each individual sample for protein quantification. A Mann-Whitney U test was implemented for the obtained proteins, taking into account the metastasis/relapse status of the patients and a classification model was built using the resulting proteins (p < 0.05) and some clinical features. The protein levels were correlated with the corresponding genomic alterations. Results: The logistic regression model was constructed based on the clinical staging and three proteins related with stem cell population maintenance, fatty acid elongation and extracellular matrix composition, with 87.5% accuracy in predicting the outcome of the patients in what comes to the development of relapse or metastasis. The area under the ROC curve was 0.864, with a 95% CI of [0.766;0.962], rendering it a model with good class separation ability. Conclusions: We were able to identify a set of proteins that seem to have a good prediction ability in what comes to metastasis and relapse status of the patients and correlate them with the CNVs. This approach has the potential to identify biomarkers with diagnostic and prognostic value leading the way to a new era of personalized medicine, that may help with metastasis and relapse early detection and consequently improve the clinical outcome of these patients.

**P8-The bigger the better? Evaluation of the value of large multi-gene panels in Portuguese cardiomyopathy genetic testing**

Márcia Baixia^1,2^, Sónia Sousa^1,2^, Maria J. Pina^1,2^, Raquel M. Silva^1,2^, Luis Cirnes^1,2,4^, Paulo Canedo^1,2^, Sérgio Castedo^1,2,3^, José C. Machado^1,2,3^

1- Institute of Molecular Pathology and Immunology of the University of Porto (IPATIMUP), Porto, Portugal; 2- Institute for Research and Innovation in Health (i3S), University of Porto, Rua Alfredo Allen, Porto, Portugal; 3- Faculty of Medicine, University of Porto, Alameda Prof. Hernani Monteiro, Porto, Portugal; 4- Escola Superior de Saúde (ESS), Instituto Politécnico do Porto (IPP), Porto, Portugal

Introduction: Genetic testing of cardiomyopathies went through major changes in the last few years, from sequential Sanger sequencing of the most likely gene candidates, to multi-gene panels by NGS, with an ever increasing number of genes analyzed. Since only a few genes account for the majority of hereditary cardiomyopathies, the increase in the number of genes evaluated is largely accompanied by adding less relevant or penetrant genes to existing panels, which may translate in minor benefits in terms of diagnostic yield but a significant increase in the number of variants of uncertain significance (VUS). In order to access the pros and cons of larger gene panels, the results of different cardiomyopathy gene panels used in our laboratory were reviewed, taking into account current ACMG classification criteria. Methods: All results of different cardiomyopathy panels performed between 2011 and 2018 at Ipatimup Diagnostics were retrieved (n = 1781 index cases). We calculated the diagnostic yield of each gene panel at the time they were used in the laboratory. Moreover, we compared the results before and after applying ACMG guidelines. Results: Before ACMG guidelines were adopted, a case was considered positive whenever a rare variant was identified. With the adoption of the ACMG guidelines, several variants previously considered relevant were classified as VUS, which lead to a drop in the diagnostic yield of the test (from 68% in 2011 to 37% in 2018). This drop is even increasing over time, as a result of the adoption of ever larger gene panels. Conclusions: The increase in the number of genes in cardiomyopathy gene panels does not necessarily mean an increase in the diagnostic yield of genetic tests. There is an increment in the number of variants detected, however most of them are VUS, some of which in genes of current limited value for cardiomyopathy genetic testing. Nevertheless, if we look at genetic testing as a tool to better understand a disease, the study of these variants (namely with functional assays and segregation studies) might help in the future to better understand some cases, which remain uncertain with the current available information.

**Financial supports:** Own (Institutional - Ipatimup) financial support.

**P9-Characterization of copy number variants (CNVs) identified by genetic testing of inherited retinal disorders**

Rocío Sánchez-Alcudia^1^, Lucia Guidugli^2^, Miika Mehine^1^, Sari Tuupanen^1^, Kati Kämpjärvi^1^, Kirsty Wells^1^, Johanna Känsäkoski^1^, Miko Valori^1^, Inka Saarinen^1^, Mikko Muona^1^, Eeva-Marja Sankila^1^, Juha W Koskenvuo^1^, Tero-Pekka Alastalo^2^

1- Blueprint Genetics, Helsinki, Finland; 2- Blueprint Genetics, San Francisco, California, United States

Aims: Retinal dystrophies (RD) include a heterogeneous group of disorders that damage the photoreceptors in the retina causing visual impairment. Prompt genetic diagnosis of these disorders can assist in risk assessment measures, management of symptoms, and selection of the appropriate targeted treatment. To provide a comprehensive diagnosis, the genetic testing strategy needs to take into account sequence alterations as well as copy number variants (CNVs). The aim of this study was to evaluate the rates and characteristics of CNVs in a cohort of 2754 patients tested using a comprehensive RD panel. Methods: DNA from patients was sequenced by targeted OS-Seq using the Illumina NextSeq500 sequencing platform or the IDT xGEN Exome Research Panel using the Illumina NovaSeq platform. CNVs were detected by CNVkit and an in-house developed deletion caller. Results: CNVs in a total of 47 genes matching the patient's phenotype were reported as a primary finding in 128 out of 2754 (4.6%) cases. Of these, 91 (71.1%) were partial gene deletions, 17 (13.3%) whole gene deletions, four (3.1%) one exon deletions, and one (0.8%) partial exon deletion. In addition, ten (7.8%) partial gene duplications, three (2.3%) whole gene duplications, one (0.8%) whole gene gain and one (0.8%) partial gene gain (CN>3) were identified. The majority of CNVs (113, 88.3%) were either likely pathogenic or pathogenic while 15 (11.7%) were variants of uncertain significance. Of the likely pathogenic and pathogenic CNVs, 94 (73.4%) were diagnostic: 66 (70.2%) in autosomal recessive genes, 17 (18.1%) in autosomal dominant genes, and 11 (11.7%) in X-linked genes. The *USH2A* and *PRPF31* genes were enriched in CNVs compared to other genes. Notably, CNVs were identified also in genes in which CNVs are not commonly reported, e.g. *ABCA4* and *RPE65*. Conclusions: Overall, these results highlight the importance of a comprehensive genetic testing approach for the diagnosis of retinal dystrophies. We have identified CNVs ranging from one exon deletions to whole gene deletions in multiple genes. In addition, we have detected a relative high percentage of copy number duplications that warrant further investigation.

**P10-Characteristics of a Portuguese cohort of Li-Fraumeni patients**

Sofia Fernandes^1,3^, Sofia Fragoso^2^, Bruno Filipe^2^, Sidónia Santos^2^, Ana Opinião^1^, Ana Clara^1^, Sandra Bento^1^, Ana Luís^1^, Isália Miguel^1^, Cecília Moura^1^, Pedro Louro^1,4^, Fátima Vaz^1,2^

1- Familial Risk Clinic, Instituto Português de Oncologia de Lisboa Francisco Gentil, Lisboa, Portugal; 2- Molecular Pathobiology Research Unit, Instituto Português de Oncologia de Lisboa Francisco Gentil, Lisboa, Portugal; 3- Medical Genetics Unit, Hospital Pediátrico, Centro Hospitalar e Universitário de Coimbra, Coimbra, Portugal; 4- Faculty of Health Sciences, Universidade da Beira Interior, Covilhã, Portugal

Context: Li-Fraumeni Syndrome (LFS) is an autosomal dominant condition caused by heterozygous germline pathogenic variants in *TP53* gene, which encodes a tumor suppressor protein. LFS is a cancer predisposition syndrome associated with the early development of cancers, such as sarcomas, brain tumors, leukemias, breast and adrenocortical carcinomas. Clinical diagnosis is usually based on classic or Chompret criteria. Patient testing and management is still challenging. Methods: Clinical and molecular characterization of all LFS cases observed at Familial Risk Clinic, IPO Lisboa. Patient and family's medical records were reviewed. Results: We identified 16 patients (4 males, 12 females) from 12 unrelated families, each with a different TP53 variant. Four families meet the classic criteria, 6 the Chompret criteria and 2 neither of these. From 2007 to 2016, affected patients were identified mostly by Sanger sequencing, while 7/12 cases were recently diagnosed through multigene testing for breast/ovarian cancer. Three patients were identified by predictive testing. We found 3 likely pathogenic and 9 pathogenic variants (2 hotspots and 1 Brazilian founder variant). As expected, the majority are missense (7/12) and 2/7 with known dominant-negative effect. Our patients had a first tumor at median age of 28 (1–59yrs) and were diagnosed with a median of 2 primary cancers (1–4). Childhood tumours were reported in 7/12 families, being diagnosed at median age of 8.5 (1–17yrs). One family had breast cancer as the only cancer diagnosis (6 cases). We also report a probable TP53 mosaic, in a 57yrs patient with fallopian tube endometrioid carcinoma. Discussion: TP53 pathogenic or likely pathogenic variants have been identified in individuals who don’t meet classical criteria for LFS, mainly by multigene panel testing for breast cancer. This raises important questions: is LFS prevalence underestimated in the clinic? Should we go for reverse phenotyping in a condition where patient management is still not consensual? Additional data from this and other cohorts of LFS patients will be important for genotype-phenotype correlation, better-informed genetic counselling and patient managemen.

**P11-ZTTK syndrome: first Portuguese case of a recently described syndrome?**

Marta Marques^1^, Sofia Maia^1,2^, Fabiana Ramos^1^, Jorge M. Saraiva^1,3^

1- Medical Genetics Unit, Hospital Pediátrico, Centro Hospitalar e Universitário de Coimbra, Coimbra, Portugal; 2- University Clinic of Genetics, Faculty of Medicine, University of Coimbra, Portugal; 3- University Clinic of Pediatrics, Faculty of Medicine, University of Coimbra, Portugal

Introduction: *SON* gene is highly conserved in mammals and encodes a protein containing an arginine/serine rich domain and two RNA-binding motifs playing an important role in regulating multiple cellular processes such as cell cycle, chromatin remodeling and genome stability. Recently described *SON* heterozygous mutations, all predicted to result in loss of protein function, were associated with ZTTK syndrome (MIM #617140). The phenotype of the 28 described cases in the literature overlap with established spliceosomal disorders and this gene seems to be a critical gene in microdeletions encompassing 21q22.11. Case description: We present, to our knowledge, the first molecularly confirmed Portuguese case with ZTTK syndrome in a boy with global developmental delay, hypotonia, feeding difficulties, short stature, brain malformations, congenital heart defect and facial dysmorphisms. Our patient was first seen at the age of 2 years and fragile X syndrome mutation analysis and array-CGH were performed and had normal results. At the age of 4.5 years we reevaluated the patient and a next-generation sequencing panel of 6110 genes was performed. The *de novo* pathogenic *SON* (NM_138927.2) c.5753_5756del p.(Val118Glufs∗87) variant in heterozygosity was identified, establishing the diagnosis. This 4-bp deletion seems to be a recurrent variant, which has been already reported in the literature in seven patients and was proven to be *de novo* in all described cases.

**P12-Copy number variations analysis of NGS data in germline oncology testing**

Maria Caloba^1,2^, Viviana Silva^1^, Rita Cerqueira^1^, Jorge Pinto Basto^1^

1- Laboratório de Diagnóstico Molecular e Genética Médica, CGC Genetics/Unilabs, Porto, Portugal; 2- Universidade de Trás-os-Montes e Alto Douro

Aims: Establish CNV detection using NGS data as part of diagnostic analysis for germline oncology genetic testing. Context: Genetic variation in human genome can range from large chromosomal abnormalities to single nucleotide variations (SNVs), including structural variations, copy number variations (CNVs), small indels and simple base alterations. CNVs comprise gains or losses of genomic material (duplications or deletions, respectively) that directly influence genetic dosage which have direct implications in inherited diseases. Copy number information can be obtained from NGS data, allowing detection of duplications and deletions of genomic regions in a single study. This study focus on patients with suspected hereditary cancer tested for different oncology gene panels, including CNVs analysis in a routine workflow. Methods: After software validation, CNVs analysis was performed on 902 clinical samples tested for oncology NGS panels. Copy number variations reported were confirmed by other methods (MLPA or qPCR) and the diagnostic yield was calculated. Results: A total of 902 patients were tested and 237 had relevant single nucleotide variants and 22 had gross deletions/duplications. From 22 CNVs reported 19 were deletions and 3 were duplications. The global diagnostic yield was 28.7%, 26.3% for SNVs and 2.4% for CNVs; these lines up or even slightly above to the referenced in literature (1.7%). Conclusions: CNVs detection through NGS data is an addition tool that allows accurate detection of large rearrangements and increases diagnostic yield by 2.4% which is relevant for clinical management and genetic counseling to patients and their relatives.

**Basic Research**

**P14-A multiplex DNA methylation SNaPshot assay for age prediction using blood samples: a replication study**

Helena Correia Dias^1,2,3^, Janet Pereira^4^, Catarina Pinto^4^, Eugénia Cunha^2,3^, Francisco Corte Real^3,5^, Licínio Manco^1^

1- Research Centre for Anthropology and Health (CIAS), Department of Life Sciences, University of Coimbra, Portugal; 2- Centre for Functional Ecology (CEF), Laboratory of Forensic Anthropology, Department of Life Sciences, University of Coimbra, Portugal; 3- National Institute of Legal Medicine and Forensic Sciences, Portugal; 4- Department of Haematology, Centro Hospitalar e Universitário de Coimbra (CHUC), Portugal; 5- Faculty of Medicine, University of Coimbra, Portugal

Background: Many age-dependent DNA methylation markers have been identified in various tissues and body fluids, using methodologies such as bisulfite pyrosequencing, massive parallel sequencing or SNaPshot analysis. In a previous study [1], five CpG sites located in *ELOVL2, FHL2, KLF14, C1orf132/MIR29B2C* and *TRIM59* genes, were tested for age prediction purposes in blood, saliva and buccal swab samples using a multiplex methylation SNaPshot assay, showing accurate age prediction in blood. Aim: To replicate the multiplex methylation SNaPshot assay of Jung et al. [1] in blood samples of Portuguese healthy individuals.

Methods: Fifty nine blood samples (37 females, 22 males; aged 1–94 years-old) were evaluated using the SNaPshot method, which consisted of multiplex PCR followed by a multiplex SBE (single-base extension) reaction. The specific primers were those previously described [1]. Linear regression models were used to analyze relationships between methylation levels and chronological age using IBM SPSS software v.24. Results: Among the five markers, the CpG site in the *ELOVL2* showed the strongest correlation between DNA methylation and age (R = 0.951, p = 3.58e-29), following by *FHL2* (R = 0.946, p = 1.49e-29), *C1orf132/MIR29B2C* (R = -0.924, p = 1.67e-25) and *TRIM59* (R = 0.910, p = 2.04e-23). *KLF14* showed the lowest age correlation value (R = 0.791, p = 1.57e-13). The final age prediction model, using simultaneously the 5 CpG sites, showed a high correlation coefficient (R = 0.982), highly significant (p = 3.63e-34), explaining 96.4% of age variation. A strong correlation between predicted and chronological ages was observed (Spearman correlation coefficient, r = 0.971), with a mean absolute deviation (MAD) between predicted and chronological ages of 4.27 years. Conclusions: The multiplex methylation SNaPshot assay revealed high prediction accuracy in blood, showing to be useful in forensic analysis for age estimation.

**References:** 1. Jung SE et al. DNA methylation of the ELOVL2, FHL2, KLF14, C1orf132/MIR29B2C, and TRIM59 genes for age prediction from blood, saliva, and buccal swab samples. Forensic Sci Int Genet. 2019; 38:1–8

**Financial support:** HCD has a PhD grant from FCT (SFRH/BD/117022/2016).

**P15-Exploring the association between Y-chromosome lineages and***G6PD***African mutations in Portuguese patients**

Ugnė Kaminskaitė^1^, Licínio Manco^1^

1- Research Centre for Anthropology and Health (CIAS), Department of Life Sciences, University of Coimbra

Background: Glucose-6-phosphate dehydrogenase (G6PD) deficiency results from mutations on *G6PD* gene on chromosome Xq28. In Portuguese patients, the two most common mutations are *G6PD* A- (202G>A) (63.4%) and *G6PD* Betica (968T>C) (14.1%) observed in the context of the common sub-Saharan African A (376G) haplotype. Y chromosome lineages were previously identified in Portugal, being the typical Western European haplogroup R1b-M269 the most common (∼60%), following by haplogroups E-M35 (∼12%) and J-M304 (∼10%). Aim: To address the association between Y-chromosome haplogroups and *G6PD* African mutations to determine whether there is signs of African male lineages assimilation in Portugal. Methods: DNA samples from 34 male *G6PD* deficient Portuguese patients, without known black ancestry, with mutations *G6PD* A-, and *G6PD* Betica, were analysed for Y-chromosome haplogroup identification using binary markers. The internal variation of the male lineages was evaluated by analysis of 7 Y-STRs. Results: Y genotyping of the *G6PD* patients revealed 6 larger haplogroups (18 R1b-M269; 7 E1b1b1- M35; 6 J-M304; 1 I-M170; 1 L-M22; 1 K∗-M9), all previously reported in the general Portuguese population. No common sub-Saharan African haplogroups were found. The multidimensional scaling (MDS) plot based on a pairwise RST matrix from seven Y-STR loci, distributed 14 populations in three main groups comprising European, Middle East and North African populations. The *G6PD* group was strong clustered with Europeans, and a sub-Saharan African population is clearly separated from all other populations. The E1b1b1a-M78 sub-haplogroup (total frequency 11.8%), which is widely distributed in northern and eastern Africa, Europe, and western Asia, but was also found in sub-Saharan Africa (1 in 883 samples), was the most common within E1b1b1-M35 (4 in 7 samples). The MJ network of E-M78 showed 2 samples identified with Spanish haplogroups and 2 isolated haplogroups, one of these at one mutational step from the sub-Saharan African haplogroup. Conclusions: Male lineages from Portuguese *G6PD* deficient patients do not show signals of Sub-Saharan African ancestry, except for one E-M78 haplogroup.

**P17-Cis-regulatory similarities of the human and zebrafishpancreas identify diseases related enhancers**

Renata Bordeira-Carriço∗^,1^, Joana Teixeira^∗,2^, Marta Duque^2^, Diogo Ribeiro^1^, Mafalda Galhardo^3^, Rafael Dominguez-Acemel^4^, Firbas Panos^4^, Juan Tena^4^, Eufrasio Eufrasio^1^, Joana Marques^1^, Telmo Freitas^1^, Fatima Carneiro^5^, Jose Luis Goméz-Skarmet^4^, Jose Bessa^1^

1- Instituto de Investigação e Inovação em Saúde - i3S, Universidade do Porto, Porto, Portugal, Porto, Portugal; 2- Instituto de Investigação e Inovação em Saúde - i3S, Universidade do Porto, Porto, Portugal; Instituto de Biologia Molecular e Celular, Universidade do Porto, Porto, Portugal; Instituto de Ciências Biomédicas Abel Salazar, Universidade do Porto, Portugal; 3- Instituto de Ciências, Tecnologias e Agroambiente - CIBIO, Universidade do Porto, Portugal; 4- Centro Andaluz de Biología del Desarrollo, Universidad Pablo de Olavide, Spain; 5- Department of Pathology, Faculty of Medicine, University of Porto, Portugal; ∗equal contribution

The pancreas is a central organ for human diseases that have a dramatic societal burden, such as pancreatic cancer and diabetes. Non-coding cis-regulatory elements (CREs) of DNA control gene expression, being required for proper pancreas function. It has been shown that most disease- associated alleles are non-coding, many overlapping with CREs, suggesting that alterations in these regulatory sequences can contribute to human pancreatic diseases by impairing gene expression. However, functional testing of CREs in vivo is not fully explored. In this work we use Assay for Transposase-Accessible Chromatin (ATAC-seq), Chromatin Immunoprecipitation (ChIP-seq) and HiChIP-seq to identify CREs active in the adult zebrafish pancreas and their target genes. Using this data, we searched for human functional equivalent CREs, identifying disease-associated sequences across species. We found a zebrafish ptf1a distal developmental enhancer, which deletion generates pancreatic agenesis, as its human counterpart. Additionally, we identified a novel human pancreatic enhancer of the tumor suppressor gene *ARID1A*, which zebrafish deletion impairs gene transcription, conferring a potential tumor suppressor role to this non-coding sequence. This work explores the zebrafish pancreas regulome, identifying CREs important for pancreas development, function and homeostasis.

**Financial support:** ERC and FCT.

**P18-c-Src/Fyn role in Huntington's Disease progression**

Lígia Fão^1,3^, Sandra I. Mota^1,2^, A. Cristina Rego^1,2,3^

1- Center for Neuroscience and Cell Biology (CNC), University of Coimbra, Coimbra, Portugal; 2- Institute for Interdisciplinary Research (IIIUC), University of Coimbra, Coimbra, Portugal; 3- Faculty of Medicine, University of Coimbra, Coimbra, Portugal

Huntington's Disease (HD) is an autosomal dominant progressive neurodegenerative disorder affecting the striatum and later the cortex, with no effective neuroprotective therapies. Mutant huntingtin (mHTT), the main HD proteinaceous hallmark, participates in reactive oxygen species (ROS) formation, mitochondrial dysfunction and modified N-methyl-D-aspartate receptors (NMDAR) activity. Importantly, c-Src and Fyn, two ubiquitous members of the Src Kinase Family (SKF), are enriched in striatal neurons and implicated in brain neuronal development, transmission, synaptic regulation of NMDAR activity and mitochondrial function, and are activated by ROS. These observations favor a common inter-player between mHTT and HD-related neuronal dysfunction, suggesting a relevant role for c-Src/Fyn-regulated pathways in HD pathogenesis. In this study, we analyzed c-Src/Fyn levels in different HD models, namely in human postmortem caudate brain samples, brain tissue and primary neurons derived from YAC128 transgenic mice and STHdhQ111/ Q111, as well as the influence of autophagy on c-Src/Fyn regulation, using Western Blotting and immunocytochemistry. We also investigated the role of these kinases on NMDAR regulation in HD context, using calcium probes and immunocytochemistry. Our data showed consistent decreased c-Src/Fyn levels and activation in all models tested, when compared to the respective controls, along with augmented Fyn degradation by autophagy in HD. Moreover, primary striatal neurons from YAC128 mice evidenced decreased c-Src/Fyn levels in distal neurites and postsynaptic density, as well as diminished PSD-95 levels and puncta, suggesting altered synapse morphology and number in HD neurons. Concordantly, decreased c-Src/Fyn in YAC128 mice was accompanied by decreased Tyr1472 phosphorylation of GluN2B-composed NMDAR and by decreased NMDAR-mediated intracellular Ca^2+^ levels. We demonstrate c-Src/Fyn tyrosine kinases involvement in HD pathogenesis. Further studies should be performed to better understand the impact of Src/Fyn modulation on neuronal function in HD; however, this work supports that c-Src/Fyn-related pathways may constitute novel potential targets for HD.

**Financial support:** Supported by the European Regional Development Fund (ERDF), through the Centro 2020 Regional Operational Programme: project CENTRO-01-0145-FEDER-000012-HealthyAging2020.

**P19-Age-at-Onset variation in Val30Met Familial Amyloid Polyneuropathy: The genetic landscape of *TTR***

Miguel Alves-Ferreira^1,2^; Ana Azevedo^2^; Teresa Coelho^3^; Diana Santos^1,2^; Jorge Sequeiros^1,2^; Isabel Alonso^1,2^; Alda Sousa^1,2^; Carolina Lemos^1,2^

1- UnIGENe, IBMC - Instituto de Biologia Molecular e Celular; i3S – Instituto de Investigação e Inovação em Saúde, Universidade do Porto, Portugal; 2- ICBAS - Instituto Ciências Biomédicas Abel Salazar, Universidade do Porto, Portugal; 3- Unidade Corino de Andrade (UCA), Centro Hospitalar do Porto (CHP), Porto, Portugal.

Aims/Contex: Val30Met in transthyretin (*TTR*) gene is causative for familial amyloid polyneuropathy (FAP). Substantial phenotypic heterogeneity has been described in Val30Met patients, including in its age-at-onset (AO) between clusters, families, and among generations. Other variants at the *TTR locus*, outside the disease-causing variant, could play a regulatory role in its expression level and modify disease expressivity. We aim at identifying genetic variants at the *TTR locus* and interpret how they contribute to genetic modification in TTR-FAP. Methods: We analyzed DNA samples of 330 Val30Met carriers (299 patients, 31 aged-asymptomatic carriers) from 120 families currently under follow-up. A generalized estimating equation analysis (GEE) was used to take into account non-independency of AO between relatives. An intensive *in silico* analysis was performed in order to understand a possible regulation of gene expression. Results: We found 11 rare variants in the promoter, coding and intron/exon boundaries of the *TTR* gene associated with the onset of symptoms before and after age 40 years, namely 2 novel ones and a tandem CA-dinucleotide repeat. The seven Val30Met/Val30Met homozygous do not carry any of the variants identified in this study, including the common ones. *In silico* analysis disclosed significant alterations in the mechanism of splicing, transcription factors and miRNAs binding. Conclusion: Variants within the promoter region may modify disease expressivity and variants in the 3’UTR can impact the efficacy of novel therapeutic interventions. Importantly, the putative mechanisms of regulation of gene expression within the *TTR* gene deserve to be better explored, in order to be used in the future as potential therapeutical targets.

**Financial support:** FEDER – [COMPETE: POCI-01-0145-FEDER-007440]. FCT [PTDC/SAU-GMG/100240/2008 and PEsT]. FCT fellowship [SFRH/BD/101352/2014]. T.C.'s institution has received support from Pfizer.

**P20-Signal Transduction Pathways Regulating the Alternative Splicing of Tumor-Related RAC1b**

Vânia Gonçalves^1,2^, Paulo Matos^1,2^, Joana Pereira^1,2^, Andreia Henriques^1,2^ and Peter Jordan^1,2^

1- Departamento de Genética Humana, Instituto Nacional de Saúde Doutor Ricardo Jorge, Lisboa; 2- BioISI - Biosystems & Integrative Sciences Institute, Faculdade de Ciências da Universidade de Lisboa

Introduction: In colon cancer distinct genetic subtypes have been described, one of which involves overexpression of RAC1b, a variant generated by alternative splicing. Aberrant splicing is known to occur in cancer and can be caused by mutation in a gene or splicing factor but also represents a dynamic response to oncogene-induced cellular signaling and in this case it may be pharmacologically targeted. Here we explore how signaling pathways are involved in the deregulation of alternative RAC1b splicing in colorectal tumor cells. Materials and Methods: HT29 colorectal cells represent serrated colorectal tumors with *BRAF* gene mutation V600E in one allele and RAC1b overexpression. Cells were transfected with shRNA vectors directed against target candidate protein kinase transcripts and their effects on RAC1b levels analyzed 24 h later by Western Blot and qRTPCR. Treatment with kinase inhibitors or anti-inflammatory drugs was performed 24 h prior to cell lysis. Results and Discussion: Two kinases, SRPK1 and GSK3β, were found required to sustain RAC1b levels and both were shown to act upon the phosphorylation of splicing factor SRSF1, which binds to and promotes the inclusion of the alternative exon in RAC1b. SRPK1 knockdown or pharmacological inhibition reduced SRSF1 phosphorylation decreasing its nuclear translocation and concomitantly RAC1b splicing. The same regulatory pathway was also found to be controlled by GSK3β. Interestingly, GSK3β phosphorylation was identified to serve as target for the anti-inflammatory drug ibuprofen, decreasing SRPK1 nuclear translocation and inhibiting RAC1b overexpression. Together, our results demonstrate that oncogenic signal transduction pathways deregulate alternative splicing and this may be drug revertable.

**Financial support:** This work was supported by the Fundação para a Ciência e Tecnologia, Portugal and Association Maratona da Saúde.

**P21-The interplay between KIAA0753 and TBCCD1 in the control of ciliogenesis and cell cytoskeleton architecture**

David C. Ferreira^1,2^, Bruno Carmona^1,2,3^, Sofia Nolasco^3,4^, H. Susana Marinho^1,2^, Helena Soares^1,2,3^

1- Centro de Química Estrutural, Faculdade de Ciências da Universidade de Lisboa; 2- Centro de Química e Bioquímica, Faculdade de Ciências da Universidade de Lisboa; 3- Escola Superior de Tecnologia da Saúde de Lisboa, Instituto Politécnico de Lisboa; 4- Centro de Investigação Interdisciplinar em Sanidade Animal (CIISA), Faculdade de Medicina Veterinária, Universidade de Lisboa

Aims/Context: Cilia are slender protuberances found in eukaryotic cells, consisting of a microtubule (MT)-based ciliary axoneme, which can confer motility and sensory functions to the cells. These organelles have a basal body, which can be derived from the centrosome and nucleates the ciliary axoneme. Centriolar satellites are protein granules located around the centrosome that play an essential role in centrosome and primary cilium assembly. Mutations in genes encoding centrosome and/or centriolar satellite components lead to various human disorders, such as ciliopathies. Previous work from our group characterized the interactome of a new centrosomal TBCC domain-containing human protein (TBCCD1). Included among the identified proteins were several well-known proteins encoded by ciliopathy genes, e.g., centrosomal and centriolar satellites protein KIAA0753 (also known as OFIP and Moonraker). *KIAA0753* is mutated in several ciliopathic syndromes as, e.g., Joubert syndrome. Methods: Immunofluorescent microscopy and Western blot were used to study the levels and cellular localization of components of centriolar satellites, cytoskeleton and cilia, in human RPE1 cells overexpressing TBCCD1. Similarly, RPE1 cells depleted of either TBCCD1 or KIAA0753 were analyzed. Results: In RPE-1 cells, both the knockdown and overexpression of TBCCD1 affect the levels of KIAA0753, as well as its localization around the centrosome. This suggests that TBCCD1 plays a role in the recruitment of KIAA0753 to the centrosome. Regarding the levels of KIAA0753, we show that its depletion affects centriole connection with consequences on the organization of the MT network. This may compromise cell polarity, cell migration, and cilia assembly. Furthermore, we show that KIAA0753 localizes in the basal body and along the axoneme of cilia, which points to a role in ciliogenesis. Conclusions: Our results support a new function for KIAA0753 in centrosome structure maintenance, as well as a new functional interaction between TBCCD1 and KIAA0753, suggesting a new pathway in ciliopathy development.

**Financial support:** PEst-OE/QUI/UI0612/2013 and UID/MULTI/00612/2013 FCT; IPL/2019/MOONOFCILI/ESTeSL.

**P22-*APOE* allele frequency in late onset Alzheimer's disease patients from Iberia**

Ricardo González^1,2^, Sandra Martins^1,2^, Luis Durães^3^, Patrícia Sousa^3^, Manuel Figueruelo^4^, María Rodríguez^4^, Carmen Pita^4^, Miguel Arenas^5^, Luis Álvarez^6^, Roberto Hornero^7^, Carlos Gómez^7^, Nádia Pinto^1,2,8^, Alexandra M Lopes^1,2^, Iva Gomes^1,2^

1- Instituto de Patologia e Imunologia Molecular da Universidade do Porto (IPATIMUP), Portugal; 2- Instituto de Investigação e Inovação em Saúde (i3 s), Universidade do Porto, Portugal; 3- Associação Portuguesa de Familiares e Amigos de Doentes de Alzheimer, Portugal; 4- Asociación de Familiares y Amigos de Enfermos de Alzheimer y otras demencias de Zamora, Spain; 5- Department of Biochemistry, Genetics and Immunology, University of Vigo, Spain; 6- TellmeGen, Valencia, Spain; 7- Biomedical Engineering Group, University of Valladolid, Spain; 8- Centro de Matemática da Universidade do Porto, Portugal

Aims/Context: Alzheimer's Disease (AD) is a progressive neurodegenerative disease associated with cognitive decline. It is one of the most severe brain disorders affecting the elderly population, being secondary to the increase of life expectancy. Although multi-factorial, the primarily genetic risk factor for late-onset AD is the *Apolipoprotein E* (*APOE*) ε4 allele. The *APOE* gene encodes a 299 amino acid protein that plays a key role in the transport and metabolism of plasma cholesterol and triglycerides, as well as in injury repair in the brain. *APOE* isoforms differ in the amino acids 112 and 158, which affect its structure, influencing its capability to bind to lipids and receptors, leading to the onset of AD. Due to the preponderant role in AD pathology, screening the *APOE* gene can facilitate AD diagnostics. Methods: DNA was extracted from saliva samples from 95 patients with clinical diagnostic of AD from Iberia (Northern Portugal and Castile and León). After whole genome amplification, we sequenced the *APOE* locus by Sanger sequencing to analyze SNPs rs429358 and rs7412, therefore assessing *APOE* alleles in our cohort of AD patients.

Results: We observed 34% of individuals carrying the risk *APOE* ε4 allele, whereas the more common ε3 allele was present in 59% of the patients. The frequency of ε2 allele (associated to a decreased disease risk) was estimated in our sample set with a frequency of 7%. Conclusions: This work is a preliminary study about the frequency distribution of the *APOE* polymorphic ε2, ε3 and ε4 alleles in a cohort of late-onset patients of AD from Northern Iberian regions. The genetic characterization of *APOE* provides a forecast on the landscape of AD risk in these regions based on the haplotype data obtained from *APOE* alleles at SNPs rs429358 and rs7412.

**Financial support:** Project 1317_AD-EEGWA, INTERREG, POCTEP (2014-2020). FCT (POCI-01-0145-FEDER-007274). FCT: CEECIND/00684/2017, IF/01262/2014, CEECIND/02609/2017, SFRH/BPD/97414/2013. Spain: RYC-2015-18241.

**P23-Sperm DNA damage, active mitochondria and spermatic parameters: influence of the lifestyle, body mass index and age.**

Patrícia Pinho^1^, Regina Arantes-Rodrigues^1,2^, Zélia Gomes^3^, Miguel Brito^3^, Osvaldo Moutinho^3^, Isabel Gaivão^4^, Bruno Colaço^2^, Rosário Pinto-Leite^1^

1- Genetics/Andrology Laboratory, Hospital Centre of Trás-os-Montes and Alto Douro, Vila Real, Portugal; 2- Centre for the Research and Technology of Agro-environmental and Biological Sciences, University of Trás-os-Montes and Alto Douro, Vila Real, Portugal; 3- Hospital Centre of Trás-os-Montes and Alto Douro, Department of Obstetrics and Gynecology, Vila Real, Portugal; 4- Animal and Veterinary Research Centre, University of Trás-os-Montes and Alto Douro, Vila Real, Portugal

Sperm DNA damage and an altered mitochondrial activity have been related to male infertility. Several common lifestyle (LS) factors, obesity and advanced paternal age may affect sperm quality, although the consequences are not unanimous in the literature. The aim of this study was to evaluate the impact of LS factors (smoking, alcohol intake and exposure to harmful occupational factors), body mass index (BMI) and men's age on sperm (spz) DNA damage, active mitochondria (AM) and spermatic parameters (SP). A total of 149 men (22–52 years old) undergoing infertility investigation collected a sample for routine semen analysis and were asked to complete an anonymous questionnaire about their LS. In 26 individuals, it was evaluated the sperm DNA integrity by Alkaline Comet and TUNEL assays, and the percentage of spz with AM (MitoTrackerTM Red FM dye). Based on the absence or presence of one/more risk factors associated with the LS, two groups of men were formed: with risks (R) and without risks (NR) associated. DNA integrity, spz with AM and SP were compared between individuals R and NR. The SP were also compared between normal weight (NW), overweight (OW) and obese (O) men. The same comparison was made exclusively in individuals NR. We also evaluated the presence of correlations (r) between BMI and DNA damage and spz with AM in individuals R and NR; and between men's age and DNA damage, spz with AM and SP. Individuals NR tended (p>0.05) to have sperm samples with less DNA damage (69.8AU vs 73.2AU), less spz with fragmented DNA (4.6% vs 5.3%), more spz with AM (70.3% vs 66.5%) and better SP than the individuals R. In individuals R the higher the BMI, the more DNA damage the sperm samples presented (r = 0.717, p = 0.030), although in individuals NR it was only observed a trend (r = 0.661, p = 0.053). Individuals O presented worse SP than individuals NW or OW (p>0.05), with statistical significance only in individuals NR. Regardless of the LS, the older the men, the more DNA damage the semen samples presented (r = 0.523, p < 0.01). Given the results, it is urgent to sensitize the population to adopt a healthy lifestyle and to warn about the decline in semen quality with age.

**Financial support:** This study was funded by the Hospital Centre of Trás-os-Montes and Alto Douro and by the projects UID/CVT/00772/2019 and UID/AGR/04033/2019, supported by FCT.

**P24-Zebrafish: an interesting model to study CDKL5 deficiency disorder**

Tatiana Varela^1^, Gil Martins^1^, Débora Varela^1,3^, Marta Vitorino^2,3^, Natércia Conceição^1,2,3^, M. Leonor Cancela^1,2,3^

1- Centre of Marine Sciences (CCMAR), University of Algarve, Faro, Portugal; 2- Department of Biomedical Sciences and Medicine and Algarve Biomedical Centre, University of Algarve, Faro, Portugal; 3- Centre for Biomedical Research (CBMR), University of Algarve, Faro, Portugal

CDKL5 deficiency disorder is a rare X-linked condition that results in early onset of impaired motor and cognitive skills such as motor rigidity, stereotypical hand movements and deficient language acquisition as well as recurrent seizures. It is caused by mutations in the cyclin-dependent kinase-like 5 (*CDKL5*) gene, which encodes a serine/threonine kinase involved in important neuronal processes such as cell signaling and neuron morphogenesis. CDKL5 is responsible for its autophosphorylation as well as the phosphorylation of its substrates including AMPH, MECP2, MAP1S and ARHGEF2. Although CDKL5 deficiency is a very severe condition, the mechanisms involved in its onset are not clearly understood and existing mouse models do not fully mimic the human phenotype. Thus, the use of alternative models represents a powerful tool to further study CDKL5 deficiency disorder. Zebrafish has been shown to be a suitable biomedical model and shares many physiological processes with human; therefore our objective was to validate the use of zebrafish as a model to study CDKL5 deficiency disorder. Through a comparative *in silico* analysis we confirmed that gene structure of zebrafish *cdkl5* and Cdkl5 substrates appear to be conserved when compared to their human orthologs. The corresponding proteins also show a degree of sequence conservation, particularly Cdkl5 catalytic domains required for phosphorylation. Zebrafish larvae were exposed to PTZ, a seizure inducing drug, total RNA was extracted and qPCR was carried out to investigate expression levels of neuronal marker genes. The results show a downregulation of *mecp2* and an upregulation of *bdnf* after PTZ treatment. Immunohistochemistry of zebrafish brain sections following treatment with PTZ showed a clearly alteration of cdkl5 expression, mostly in telencephalon, comparing with the control. We are presently conducting experiments in zebrafish with morpholinos to suppress Cdkl5 expression in order to investigate the resulting morphological, behavior and molecular changes. In conclusion, our results contribute to validate the use of zebrafish as a suitable model for the study of CDKL5 deficiency disorder mechanisms.

**Financial support:** Million Dollar Bike Ride Grant program from Orphan Disease Center, University of Pennsylvania, USA, Award Number: MDBR-19-104-CDKL5 and national funds from FCT through UID/Multi/04326/2019 (CCMAR)

**P25-A BRCA2 intronic variant of uncertain significance alters pre-mRNA splicing**

Célia Carvalho^1^, Noélia Custódio^1^, Patrícia Dias^2^, Beatriz Porto^3^, Ana Berta Sousa^2^, Maria Carmo-Fonseca^1^

1- Instituto de Medicina Molecular João Lobo Antunes, Faculdade de Medicina, Universidade de Lisboa, Lisboa, Portugal; 2- Serviço de Genética, Departamento de Pediatria, Hospital de Santa Maria, Centro Hospitalar Lisboa Norte, Centro Académico de Medicina de Lisboa, Lisboa, Portugal; 3- Laboratório de Citogenética, Instituto de Ciências Biomédicas Abel Salazar, Universidade do Porto, Porto, Portugal

Context/Aim: Heterozygous germline mutations in the BRCA2 gene are associated with an increased risk for developing breast and ovarian cancers, whereas biallelic pathogenic germline variants in BRCA2 cause Fanconi Anemia. However, a large fraction of genetic alterations found in the BRCA2 gene are classified as variants of uncertain significance (VOUS), precluding an appropriate clinical approach to patients and relatives. The aim of this study was to characterize the splicing pattern and stability of BRCA2 mRNAs in cells from two sisters with cancer that carry a biallelic intronic variant in the BRCA2 gene classified as VOUS. Analysis of DNA crosslink sensitivity was negative for Fanconi Anemia but suggested the presence of genomic instability. Methodology and results: The variant studied localizes in the intron downstream of exon 8, BRCA2: c.681+5G>C. Using a high precision quantitative RT-PCR assay, based on the digital droplet PCR technique (ddPCR), we show that the great majority of BRCA2 mRNAs produced in the patient cells exclude exon 8. The exclusion of exon 8 results in frameshift and generation of a premature stop codon (PTC) that is expected to drive mRNA to Nonsense Mediated Decay (NMD). We developed an assay based on ddPCR to confirm that BRCA2 transcripts in the patient’ PBMCs (peripheral blood mononucleated cells) were indeed targets of NMD. We further found a newly identified exclusion of exon 7 in cells from control donors as well as in the cells from the patients harboring the variant. Conjugated skipping of exon 8 and exon 7 abrogates the frameshift, leading to the expression of an internally truncated BRCA2 protein. Conclusion: Our results suggest that in normal cells the exclusion of exon 7 commits the mRNA to degradation by NMD, whereas in the patients’ cells it prevents degradation of the mis spliced mRNA resulting in expression of an abnormal BRCA2 protein. We are currently studying DNA repair function in the patient cells.

**Financial support:** Fundação para a Ciência e Tecnologia e Programa Operacional Regional de Lisboa (LISBOA-01-0145-FEDER-029469).

**Clinical Research**

**P27-2q31.1 microdeletion syndrome: mapping the clinical phenotype**

Raquel Rodrigues^1^, Rosário Santos^1^, Sónia Custódio^1^, Juliette Dupont^1^, Oana Moldovan^1^, André Travessa^1^, Marta Soares^1^, António Macedo^2^, Ana Sousa^1^, Ana Berta Sousa^1^

1- Serviço de Genética Médica, Departamento de Pediatria, Hospital de Santa Maria, CHULN, CAML, Lisboa, Portugal; 2- Serviço de Pediatria, Hospital São Francisco Xavier, CHLO, Lisboa, Portugal

Context: Microdeletions of 2q31.1 region are rare. The clinical phenotype is variable, including intellectual disability (ID), facial dysmorphism and limb defects of varying severity. Less frequently the brain, eyes, heart, and urogenital system may also be affected. Haploinsufficiency of the HOXD gene cluster has been linked to limb anomalies, however the etiology of ID remains unclear. We describe three new cases with 2q31.1 microdeletion, aiming to contribute to the genomic mapping of clinical features for this rare syndrome. Methods: Case 1- 9yo boy, mild ID. Bitemporal narrowing, small palpebral fissures, strabismus, prominent columella, thin upper lip, retrognathia, cupped ears with thickened helices and lobes; 3^rd^ finger camptodactyly, 5^th^ finger clinodactyly; 2^nd^/3^rd^ toes complete syndactyly; cryptorchidism. Case 2- 6yo girl, mild ID and ADHD. Small palpebral fissures, retrognathia, dysplastic helices; broad hallux, sandal gap and 2^nd^/3^rd^ toe syndactyly.

Case 3- 9yo girl, mild ID. Microcephaly; triangular face, narrow forehead, underfolded helices, misaligned teeth, high palate; 5^th^ finger clinodactily; 2^nd^/3^rd^ toes partial sindactily. DNA samples were studied by aCGH (180K CGX-HD). Available parents were studied by FISH analysis. Results/discussion: 2q31.1 microdeletion was identified in all cases: 1-(173550859_176967147)x1; 2-(173120478_176272245)x1 dn; 3-(174436582_175704751)x1. Deletions of HOXD genes result in hand/foot anomalies. Although all our patients have limb defects, only case 1 encompasses HOXD genes, reinforcing the hypothesis that deletion of upstream regulatory elements may also cause limb anomalies. *DLX1/2, RAPGEF4* and *CHN1*, all playing a role in brain development, were suggested as candidates for ID etiology. Only *CHN1* is included in the minimal overlap deleted region in our cases, supporting its key contribution to ID in these patients. *CHN1* participates in the pruning of dendritic arbors. Disruption of these events during circuit maturation and refinement may lead to brain dysfunction and neurological disease. Our cases, with breakpoints defined by aCGH, contribute to the genomic mapping of clinical features for this rare syndrome.

**P28-Reproductive Options and Familial Amyloid Polyneuropathy**

Teresa Saraiva^1^, Patrícia Rodrigues^2^, Ana Fortuna^1,3^

1- Medical Genetics Department, Centro de Genética Médica Doutor Jacinto Magalhães, Centro Hospitalar Universitário do Porto, EPE, Porto, Portugal; 2- Cardiology Department, Centro Hospitalar Universitário do Porto, EPE, Porto, Portugal; 3- Unit for Multidisciplinary Research in Biomedicine, ICBAS-UP Porto, Portugal.

Introduction Familial Amyloid Polyneuropathy (FAP), Portuguese type, is a late onset neurodegenerative disease with high penetrance and impressive morbidity. Prenatal diagnosis (PND) and preimplantation genetic diagnosis (PIGD) are currently available as reproductive options (RO), the later since 2001. Methods Between January 2018 and July 2019, a representative cohort of FAP subjects followed at FAP cardiology consultation of our hospital, aged between 18 and 55 years, were requested to complete an anonymous questionnaire about their RO. The aim of this study is to determine the current knowledge about RO, analyze their choices, information sources and the impact of genetic counseling on their decisions.

Results A total of 126 subjects volunteered to answer the questionnaire: 40% were females; 66% had less than 12 years of schooling education; 62% were married and 53% claimed to have RO information. Thirty-nine percent had medical genetics consultation (MGC), being the FAP neurology consultation the main information source (42%), followed by MGC (18%). In general, the prevalence of PND and PIGD use was 6% and 13% with a successful pregnancy of 63% and 38%, respectively. Nine percent chose not to have children due to fear of transmission. In MGC group (49/126), the prevalence of PND and PIGD use was 6% and 14%, 22% chose not undergo reproductive planning and 12% were unaware of RO. In those without MGC (77/126), the prevalence of PND and PIGD use was 7% and 10%, 27% chose not undergo reproductive planning and 29% were unaware of RO. In those with offspring born after PIGD availability (44/126), 41% have had MGC, 68% did not use reproductive techniques, the main reasons being choice and RO unawareness. In those that planned to have children (25/126), 52% had MGC, 92% elected PIGD for reproductive family planning since most (88%) didn’t want to terminate a pregnancy; 17% reported the waiting time as the reason for not choosing PIGD. Conclusions This study highlights the importance of medical genetics in the multidisciplinary approach of this disease. A greater investment should be made in reproductive counseling and in facilitating access to RO.

**P31- Liquid Biopsy in CRC patients – when no tumor sample is available**

Ana Oliveira^1^, Luís Nogueira^2^, Carolina Ribeiro^2^, Rui Oliveira^3^, Margarida Abrantes^4,5^, José Guilherme Tralhão^1,4^, Henriqueta Coimbra Silva^4^

1- Surgical Department, Hospitais da Universidade de Coimbra, Centro Hospitalar e Universitário de Coimbra; 2- Institute of Medical Genetics/UCGenomics, Faculty of Medicine, University of Coimbra; 3- Pathology Department, Hospitais da Universidade de Coimbra, Centro Hospitalar e Universitário de Coimbra; 4- Faculty of Medicine, Coimbra Institute for Clinical and Biomedical Research (iCBR), University of Coimbra, Coimbra, Portugal; 5- Biophysics Institute, IBILI, Faculty of Medicine, University of Coimbra;

Introduction: Liquid biopsy (LB) has been emerging as a useful tool in the management of colorectal cancer (CRC) patients, both at diagnosis and follow-up. Its major advantages include being a minimally invasive methodology and the possibility of better assessing the molecular heterogeneity and dynamics of the disease. Currently, expanded tumor RAS analysis is mandatory for patients with metastatic CRC being considered for EGFR-targeted monoclonal antibodies. For patients with no molecular analysis of primary tumor, LB may be the best choice. Methods: Six CRC patients’, stage IIA to IV were studied. Tumor molecular results were only available for two patients. Peripheral blood samples (5–7 ml) in EDTA tubes were processed for plasma collection within one hour. Cell-free DNA (cfDNA) was extracted from 2.5 -3.5 ml of plasma using MagMax cfDNA isolation Kit. Molecular profile was performed with Oncomine Colon cfDNA Assay. Ion Chef System and Ion 530 Kit-Chef were used for template preparation. A 50pM library pool containing six samples were applied in Ion 530 Chip and sequenced on the Ion Gene Studio S5 Plus platform (Thermo Fisher Scientific). For variant analysis, Torrent Variant Caller plugin, Torrent Suite Software v5.2.1 and the Ion Reporter online tool were used (Thermo Fisher Scientific). Results: Plasma cfDNA concentration varied from 0.6 to 15.7 ng/μl, the higher value being detected in a patient with multiple metastasis. Using a cfDNA input ranging between 8 to 31 ng, limits of detection (LoD) from 1.25 to 0.1% were achieved. KRAS actionable mutations were detected in five patients including the two patients with tumor molecular study (concordant results). Five patients had driver mutations in three or more genes, the most frequently mutated being KRAS, PIK3CA and APC. Driver mutations in seven genes were identified in the LB of a patient with evidence of mismatch repair (MMR) system deficiency in tumor biopsy. Conclusion: in patients with high stage CRC, with no primary biopsy of tumor tissue available, LB using NGS approach may evaluate KRAS mutational status and identify other eventually actionable or prognosis-associated somatic variants.

**Financial support:** Funding from UCGenomics is a member of GenomePT Consortium (POCI-01-0145-FEDER-022184)

**P32- Diagnosis of congenital fibrinogen deficiencies – a center**'**s experience**

Catarina S. Pinto^1^, Patrícia Martinho^1^, Inês C. Conceição^2^, M. Fátima Rodrigues^3^, Cristina Oliveira^3^, Ramón Salvado^4^, Marina Costa^5^, Teresa Fidalgo^1^ & M. Letícia Ribeiro^1^

1– Laboratório Hematologia Molecular, Serviço de Hematologia Clínica, Centro Hospitalar e Universitário de Coimbra; 2– Serviço de Patologia Clínica, Centro Hospitalar Barreiro Montijo, Barreiro; 3– Serviço de Imunohemoterapia, Hospital Santa Maria, Lisboa; 4– Serviço de Sangue e Medicina Transfusional, Centro Hospitalar e Universitário de Coimbra; 5– Serviço de Imunohemoterapia, Hospital de São Teotónio, Viseu

Aim/Context: Congenital fibrinogen deficiencies are rare disorders, classified as quantitative (hypofibrinogenemia/afibrinogenemia, with partial/complete absence of fibrinogen) or qualitative (dysfibrinogenemia and hypodysfibrinogenemia, with normal or reduced fibrinogen levels and/or abnormal functional activity). Clinically, quantitative deficiencies are associated with hemorrhagic events, while dysfibrinogenemias are mainly asymptomatic, or can present with bleeding, thrombosis or both. Fibrinogen is a hexameric protein consisting of three pairs of polypeptide chains (Aa, Bb and g), encoded by FGA, FGB and FGG genes, respectively. Variants in these genes have been reported, both in homo- or heterozygosity. Our aim was to identify the molecular diagnosis of patients with fibrinogen anomalies and/or hemorrhagic diathesis of unknown cause by next generation sequencing (NGS). Methods: We have analyzed 17 unrelated patients and 5 relatives. The molecular diagnosis was done using a custom panel for NGS (43 genes). Library preparation and sequencing was done using IonS5 (TFS) protocol. Results: We have identified 13 different variants (pathogenic and potentially pathogenic) in FGA (5), FGB (2) and FGG (6) genes, including six new variants in FGB (Glu240Lys and Arg196Cys) and FGG (Asp63Val, Trp360∗, Glu422del and Tyr27Cys). The patients were diagnosed as hypofibrinogenemia (n = 7), hypodisfibrinogenemia (n = 4) and dysfibrinogenemia (n = 4). The patients diagnosed with afibrinogenemia (n = 2) were homozygous for 2 variants in FGA (complete deletion and Arg181∗) and had severe hemorrhagic manifestations. Five presented with thrombosis: one was carrier for FGA Cys64Tyr, associated with both thrombosis and bleeding. In total, 11 patients were asymptomatic. Conclusions: We have identified 6 new variants in the coding region of the fibrinogen cluster, together with high phenotypic variability. A phenotype-genotype correlation was observed in the quantitative deficiencies. In dysfibrinogenemias, it is still a challenge. We add another example of the valuable tool that NGS represents to clinical practice, allowing for a faster and broader diagnosis of fibrinogen hereditary anomalies.

**P33- Circulating Cell-free DNA levels as a potential biomarker in cancer – a preliminary study**

Inês Tavares^1^, Luís M Pires^1^, Ilda P Ribeiro^1,2^, Leonor Barroso^3^, Ivana Martins^1^, Alexandra C. Oliveira^1^, Joana B. Melo^1,2,4^, Isabel M Carreira^1,2,4^

1- Cytogenetics and Genomics Laboratory, Faculty of Medicine, University of Coimbra, Coimbra, Portugal; 2- CIMAGO - Center of Investigation on Environment Genetics and Oncobiology - Faculty of Medicine, University of Coimbra, Coimbra, Portugal; 3- Maxillofacial Surgery Department, Coimbra Hospital and University Centre, CHUC, EPE, Coimbra, Portugal; 4- iCBR - Coimbra Institute for Clinical and Biomedical Research, Coimbra, Portugal

Context: Cell-free DNA (cfDNA) is extracellular DNA present in plasma of both healthy individuals and patients with benign lesions, inflammatory diseases, tissue trauma and cancer. The release of DNA into blood might rise to higher levels in cancer patients as a consequence of the necrotic and apoptotic processes typical of tumor cells. These increased cfDNA levels in cancer patients suggest a clinical relevance for the diagnosis and prognosis of those patients, as well as for disease monitoring. The aim of this preliminary study was to evaluate the ability to isolate cfDNA from plasma and then compare cfDNA levels of different patients with head and neck cancer in different stages of the disease, before and after treatment. Methods: cfDNA was isolated from 2 mL of plasma from 12 samples using a kit (QIAamp Circulating Nucleic Acid kit; Qiagen Manchester Ltd, Manchester, UK) adapted from the manufacturer's instructions. Results: Four of the patients (stages I to IV) were only analyzed before the treatment and four other patients were analyzed also after the treatment (surgery or chemotherapy/radiotherapy). When evaluating pre-treatment samples, no large variations in cfDNA were observed. Comparing pre and after treatment cfDNA levels we observed an increase after treatment. Tumor staging and BMI of the patients were correlated with these results. Conclusions: This preliminary study allowed us to see the differences in the cfDNA levels in cancer patients and evaluate the clinical relevance of those levels for the diagnosis, prognosis and follow-up of patients.

**P34- MLPA and array CGH evaluation in Oral Squamous Cell Carcinoma**

Ana Santos^1^, Ilda P Ribeiro^1,2^, Luísa Esteves^1^, Francisco Marques^3^, Leonor Barroso^4^, Maria J Julião^5^, Isabel P Baptista^3^, Isabel M Carreira^1,2^ and Joana B Melo^1,2^

1- Cytogenetics and Genomics Laboratory, Faculty of Medicine, University of Coimbra, Coimbra, Portugal; 2- iCBR-CIMAGO - Center of Investigation on Environment Genetics and Oncobiology - Faculty of Medicine, University of Coimbra, Coimbra, Portugal; 3- Department of Dentistry, Faculty of Medicine, University of Coimbra, Coimbra, Portugal; 4- Maxillofacial Surgery Department, Coimbra Hospital and University Centre, CHUC, EPE, Coimbra, Portugal; 5- Department of Pathology, Coimbra Hospital and University Centre, CHUC, EPE, Coimbra, Portugal

Introduction: Head and Neck cancer affects seven anatomical locations; amongst them, in the oral cavity, squamous cell carcinoma is the most common (90%) histological type. Oral squamous cell carcinoma (OSCC) affects different oral regions, most frequently the floor of the mouth and the tongue. Due to their proximity with structures with vital importance and with lymph nodes, this cancer still has a high mortality rate. As for genetic imbalances, alterations in copy number occurring in gene-dosage sensitive genes can have an important carcinogenic role in this disease, since associations between some imbalances and OSCC, such as in *TP53*, *RB1*, *EGFR* and *FHIT*, have already been reported. Aims: Identification of genetic imbalances that can be relevant as biomarkers, by characterizing copy number alterations (CNAs) in a cohort of squamous cell carcinomas, from the tongue and the floor of the mouth. Methodologies: Two different techniques, Multiplex Ligation-dependent Probe Amplification (MLPA) and microarray-based Comparative Genome Hybridization (aCGH) were performed in a 62 samples cohort. For MLPA a specific probe panel was used to evaluate CNAs. Data analysis was performed by Excel and by R (version 3.5.2). Results: The type of genetic alterations with higher frequency in our cohort were gains. MLPA most common imbalances were seen in chromosomes 3, 8 and 11, particularly in the 11q13.3 region, which presented high number of gains in *FADD*, *FGF4*, *CCND1* and *CTTN* genes. In aCGH, the most frequent abnormalities were seen in 8p23.1, 8p11.22, 6p25.3, 15q11.2, 5q13.2 and 11q11 regions. Common alterations to both locations detected in high frequency were observed in 8p23.1, 8p11.22, 8q24.3 and 3q29 regions. Conclusion: We were able to find that 11q13.3 is a hotspot region in this cancer, showing high rates of gains by both techniques. MLPA and aCGH also showed that chromosomes 3, 8 and 11 were the most commonly altered. Correlation between the detected genetic imbalances and clinicopathological features is crucial to propose novel biomarkers with prognostic value.

**P36- From copy number alterations to genomic correlations in oral cavity cancer – Cohort of patients from Brazil**

Inês Saraiva^1^, Luísa Esteves^1^, Inês Tavares^1^, Marília Santiago^2^, Cármem Bertuzzo^2^, Joana Barbosa Melo^1,3^, Isabel M Carreira^1,3^, Ilda P Ribeiro^1,3^

1- Cytogenetics and Genomics Laboratory, Faculty of Medicine, University of Coimbra, 3000–354 Coimbra, Portugal; 2- Laboratório de Genética Molecular, Departamento de Genética Médica – Faculdade de Ciências Médicas – Unicamp, Brazil; 3- CIMAGO - Center of Investigation on Environment Genetics and Oncobiology - Faculty of Medicine, University of Coimbra, 3000–354 Coimbra, Portugal

Introduction: Oral squamous cell carcinoma (OSCC) presents a high incidence and mortality worldwide. The progress of whole-genome technologies has opened new opportunities to explore cancer-associated biomarkers with diagnostic and therapeutic applications. This study aimed to perform a genome-wide characterization of OSCC patients and to identify the most common altered chromosomes and genes related to cancer development. Methods: The genomic characterization of 22 tumor tissue samples with oral cavity cancer diagnosis from a cohort of Brazil was performed using array comparative genomic hybridization technique. A fraction of alteration (number of altered base pairs over total base pairs) was calculated for every chromosomic arm, which was then used to determine the Spearman correlation coefficient, in order to find concomitant alterations. Results: We detected imbalances in almost all chromosomes; however, it was possible to verify that the most common losses and gains were observed in specific chromosomal regions. The most frequent gains were observed at 14q32, 11q21, 3q29, 17q12, 4q34, 3q24, 7q11, 8q11, 4q13, 8q11, 1p36, 11p15, 11q24, 19q13, 8q24, 20q11, 22q11, 7q21, 9q22, 15q11, 3q25, 3q28, 15q15, 20p12, 4p16, 11q11, 11q12, 11q14, 11q23 and 3q26, and the most frequent losses were observed at 8p23, 10q11, 6p25, 8p11, 11p15, 12p13, 6p21, 4q13, 11q11, 3p26, 5p15, 9p24, 5q11, 15q11. Several genes with a possible association with OSCC diagnosis and prognosis were highlighted, namely *ADAM3A* (8p11), *CDKN1C* (11p15), *MAML2* (11q21), *ASIC2* and *CCL2* (17q12). Regarding the Spearman correlation coefficient, we observed a strong correlation between 3p and 8q, i.e. in our cohort we simultaneously verify 3p loss and 8q gain. Additionally, a strong correlation between gains in 5p and 7p and in 5p and 12p was also observed. Regarding copy number losses, a strong correlation was observed between 3p and 9p. Conclusions: These results are in agreement with other cohort of patients studied in our lab, showing the importance of genomic evaluation to identify possible diagnostic and prognostic biomarkers. The correlation between molecular and clinic-pathological data is vital.

**P37-Genomic characterization of chronic lymphocytic leukaemia and multiple myeloma patients: aCGH contribution**

Alexandra C. Oliveira^1^, Diana A. Adão^1^, Luís M. Pires^1^, Ilda P Ribeiro^1,2^, Ana C. Gonçalves^2,3^, Artur Paiva^2,4^, Catarina Geraldes^2,5^, Adriana Roque^5^, Telma Nascimento^5^, Sara Duarte^5^, José Pedro Carda^2,3,5^, Amélia Pereira^2,6^, Joana Azevedo^5^, Maria Letícia Ribeiro^2,5^, Joana B. Melo^1,2^, Ana B. Sarmento-Ribeiro^2,3,5^, Isabel M Carreira^1,2^

1- Cytogenetics and Genomics Laboratory, Faculty of Medicine, University of Coimbra, Coimbra, Portugal; 2- iCBR-CIMAGO - Centre of Investigation on Environment Genetics and Oncobiology - Faculty of Medicine, University of Coimbra, Coimbra, Portugal; 3- Laboratory of Oncobiology and Haematology and University Clinic of Haematology, Faculty of Medicine, University of Coimbra, Coimbra, Portugal; 4- Cytometry Operational Management Unit, Clinical Pathology Service of Centro Hospitalar e Universitário de Coimbra (CHUC), Coimbra, Portugal; 5- Clinical Haematology Department of CHUC, Coimbra, Portugal; 6- Medicine Department, Hospital Distrital da Figueira da Foz, Figueira da Foz, Portugal.

Context: The characteristic variable clinical behaviour of haematological malignancies reflects the tumour biological heterogeneity and ultimately, the tumour genomic abnormalities. In chronic lymphocytic leukaemia (CLL) and multiple myeloma (MM) the heterogeneous genomic landscape has long been reported. Conventional Cytogenetics (CC) and Interphase Fluorescence in Situ Hybridization (i-FISH) became common clinical practice in the detection of cytogenetic alterations. However, both techniques have limitations: CC implies division cells while i-FISH is a targeted technique. Array Comparative Genomic Hybridization (aCGH) detects copy number variations at a genome-wide level, allowing clinically relevant abnormalities detection, otherwise missed. Therefore, the goal of this work was to characterize the genome of CLL and MM patients, by aCGH and to compare the suitability of aCGH and i-FISH (results from the clinical practice) in the detection of genomic abnormalities. Methods: A total of 19 CLL patients, 1 monoclonal B lymphocytosis, 15 MM and 1 monoclonal gammopathy of undetermined significance were analysed by aCGH. Mononuclear cells of CLL patients and plasma cells from MM patients were respectively isolated from peripheral blood and bone marrow samples, followed by genomic DNA extraction.

Results: In patients clinical routine, i-FISH studied a few number of regions previously associated to some prognostic value. For its part, aCGH allowed a whole genome research and the identification of new disease-associated chromosomal regions. The most frequent genetic alterations found in CLL patients’ samples were 14q and 13q deletions (40% and 35%) and trisomy 12 (30%), whereas in MM patients’ samples the most common genomic abnormalities were 13q deletions (63%), 1q gains (44%) and trisomies 9 and 7 (38%), already described in literature.

Conclusions: Whole genome level characterization of tumour samples was performed by aCGH, detecting alterations in regions other than the ones evaluated by standard method i-FISH. Nevertheless, i-FISH detects low expression mosaicisms and balanced rearrangements, denoting the convenience of applying both techniques at the clinical level.

**Financial support:** Projeto financiado pela Bolsa de Investigação em Oncologia do Núcleo Regional do Centro da Liga Portuguesa Contra o Cancro, em parceria com o Centro de Investigação em meio Ambiente, Genética e Oncobiologia.

**P38- Intratumor genetic and epigenetic heterogeneity in oral cancer**

Ilda P Ribeiro^1,2^, Inês Tavares^1^, Francisco Marques^3^, Leonor Barroso^4^, Joana B Melo^1,2^, Isabel M Carreira^1,2^

1- Cytogenetics and Genomics Laboratory, Faculty of Medicine, University of Coimbra, Coimbra, Portugal; 2- iCBR_CIMAGO—Center of Investigation on Environment Genetics and Oncobiology—Faculty of Medicine University of Coimbra, Coimbra, Portugal; 3- Department of Dentistry, Faculty of Medicine, University of Coimbra, Coimbra, Portugal; 4- Maxillofacial Surgery Department, Coimbra Hospital and University Centre, CHUC, EPE, Coimbra, Portugal.

Background: Cancer presents intertumoral and intratumoral heterogeneity, since tumors comprise subpopulations of different cells either within a primary tumor or between tumors of different tissues. The diagnostic and management of cancer patients is difficult due to the presence of tumor heterogeneity, which could be originated by subpopulations of distinct cells with nonrecurring mutations and genomic alterations as well as by clonal evolution and positive selective pressure from therapeutics. The molecular heterogeneity of oral cancer seems to hamper the development and efficacy of target treatments. This study aimed to perform a genetic and methylation characterization of oral tumor samples and to evaluate the presence of intratumor heterogeneity. Methods: Tumor and non-tumor tissue samples from 9 patients diagnosed with oral cavity squamous cell carcinoma were analyzed using Methylation Specific Multiplex Ligation-dependent Probe Amplification (MS-MLPA). From each patient 4 different biopsy tissue sites -two from distinct regions of tumor and two from distinct regions of non-tumor -were analyzed. Results: From the 9 patients, a total of 36 samples were analyzed (tumor and non-tumor), being *WT1* gene methylated in 18 samples. The second most common methylated gene was *GATA5* in 8 samples. We identify few copy number alterations, being the most frequent gain observed at *GSTP1* gene and the most frequent loss observed at *CDKN2A* gene. We found intratumor heterogeneity in different genes of 6 patients from the 9 analyzed. Conclusion: Our results highlight the challenges and difficulties to identify a comprehensive molecular profile of oral carcinoma with single site biopsy, which could have implications for precision medicine and clinical patient's management and follow-up.

**P39-Molecular diagnosis of haemophilia A: four novel variants identified in five patients**

Rita Certã^1^, Isabel Moreira^1^, Ema Antunes^2^, Eugénia Cruz^3^, Maria João Diniz^2^, Paula Kjollerstrom^4^, Sara Morais^3^, João Gonçalves^1^

1- Unidade de Genética Molecular, Departamento de Genética Humana, Instituto Nacional de Saúde Doutor Ricardo Jorge, Lisboa; 2- Serviço de Imunohemoterapia, Hospital de S. José, Centro Hospitalar Lisboa Central, Lisboa; 3- Serviço de Hematologia Clínica, Hospital Geral de Santo António, Centro Hospitalar do Porto, Porto, Portugal; 4- Unidade de Hematologia, Hospital D. Estefânia, Centro Hospitalar Lisboa Central, Lisboa, Portugal.

Aims/Context: Haemophilia A (HMA) is an X-linked bleeding disorder caused by reduced levels of the coagulation factor VIII (FVIII) due to alterations in the *F8* gene. Decreased levels of FVIII activity leads to a loss of clotting activity and to bleeding (predominantly into joins, muscles and inner organs). The severity of HMA ranges from mild (5–30% activity) to moderate (2–5% activity) to severe (<1% activity). During the last five years, we have found four novel variants identified in five index patients with no family history of HMA. Three frameshift variants were detected in patients presenting severe HMA and one missense variant was identified in two unrelated patients with a mild phenotype. Methods: Analysis of the *F8* gene was performed in five index patients using PCR followed by Sanger sequencing, after *F8* IVS22 and IVS1 inversions being excluded in severe HMA cases. Bioinformatics analysis was performed with several pathogenicity prediction tools (Alamut Visual, VarSome, VEP and Human Splicing Finder). Results and Conclusions: In the three patients with severe HMA, three different novel *F8* variants were identified: c.1060_1061delCT, p.(Leu354Thrfs∗5), c.4804delC, p.(Gln602Lysfs∗19) and c.3561dupT, p.(Pro1188Serfs∗10). All these variants create a frameshift, leading to a premature termination codon and presumably resulting in non-functional truncated proteins, confirming the patient's phenotypes. The novel *F8* missense variant c.5836G>T, p.(Asp1946Tyr) was identified in two unrelated patients, both with mild HMA. The Asp1946 is a highly conserved amino acid in the FVIII protein. Additionally, physicochemical properties between Asp and Tyr are significantly different, and *in silico* analysis classified it as pathogenic due to the amino acid substitution. Normal mRNA splicing process can also be disturbed due to the creation of a new donor splice site. RNA studies and other functional assays are essential in order to establish this variant clinical significance. Identification of novel pathogenic *F8* variants in HMA patients allows genotype-phenotype correlations, appropriate genetic counseling and new knowledge about the molecular bases of this pathology.

**Financial support:** This work was partially supported by Instituto Nacional de Saúde Dr Ricardo Jorge.

**P40-Presumed *TP53* mosaicism: variants detected using a NGS hereditary cancer multigene panel**

Pedro Rodrigues^1^, Patrícia Theisen^1^, Márcia Rodrigues^2^, Catarina Silva^3,4^, Luís Vieira^3,4^, João Gonçalves^1,4^

1- Unidade de Genética Molecular, Departamento de Genética Humana, Instituto Nacional de Saúde Doutor Ricardo Jorge, Lisboa; 2- Serviço de Genética Médica, Hospital de Santa Maria, Centro Hospitalar Lisboa Norte; 3- Unidade de Tecnologia e Inovação, Departamento de Genética Humana, Instituto Nacional de Saúde Doutor Ricardo Jorge, Lisboa; 4- ToxOmics, Faculdade de Ciências Médicas, Universidade Nova de Lisboa.

Aims/Context: NGS multigene panels are routinely used to identify germline pathogenic variants in cancer susceptibility genes. In addition, NGS allows the identification of low-level mosaicism events that may not be detectable by conventional Sanger sequencing. We describe two cases of presumed *TP53* mosaic variants detected by NGS on blood-derived DNA, and confirmed by ARMS-PCR and Sanger sequencing. Case 1: female, 87 years old, colon cancer at 83 and metachronous breast cancer at 86, no history of familial cancer. Case 2: female, 75 years old, ovarian cancer at 71, local relapse at 74. Methods: NGS using TruSight Cancer Sequencing Panel and TruSight Rapid Capture kit (Illumina) and paired-end sequencing on MiSeq platform (Illumina). Bioinformatic analysis with MiSeq Reporter, Enrichment, VariantStudio, VEP, Alamut Visual, VarAFT, VarSome and IGV. ARMS-PCR and Sanger sequencing were used to confirm the *TP53* variants. Results and Conclusions: Two cases of presumed *TP53* mosaic variants were studied. Case 1: the missense alteration *TP53*: c.764T>G, p.(Ile255Ser) was detected with a variant allele frequency (VAF) of 26% (39/150 reads). This variant is described in ClinVar as a somatic alteration, classified as likely pathogenic. It is not reported in gnomAD and VarSome software classified it as a variant of uncertain significance. Case 2: missense variant *TP53*: c.524G>A, p.(Arg175His) detected with a VAF of 15% (10/58 reads). This variant is described as pathogenic in HGMD Professional, LOVD and ClinVar, in association with Li-Fraumeni syndrome. These two cases seem to represent *TP53* mosaicism, supported by: i) VAF lower than 30%, ii) detection at the sensitivity limit of Sanger sequencing and iii) confirmation by ARMS-PCR. Confirming this hypothesis by studying tumor and other tissue samples and offspring analysis (underway in both cases), is essential for disease diagnosis, assessing recurrence risk and genetic counseling. The hypothesis of acquired aberrant clonal expansion limited to the hematologic compartment, versus a germline variant should be considered in similar cases, and confirmatory methodologies are mandatory.

**Financial support:** Inst Nac Saúde Dr Ricardo Jorge, Centre Toxicogenomics and Human Health (UID/BIM/00009/2013), genomePT (POCI-01-0145-FEDER-022184), and Fundação para a Ciência e Tecnologia.

**P42-Novel TP-PCR based detection of repeats within *AFF2* gene (FRAXE) and accurate homozygous identification**

Cecília Silva^1,2^; Flávia Santos^2,3^; Bárbara Rodrigues^1,2^; Nuno Maia^1,2^; Isabel Marques^1,2^; Rosário Santos^1,2^; Paula Jorge^1,2^

1- Unidade de Genética Molecular, Centro de Genética Médica Doutor Jacinto de Magalhães (CGMJM), Centro Hospitalar Universitário do Porto; 2- Unidade Multidisciplinar de Investigação Biomédica (UMIB), Instituto de Ciências Biomédicas Abel Salazar (ICBAS), Universidade do Porto; 3- Mestrando em Biologia Celular e Molecular, Faculdade de Ciências, Universidade do Porto, Porto

Context: Fragile XE syndrome (FRAXE) is a form of mild to moderate intellectual disability associated with learning deficits, hyperactivity and autistic behaviour. FRAXE, with an estimated frequency of 1/50000, is a trinucleotide repeat disease mostly caused by a GCC expansion in the *AFF2* gene. The broad and unspecific spectrum of FRAXE clinical presentation makes molecular testing essential for a definitive diagnosis. Routinely, the sizing of the *AFF2* GCC repetitive region includes PCR-based and Southern blot (SB) analyses, the latter being a very time-consuming methodology. For that reason, SB is being replaced by alternative approaches such as triplet-repeat primed PCR (TP-PCR). To the best of our knowledge, this method has never been applied to the diagnosis of FRAXE. Methods: A novel TP-PCR was developed using a primer binding upstream the repeat and a (GCC)_5_ primer with a tail (F+R) that also binds to a second region within *AFF2*. The assay was optimized resorting to samples with known allele sizes and validated using DNA samples from 500 unrelated females with a previous uninformative routine PCR testing result. Results: Firstly, the assay correctly sized 100% of the alleles in 475 samples with a normal-range GCC genotype. In the remaining 25 samples originally genotyped as homoallelic, our assay determined 19 with alleles within the normal range, four intermediate alleles and two premutations. Among the first group of 19, four had been incorrectly genotyped due to a SNP near the repetitive region (validated by Sanger sequencing). To verify the correct repeat length in the discrepant samples, they were additionally analysed by SB and the presence of the expanded alleles was confirmed. Conclusions: We describe a simple, accurate and specific tool that can be used in the molecular diagnosis of FRAXE. The assay unambiguously identified homoallelic samples often obviating the need of a second, usually time-consuming technique and representing an attractive alternative for diagnostic laboratories. Furthermore, in six samples this assay correctly identified a putatively pathogenic and unstable allele which had escaped detection with the previously performed PCR.

**Financial support:** UMIB is supported by National Funds through the FCT in the frameworks of the UID/Multi/0215/2016 project UMIB/ICBAS/UP.

**P43- Secondary findings identified in broad-scale sequencing in a Portuguese 915 patient cohort**

Joana Rosmaninho-Salgado^1^, Sérgio B. Sousa^1,2^, Rita Cerqueira^3^, Maria Margarida Venâncio^1,2^, Joaquim Sá^1,2^, Pedro Louro^1^, Sofia Maia^1^, Ana Luísa Carvalho^1^, Pedro Almeida^1,4^, Jorge Pinto-Basto^3^, Jorge M. Saraiva^1,5^

1- Medical Genetics Unit, Hospital Pediátrico, Centro Hospitalar e Universitário de Coimbra, Coimbra, Portugal, 2- Medical Genetics Institute – UC Genomics, Faculty of Medicine, University of Coimbra, Coimbra, Portugal, 3- CGC Genetics, Molecular Diagnostics and Clinical Genomics, CGC Genetics, Porto, Portugal, Porto, Portugal, 4- Faculty of Health Sciences, University of Beira Interior, 5- University Clinic of Pediatrics, Faculty of Medicine, Universidade de Coimbra, Coimbra, Portugal

Context: Controversy concerning the active search for secondary findings (SF) remains since broad massive parallel sequencing emerged in clinical practice. The American College of Medical Genetics (ACMG) recommends the return of SF classified as known or expected pathogenic variants in a set of 59 genes. In this study we review our department's experience in addressing SF. Methods: We included all consecutive results from broad NGS–4813 or 6110 genes panels–or whole exome sequencing concluded between January 2015 and August 2019. The studies were performed by CGC Genetics (860) or within the In2Genome project (55). SF were reported based on the 2013,2016 and 2017 ACMG recommendations. Evaluation of family medical records and segregation studies were performed whenever possible. Results: A SF was reported in 11 out of the 915 analyzed cases (1.2%). The eleven unique SF variants identified were associated with: cancer predisposition–BRCA1, BRCA2 (2), MSH2; cardiomyopathy– MYBPC3 (2), DSC2, KCNQ1, GLA; and familial hypercholesterolemia–APOB, LDLR. None of the index cases had clinical phenotype related to the SF. In two families, relatives already had the clinical diagnosis, one with molecular confirmation (BRCA1) and one without (MSH2). Three additional families had some clinical manifestations within the spectrum of the SF (DSC2, KCNQ1, LDLR). In the six remaining families with a SF, there were no affected relatives. In 3 additional cases, the variant VHL [c.154G>T p.(Glu52Ter)] was found: in 2015 it was reported as SF and now reclassified as a VUS. Discussion: The 1.2% of SF identified in our cohort is consistent with the literature. SF reporting allows the diagnosis and improves the health-care in families that did not meet the clinical criteria for routine diagnostic genetic testing. The absence of affected relatives in 6/11 families may be explained by the fact that some disorders have oligo/polygenic inheritance or that reported variants may turn out to be non- pathogenic. This was the case of the recurrent nonsense VHL variant, which will be discussed in detail. Controversial questions and other examples illustrating our clinical experience and will be presented.

**Financial support:** Part of this cohort was sequenced through the In2Genome project, funded by Centro Portugal Regional Operational Programme (CENTRO-01-0247-FEDER-017800)

**P45- Osteoporosis: Gene interaction between haptoglobin and *HFE* polymorphisms**

Laura Aguiar^1,2,3^, Joana Ferreira^1,2,3^, Maria José Laires^4^, Mário Rui Mascarenhas^3,5^, Ângela Inácio^1,2,3^, Paula Faustino^3,6^, Manuel Bicho^1,2,3^

1- Instituto de Investigação Científica Bento da Rocha Cabral, Lisbon, Portugal; 2- Laboratório de Genética, Faculdade de Medicina da Universidade de Lisboa, Lisbon, Portugal; 3- Instituto de Saúde Ambiental, Faculdade de Medicina da Universidade de Lisboa, Lisbon, Portugal; 4- Centro de Estudos da Performance Humana, Faculdade de Motricidade Humana da Universidade de Lisboa, Lisbon, Portugal; 5- Clínica de Endocrinologia, Diabetes e Metabolismo de Lisboa, Lisboa, Portugal; 6- Departamento de Genética Humana, Instituto Nacional de Saúde Doutor Ricardo Jorge,Lisbon, Portugal

Osteoporosis is a common metabolic bone disease characterized by reduced bone mass and increased risk of fragility fractures. The pathogenesis of this disease is complex and influenced by multiple risk factors, where genetic factors play an important role. Osteoporosis and iron metabolism have an important relationship. Iron overload suppresses osteoblast formation and also stimulate osteoclast resorption of bone, suggesting that polymorphisms in genes affecting iron homeostasis can increase the susceptibility for the development of osteoporosis. In the present study, we aimed to analyse the epistatic relationship between two iron metabolism related genes - haptoglobin (*Hp*) and *HFE* – in osteoporosis. To achieve this, 313 patients with osteoporosis and 450 controls with normal bone mineral density were enrolled. Haptoglobin phenotype was determined by polyacrylamide gel electrophoresis (PAGE) and *HFE* polymorphisms (H63D and C282Y) were evaluated by PCR-RFLP. All statistical tests were performed with SPSS 24.0 software. Results showed that, no significant differences were found between the two populations (patients vs controls) concerning Hp phenotypes or *HFE* (H63D and C282Y) genotypes. However, individuals that have co-inherited the Hp 2.2 and the *HFE*_H63D HH have an increased risk for developing osteoporosis [p = 0.049; OR (95% CI) = 2.509 (1.003–6.279)] (adjusted for age and body mass index). In summary, a significant epistatic interaction was detected between haptoglobin and *HFE* and osteoporosis, where Hp 2.2 in combination with *HFE*_H63D HH genotype appear to increase the risk for developing osteoporosis. Since these genes are related to iron metabolism, the results of this study reinforce an important action of this metabolism in the development of osteoporosis.

**Financial support:** The study was self-funded by the institutions involved.

**P46- Cytogenetic analyses of CLL patients**

Lurdes Torres^1,2,∗^, Susana Lisboa^1,2,∗^, Daniel Santos^1,2^, Joana Vieira^1,2^, Susana Bizarro^1,2^ Nuno Cerveira^1,2^, José M Mariz^3^, Cecilia Correia^1,2^, Manuel R Teixeira^1,2,4^

1- Department of Genetics, Portuguese Oncology Institute of Porto (IPO-Porto), Porto, Portugal; 2- Cancer Genetics Group, IPO-Porto Research Center (CI-IPOP), Portuguese Oncology Institute of Porto (IPO-Porto), Porto, Portugal; 3- Department of Onco-haematology, Portuguese Oncology Institute of Porto (IPO-Porto), Porto, Portugal; 4- Pathology and Molecular Immunology, Institute of Biomedical Sciences Abel Salazar (ICBAS), University of Porto, Porto, Portugal; ∗Equal contribution

Introduction: Chronic lymphocytic leukemia (CLL) is a biologically heterogeneous disease with a highly variable clinical course ranging from indolent to very aggressive. The international prognostic score (CLL-IPI) in order to identify distinct risk groups of CLL patients, integrates clinical variables and also genetic and biological features (del(17p) and/or mutations of the TP53 gene and the somatic hypermutation status of the IGHV gene). The presence of an increased number of cytogenetic abnormalities detected by chromosome banding analyses (CBA) has been associated with more aggressive clinical outcomes, namely the presence of a complex karyotype (CK). We present a series of 55 CLL patients analyzed by CBA and FISH for del(17p). Material and Methods: Bone marrow or peripheral blood samples from 55 CLL patients at diagnosis and before the initiation of any treatment, were cultured with DSP30 and Interleukin 2. CBA was performed with GTL banding. The presence of three or more aberrations was classified as a complex karyotype, whereas High-CK was defined by the presence of five or more. In all cases, FISH analyses with the ON TP53/17 (Leica) probe was performed. Results: A successful karyotype was obtained in 52 of the 55 samples. Thirty-seven out of 52 patients presented an abnormal karyotype (71%), with 16 (43%) classified as CK, and 11 of these (69%) exhibiting High-CK. Deletion of 17p (*TP53*) was observed in 4 out of 55 patients (7%), 3 of which had a CK. Discussion and Conclusions: Routine cell stimulation protocols for CBA in samples of CLL patients allowed to overcome the difficulty in obtaining sufficient metaphases of the malignant clone that rendered a low detection rate of chromosome abnormalities. Recent studies suggest that High-CK may be a predictive marker, independently of the presence of *TP53* aberrations. In our series, karyotype result was obtained in 95% of the samples, with CK in over 40% of the cases. A significant proportion of CK (69%) showed a High-CK. Interestingly, only one case with High-CK exhibited del(17p) detected by FISH. Therefore, CBA before treatment initiation may be useful in the clinical practice.

**P47- Rare autosomal dominant hereditary hemochromatosis associated with *SLC40A1* gene: ferroportin disease or type 4 hereditary hemochromatosis?**

Rita Simão^1^, Rita Fleming^2^, Joana Mendonça^1^, Miguel P. Machado^1^, Luís Vieira^1,3^, Wilson Tavares^1^, João Lavinha^1,4^, Paula Faustino^1,5^

1- Departamento de Genética Humana, Instituto Nacional de Saúde Doutor Ricardo Jorge, Lisboa; 2- Serviço de Imuno-hemoterapia, Hospital de Santa Maria, Centro Hospitalar Universitário de Lisboa Norte, Lisboa; 3- ToxOmics, Faculdade de Ciências Médicas, Universidade Nova de Lisboa, Lisboa; 4- BioISI, Faculdade de Ciências, Universidade de Lisboa, Lisboa; 5- Instituto de Saúde Ambiental (ISAMB), Faculdade de Medicina, Universidade de Lisboa, Lisboa; Portugal

Ferroportin (FPN1), encoded by the *SLC40A1* gene, is the unique cellular iron exporter identified in mammals. FPN1 transfers iron from the intestine and macrophages into the bloodstream. This function is negatively regulated by hepcidin. Mutations in *SLC40A1* may affect FPN1 function, originating distinct autosomal dominant diseases: (i) the Ferroportin Disease (FD), due to loss-of-function mutations, is characterized by decreased iron export from enterocytes and severely affected iron transfer in macrophages, giving rise to a marked iron accumulation in spleen and liver; and (ii) the Type 4 Hereditary Hemochromatosis (HH), resulting from gain-of-function mutations conferring resistance to hepcidin-mediated FPN1 degradation and consequently high cellular iron export. In this study, 335 individuals suspected of having non-classic HH were enrolled. Six genes related with iron metabolism were analysed by SSCP, dHPLC or NGS. The latter used *TruSeq* or *Nextera XT* libraries and a *MiSeq* platform (*Illumina*). Genetic variants found were validated by Sanger sequencing. Predictive consequences at protein level were evaluated using *Polyphen-2* and *SIFT* softwares. From all patients analysed, three *SLC40A1* pathogenic variants were detected in heterozygosity in three women: two missense, c.238G>A, p.Gly80Ser and c.610G>A, p.Gly204Ser; and one deletion, c.485_487delTTG; p.Val162del. These variants had been reported in public databases, but they were not known to be present in the Portuguese population. The p.Gly80Ser and the p.Val162del are FPN1 loss-of-function mutations and were found associated with hyperferritinemia and low transferrin saturation (FD). In contrast, the p.Gly204Ser induced a gain of FPN1 function with a full iron export capacity giving the patient a type 4-HH phenotype, which includes iron overload, hyperferritinemia and high transferrin saturation. Detailed clinical evaluation of the suspected patients are useful to unravel the effect of different mutations in FPN1 function, expression and regulation.

**Financial support:** This work was partially supported by INSA_2013DGH910 and GenomePT (POCI-01-0145-FEDER-022184).

**P48-Evaluation of mathematical indices as tools for distinguishing β-thalassemia trait from iron deficiency anemia in Portuguese females with microcytic anemia**

Bárbara D. Faleiro^1^, João Lavinha^1,2^, Paula Faustino^1,3^

1- Departamento de Genética Humana, Instituto Nacional de Saúde Doutor Ricardo Jorge, Lisboa; 2- BioISI, Faculdade de Ciências, Universidade de Lisboa, Lisboa; 3- Instituto de Saúde Ambiental (ISAMB), Faculdade de Medicina, Universidade de Lisboa, Lisboa; Portugal

Microcytic anemia is a common condition frequently caused by iron deficiency anemia (IDA) or β-thalassemia trait (BTT). Some mathematical indices have been described as fast and inexpensive tools for distinguishing these two conditions. This approach is very useful in mass screening programs especially in countries with limited resources. This study aimed to evaluate the diagnostic performance of 13 distinct indices: RBC, England&Fraser, Mentzer, Srivastava, Shine&Lal, RDW, Ricerca, Jayabose (RDWI), Green&King (G&K), MDHL, MCHD, Sirdah and Ensani. We investigated 102 adult Portuguese female, presenting anemia (Hb < 12 g/dL) and microcytosis (MCV<80fL). The *HBB* gene was screened for pathogenic variants by ARMS or PCR following Sanger sequencing. The iron status was evaluated by standard approaches. IDA was considered when ferritin<12 μg/L and/or transferrin saturation<15%. Two groups were generated: 51 BTT (with one *HBB* variant: c.92+1G>A; c.92+6T>C; c.92+110G>A or c.1188C>T) and 51 IDA, being assured that no individual had simultaneously the two conditions. To determine the performance of the indices, sensitivity, specificity, Youden index (YI) and receiver operating characteristic (ROC) curves were calculated. Due to the high values of AUC (Area Under the *Curve)* from ROC analysis, a cutoff of 0.70 for the YI was established in order to determine the best formulas. We find that the 3 best performing indices to differentiate the 2 groups were RBC (YI = 0.71; AUC = 0.902), RDWI (YI = 0.84; AUC = 0.973) and G&K (YI = 0.82; AUC = 0.972). Our results suggest a similarity with other Mediterranean countries such as Spain and Greece, where G&K and RDWI also performed above our set cutoff. The same is observed in Brazil probably due to its Portuguese ancestry. We conclude that aiming to diagnosis the condition underlying a microcytic anemia in a female population, there is value in using this method to recognize the individuals suspected of BTT and forward them for HbA2 measurement or *HBB* molecular test. In the future, a robust group of male patients should be added to the analysis in order to extrapolate which of these indices would best apply to the whole adult Portuguese population.

**Financial support:** This work was partially funded by INSA_2012DGH720 and ISAMB.

**P49- Chromosomal abnormalities in a cohort of 1341 patients with infertility**

Alexandra Estevinho^1^, Jorge M Saraiva^2^, Serviço de Medicina da Reprodução^3^, C. Pré-Concepcional^4^, Isabel M Carreira^5,6,7^, Eunice Matoso^1,7^

1- Laboratório de Citogenética, Serviço de Genética Médica, Hospital Pediátrico, Centro Hospitalar e Universitário de Coimbra, Portugal; 2- Serviço de Genética Médica, Hospital Pediátrico, Centro Hospitalar e Universitário de Coimbra, Coimbra, Portugal; 3- Serviço de Medicina da Reprodução do Centro Hospitalar e Universitário de Coimbra, Portugal; 4- Consulta Pré-Concepcional da Maternidade Bissaya-Barreto, Centro Hospitalar e Universitário de Coimbra, Portugal; 5- Laboratório de Citogenética e Genómica, Faculdade de Medicina da Universidade de Coimbra, Portugal; 6- CIBB- Centro de Inovação em Biomedicina e Biotecnologia, Universidade de Coimbra, Portugal; 7- iCBR CIMAGO – Centro Investigação em Meio Ambiente Genética e Oncobiologia, Faculdade de Medicina da Universidade de Coimbra, Portugal

About 7% of the couples worldwide suffer from infertility. There are two forms of male or female infertility: primary or secondary. It is a heterogeneous pathology with a complex etiology that includes environmental and genetic factors. Genetic defects account for 50% of the cases and can be of the following categories: chromosome aberrations, DNA copy number variants, single-gene disorders, complex conditions and epigenetic disorders. The aim of this study is to identify the prevalence of chromosome abnormalities in a cohort of 1341 patients with primary or secondary infertility and correlate the reproductive history with the type of abnormality. We studied 1341 patients distributed as: 293 couples with secondary infertility, 65 couples with primary infertility, 544 males with primary sterility and 81 females with primary infertility. Chromosome analysis was performed on blood lymphocytes, using GTG high resolution banding. Complementary molecular studies were performed when needed. The study identified 91 chromosomal abnormalities, 62 were aneuploidies of the sex chromosomes, 24 in the females and 38 in the males. Out of this 31 were mosaics and 8 involved a structural aberration of the sex chromosomes. Autossomal balance rearrangements were observed in 17 patients most associated with secondary infertility. Reciprocal translocations involving sex chromosomes were found only in two infertile male and one female. Our data shows an increased prevalence of chromosome abnormalities along the sub-groups: couples with primary infertility (1,5%), couples with secondary infertility (4,4%), males with identified sterility (9,19%) and female with identified sterility (16,04%). Between couples with secondary infertility the most prevalent cause were balance structural aberrations of the autossomes, mostly affecting the female. When the cause was identified on the male, usually involving spermatogenesis defects, the majority of cases revealed numerical aberrations, reciprocal translocations or men with a 46,XX karyotype. This study supports the usefulness of cytogenetic studies in couples with reproductive failure and the good relation cost-benefit of the karyotype.

**P50- *CYP1B1* mutational screening in a Portuguese cohort of congenital glaucoma patients**

Maria José Simões^1^, Cristina Barroso^1,2^, Ana Luísa Carvalho^3^, Sara Ribeiro^3^, Jorge M Saraiva^3,4^, Conceição Egas^1,2^

1- Genoinseq, Next-Generation Sequencing Unit, Biocant, BiocantPark, Cantanhede, Portugal; 2- Center for Neuroscience and Cell Biology, University of Coimbra, Coimbra, Portugal; 3- Medical Genetics Unit, Hospital Pediátrico, Centro Hospitalar e Universitário de Coimbra, Coimbra, Portugal; 4- University Clinic of Pediatrics, Faculty of Medicine, Universidade de Coimbra, Coimbra, Portugal

Purpose: To determine the prevalence and spectrum of *CYP1B1* mutations causing primary congenital glaucoma in a Portuguese population followed at the Ophthalmology or Medical Genetics Unit of the Coimbra Hospital University Center. Methods: The *CYP1B1* coding regions and intron/exon boundaries were screened by Sanger sequencing in 41 unrelated Portuguese patients with congenital glaucoma. Results: Twelve disease-causing mutations were present in 70.7% (29/41) of the patients. The mutations found corresponded to 5 frameshifts (A179RfsX18, R355HfsX69, T404SfsX30, D449MfsX8 andS464FfsX14), 5 missenses (L378Q, E387K, P437L, I471N and K477E), 1 nonsense (R444X) and 1 non-frameshift (R468_S476dup). All mutations segregated with the disease phenotype, consistent with an inherited autosomal recessive form with complete penetrance. The most frequently mutated allele was the A179RfsX18 frameshift (17/58, 29.3%), being the only mutation found in the exon 2. All other mutations were located at the 5’ end of the exon 3. In terms of allele frequencies, the A179RfsX18 mutation was followed by E387K (10/58, 17.2%), R355HfsX69 (9/58, 15.5%), T404SfsX30 (8/58, 13.8%), R468_S476dup (4/58, 6.9%), S464FfsX14 (3/58, 5.2%) and P437L (2/58, 3.4%). L378Q, R444X, D449MfsX8, I471N and K477E mutations were the least frequent in the studied patients (1/58, 1.7%). Both I471N and K477E are not yet associated with primary congenital glaucoma, are not described in the Genome Aggregation Database (gnomAD) and are predicted as deleterious according to PolyPhen-2 and SIFT. Conclusions: *CYP1B1* is responsible for the disease in almost three-quarters of the patients analysed. These results reinforce the importance of *CYP1B1* screening in PCG patients and at-risk relatives as a first-line genetic test instead of extended studies.

**Clinical Cases**

**P51- Case Report of Parental Transmission of Koolen-De Vries Syndrome**

Paula Rendeiro^1^, Rui Gonçalves^2^, João Parente Freixo^2^, Sofia Sousa Nunes^2^, Raquel Lemos^1^, Cíntia Ventura^1^, Sofia Melo Pereira^1^, Jorge Pinto-Basto^1^, Purificação Tavares^1^, Joaquim Sá^1^.

1- Cytogenetics Laboratory, CGC Genetics, Porto, Portugal; 2- Department of Medical Genetics, Hospital Dona Estefânia – CHLC EPE, Lisbon, Portugal

Koolen-De Vries Syndrome (KDVS) is a rare disorder caused by haploinsufficiency of *KANSL1* gene, either by heterozygous mutation of *KANSL1* or microdeletion on chromosome 17q21.31. Major clinical features include delayed psychomotor development, hypotonia and characteristic facial features. To this day, all individuals reported with KDVS were identified as having the syndrome because of a *de novo* microdeletion/mutation event. Although parent-to-offspring transmission of the syndrome is thought to take place in an autosomal dominant manner, no KDVS individual has been reported to have children of his/her own. In this case report, we present two novel patients with KDVS, in which the microdeletion pattern associated with this syndrome was vertically transmitted, from mother to son. To our knowledge, this is the first case report of a parental transmission of KDVS. Patients were tested using array-based comparative genomic hybridization (array CGH), performed on an Affymetrix platform, Cytoscan 750K. Data analysis was performed on ChAS Software, Affymetrix (NCBI_hg19 reference). Array CGH results revealed an interstitial microdeletion of 477 Kb and 503 Kb at 17q21.31 in proband and his mother, respectively. Diagnosis of both patients was concluded as Koolen-De Vries syndrome. To our knowledge, this is the first case report of a parental transmission of KDVS.

**P52-Phenotypic characteristics of two patients with mandibulofacial dysostosis - Guion Almeida type**

Célia Azevedo Soares^1,2^; Ana Maria Fortuna^1,2^; Gabriela Soares^1^

1- Centro de Genética Médica Jacinto Magalhães, Centro Hospitalar Universitário do Porto, Porto, Portugal; 2- Unit for Multidisciplinary Research in Biomedicine, Instituto de Ciências Biomédicas Abel Salazar/Universidade do Porto, Porto, Portugal

Background: Mandibulofacial dysostosis - Guion Almeida type (MD-GA – MIM#610536) is an autosomal dominant malformation syndrome comprising craniofacial anomalies, microcephaly, abnormalities of the ears and hearing, intellectual impairment and in some cases extracranial malformations and/or short stature. It is caused by heterozygous pathogenic variants or deletions in EFTUD2 gene. With this work we propose to review the phenotypic features of patients with MD-GA and their developmental milestones. Methods: We reviewed the clinical and developmental data of two female patients with MD-GA followed up at Centro Hospitalar Universitário do Porto. Their development was compared with the Haizea-Llevant development table. Results: Both patients had a confirmed molecular diagnosis of MD-GA given by multigene panel, with patient (P) 1 presenting an intronic missense variant on a splicing site and P2 a stop gain missense. Both variants are novel and occurred de novo. Both patients presented typical features of MD-GA such as microcephaly, prominent metopic ridge, upslanted palpebral fissures, prominent nose, micrognathia, and conductive hearing impairment. Only P1 presents the “squared-off” ear lobule typical of MD-GA. Both patients presented a delayed development with P1 acquiring independent walking at age 20 months old (mo), first words at 21 mo, and sentences at 72 mo. P2 acquired independent walking at age 36 mo, first words at 24 mo, day continence at 48 mo. At the age of 6 years P2 does not say sentences or shows continence at night. Conclusion: Our patients have some features typical of MD-GA but most of their features are non-specific. The use of multigene panel was an important strategy to reach a diagnosis, allowing specific genetic counseling for parents and other relatives. In our study we describe the development of our patients, which can be a useful reference to compare with other patients.

**P53- Disruption of***SHH***gene due to an apparently balanced***de novo***t(7;17)(q36;q23) causes holoprosencephaly**

Márcia Rodrigues^1^, Marta P. Soares^1^, Raquel Rodrigues^1^, Rosário Silveira-Santos^1^, Ana Sousa^1^, Ana Berta Sousa^1^

1- Serviço de Genética Médica, Departamento de Pediatria, Hospital de Santa Maria, Centro Hospitalar Universitário de Lisboa Norte EPE, Centro Académico de Medicina de Lisboa, Lisboa, Portugal

Introduction: Holoprosencephaly (HPE) is a structural anomaly of the brain that results from failure of cleavage of the forebrain vesicle to form the cerebral hemispheres. It is associated with malformations of midfacial structures, developmental delay (DD), seizures and pituitary dysfunction, of variable intra and interfamilial severity. HPE is genetically heterogeneous: a chromosomal abnormality is present in ≈25- 50%; a recognizable syndrome in ≈18–25%; and nonsyndromic monogenic HPE in the remainder. Case Report: The patient is the third child of non-consanguineous parents, with irrelevant family history. Pregnancy was uneventful, with normal prenatal ultrasound scans. Amniocentesis was done due to advanced maternal age. Fetal karyotype was 46,XY,t(7;17)(q36;q23)dn; DNA microarray showed no genomic imbalances, and uniparental disomy of chromosome 7 was excluded. After a term caesarean delivery, a male baby was born with microcephaly, bilateral anophthalmia, facial dysmorphism, dysgenesis of corpus callosum and micropenis. Observation of the newborn was suggestive of HPE, most likely caused by disruption of *SHH* gene, which is located in the 7q36.3 region. Peripheral blood karyotype confirmed the prenatal result. Subsequent fluorescence in situ hybridization (FISH) studies, using DNA probes specific for the 7q36.3 (RP11-749O9), which includes *SHH* gene, 7q36.1 (RP11- 163I18) and 17q24.3 (RP11-1150F22) regions, showed FISH signals for RP11-749O9 probe in normal chromosome 7, in der(7) and in der(17). These results indicate that the breakpoint in chromosome 7 is located in 7q36.3 [chr7:155.547.952-155.714.378 (hg19)], which is compatible with disruption of *SHH* gene. Brain magnetic resonance imaging revealed middle interhemispheric variant HPE and dysgenesis of corpus callosum. The patient is now 8 months-old and has moderate DD, pyriform aperture stenosis, feeding difficulties and central diabetes insipidus. Conclusion: This case illustrates the need to correlate phenotype in patients with apparently balanced *de novo* rearrangements with genes located in breakpoint regions, as the pathogenic mechanism can be the disruption of a specific gene located in such regions.

**P54-***RERE***gene related disorder - a glimpse of a new neurodevelopmental syndrome**

Raquel Gouveia Silva^1^; Mariana Soeiro e Sá^1^; Ana Medeira1; Oana Moldovan^1^; Ana Berta Sousa^1^

1- Serviço de Genética, Departamento de Pediatria, Hospital de Santa Maria, Centro Hospitalar Universitário Lisboa Norte, Centro Académico de Medicina de Lisboa, Lisboa, Portugal

Introduction: The *RERE* gene is located in the proximal 1p36 critical region and positively regulates retinoic acid signalling in multiple tissues during embryonic development. RERE-related disorder is characterized by mild to severe developmental delay (DD) and intellectual disability (ID), behavioral issues including autism spectrum disorder, hypotonia, epilepsy, variable coexisting structural anomalies (of the eyes, heart, genitourinary tract and other systems), and neurosensorial hearing loss. It is caused by *de novo* pathogenic variants in *RERE* gene and follows an autosomal dominant pattern of inheritance. To our knowledge, at least 19 affected individuals have been reported in the literature. Clinical Report: We describe a 12-year-old boy, with irrelevant family history, and a 13-year-old girl, who has two maternal first cousins once-removed and a paternal first cousin with ID of unknown origin. Both had DD with language impairment and later ID, which is mild to moderate in the boy, and moderate to severe in the girl. The boy also has generalized hypotonia with muscle weakness, myopathic gait, attention deficit disorder, epilepsy, hydronephrosis and gastroesophageal reflux. The girl was diagnosed with epilepsy (now asymptomatic without medication), pulmonary fibrosis, tracheal stenosis, myopia and hypercholesterolemia. Both patients have minor dysmorphic features. Whole exome sequencing (WES) was performed after extensive investigation, revealing the presence of a *de novo* heterozygous likely pathogenic missense variant in RERE in both cases. Discussion: Proximal 1p36 deletions including *RERE* gene share some characteristics with this disorder, suggesting that haploinsufficiency of RERE may cause at least some of the phenotypic features associated with this CNV. Because individuals with RERE-related disorder can present with a range of clinical phenotypes, and the clinical features are often not specific enough to point at a specific diagnosis, most affected individuals are likely to be diagnosed using NGS panels or WES/WGS. Due to its rarity, ID panels may not include this gene, highlighting the advantages of WES/WGS in the diagnosis of syndromic ID.

**P55- A novel***RAD21***variant in family with mild Cornelia de Lange syndrome phenotype**

Ana Grangeia^1,2^, Rita Quental^1^, Isabel Alonso^2,3^, Helena Santos^4^, Miguel Leão^1,5^

1- Serviço de Genética, Centro Hospitalar Universitário de São João; 2- i3S - Instituto de Investigação e Inovação em Saúde, Universidade do Porto; 3- CGPP e UnIGENe, Instituto de Biologia Molecular e Celular (IBMC), Universidade do Porto; 4- Serviço de Pediatria, Centro Hospitalar de Vila Nova de Gaia; 5- Serviço de Genética, Faculdade de Medicina da Universidade do Porto

Context: Cornelia de Lange syndrome (CdLS) is an autosomal dominant or X-linked developmental disorder characterized by facial dysmorphism, hirsutism, intellectual disability, growth retardation, upper limb and multiorgan anomalies. The clinical features vary widely among affected individuals. Only eight intragenic *RAD21* pathogenic variants and five 8q24.1 deletions encompassing *RAD21* have been identified so far to give rise to CdLS type 4. Results: We report a 12 years old boy with microcephaly, facial dysmorphisms with synophrys, thick highly arched eyebrows, long eyelashes, right palpebral ptosis, depressed nasal bridge, short nose, anteverted nares and long philtrum; hirsutism, atrophic left kidney and claw toe deformity of the left feet with metatarsophalangeal dislocation. Cognitive profile assessment showed a borderline IQ (75). He also presents an attention deficit hyperactivity disorder predominantly inattentive, treated with methylphenidate. His mother showed microcephaly, similar facial dysmorphisms, borderline IQ, hirsutism and brachydactyly. Previous genetic studies, including two NGS panels for mitochondrial genes and microcephaly were normal. Exome sequencing revealed heterozygosity for a novel *RAD21* variant, c.1858A>T (p.Ile620Phe), in exon 14. This variant is not reported in population (gnomAD) or disease (ClinVar and HGMD) databases and bioinformatic analysis predicted the variant as disease-causing. Familial segregation analysis revealed that the variant was inherited from the mother. Conclusion: The patient presented with clinical features previously associated to CdLS type 4 as well as an anomaly of the feet that had not been reported before in CdLS patients. This is the fourth familial case with familial transmission of *RAD21* pathogenic variants and highlights the phenotypic variability of Cornelia de Lange syndrome and also the relevance of molecular diagnosis and genetic counseling to probands and their families.

**P57- Lateral meningocele syndrome presenting as neonatal hypotonia and dysmorphic features**

Maria Abreu^1^, Claudia Falcão Reis^1^, Sónia Figueiroa^2^, Ana Rita Gonçalves^3^, Natália Salgueiro^4^, Margarida Reis Lima^4^, Gabriela Soares^1^

1- Medical Genetics Department, Centro de Genética Médica Jacinto de Magalhães (CGMJM), Centro Hospitalar Universitário do Porto, E.P.E., Porto, Portugal; 2- Division of Pediatric Neurology, Department of Child and Adolescent,Centro Hospitalar do Porto,Porto, Portugal; 3- Molecular Genetics Unit, Centro de Genética Médica Jacinto de Magalhães (CGMJM), Centro Hospitalar Universitário do Porto, E.P.E., Porto, Portugal; 4- GDPN-SYNLAB, Porto, Portugal.

Introduction: Lateral meningocele syndrome (LMS), also known as Lehman syndrome (MIM # 130720), is a rare disorder, with only 14 patients reported in the literature, half of which with molecular diagnoses that consist of heterozygous *NOTCH3* mutations affecting exon 33. LMS should be suspected in patients with multiple lateral spinal meningoceles, distinctive craniofacial appearance, musculoskeletal involvement with hypotonia and joint hyperextensibility, and developmental delay usually with no intellectual disability, with or without congenital malformations and hearing loss.

Clinical Case: a 9 month-old girl was referred to our outpatient clinic for neonatal hypotonia and low weight. She was the only child of a healthy non-consanguineous couple, and there was no family history of congenital anomalies or developmental delay. She had macrocephaly, tall forehead, highly arched eyebrows with medial thinning, depressed nasal bridge, thin upper lip vermillion and downturned corners of the mouth. Brain MRI showed enlarged ventricles. Initial investigations included metabolic study, 15q11.2 MS-MLPA, array-CGH and *DMPK* molecular testing, all normal. Whole exome sequencing (WES) identified a recurrent de novo heterozygous pathogenic variant in *NOTCH3* - c.6692dup (p.Ala2233Glyfs∗9) - providing the diagnosis of LMS. Complete CNS MRI showed multiple lateral mielomeningoceles and ectopic amygdalae. Evaluation by Cardiology, ENT, and Orthopaedics showed no additional findings, and Ophthalmology appointment was scheduled. Throughout follow-up, she overcame the initial developmental delay. Prenatal diagnosis was performed in a subsequent pregnancy, revealing an unaffected foetus.

Discussion: Comparing with previous reports, our patient had a milder presentation, attributed to the absence of congenital malformations as well as to fully preserved hearing. The multiple lateral mielomeningoceles, core feature of LMS, were initially unsuspected. In this case, WES established the diagnosis, enabling a better understanding of prognosis and improving patient management and followup. Molecular diagnosis also allowed specific genetic counselling and prenatal diagnosis.

**P58- Novel***YY1***pathogenic variant: expanding the phenotype of Gabriele-de Vries Syndrome**

Rita Quental^1^, Ana Grangeia^1,2^, Isabel Alonso^2,3^, Miguel Leão^1,4^

1- Serviço de Genética Médica, Centro Hospitalar Universitário de São João, Porto, Portugal; 2- i3S - Instituto de Investigação e Inovação em Saúde, Universidade do Porto, Porto, Portugal; 3- CGPP UnIGENe, Instituto de Biologia Molecular e Celular (IBMC), Universidade do Porto, Porto, Portugal; 4- Serviço de Genética Médica, Faculdade de Medicina da Universidade do Porto, Porto, Portugal

Background: Gabriele-de Vries is a recently described syndrome caused by *YY1* gene dysfunction. Clinical manifestations include developmental delay/intellectual disability, intrauterine growth restriction, feeding problems, and variable functional and morphologic abnormalities of the face, brain, eye, heart, kidney, genitals and skeleton. All pathogenic variants described so far are *de novo* and found in heterozygosity. Case report: We present a 10 years-old boy, first child of non-consanguineous healthy parents. The pregnancy resulted from *in vitro* fertilization and at 34 weeks’ gestation intrauterine growth restriction and a single umbilical artery were detected, leading to caesarean section at 38 weeks. Somatometry at birth was below 5th percentile and Apgar score was 8/10. Admission to the neonatal unit was required due to feeding difficulties. Additional clinical features included neonatal teeth, a left duplex kidney, left inguinal hernia and bilateral iris coloboma. Cranioencephalic MRI at 2 years of age was normal. Cardiac MRI at the age 9 revealed left ventricular noncompaction with normal systolic function. At present, his weight is below P5 and stature between P5-10 (under growth hormone therapy) and he presents mild intellectual disability, attention deficit hyperactivity disorder, retractable testicles, micropenis and abdominal hypopigmented lesions. Etiologic investigation by aCGH revealed a 16q23.1 duplication inherited from his healthy mother, and molecular studies of *SOX2* gene, Cat-eye, Papillorenal and CHARGE Syndromes were normal. Whole exome sequencing trio analysis revealed a *de novo* and novel heterozygous variant in *YY1* gene (c.137_138insT; p.(Glu46Aspfs∗102)) classified as pathogenic. Conclusions: YY1 is a zinc-finger transcription factor with critical roles in development. To date, only 11 patients with pathogenic variants in *YY1* and 13 patients with deletions involving *YY1* gene have been reported. The phenotype of our patient is consistent with that of Gabriele-de Vries Syndrome. Iris coloboma and left ventricular noncompaction were not previously reported, thus enlarging the phenotypic spectrum of this syndrome.

**P59-Newborn whit a derivative chromosome X and ambiguous genitalia**

Laurentino Simão^1^, Sílvia Serafim^1^, Filomena Brito^1^, Cristina Alves^1^, Marisa Silva^1^, Ricardo Peliano^1^, Cristina Ferreira^1^ Bárbara Marques^1^, Sónia Pedro^1^, Juliana Oliveira^1^, Tiago Branco^1^, Hildeberto Correia^1^

1- Departamento de Genética Humana, Instituto Nacional de Saúde Doutor Ricardo Jorge, I.P., Lisboa; 2- Unidade de Neonatologia/Cuidados Intensivos Neonatais, Serviço de Pediatria, Centro Hospitalar do Tâmega e Sousa, E.P.E., Penafiel.

Translocations involving the short arms of the X and Y in human chromosomes are uncommon. One of the primary functions of the X and Y chromosomes is gender phenotype determination. Here we report a newborn female with ambiguous genitalia and abnormal X chromosome. Karyotype was performed using the standard methods and Fluorescence in situ hybridization (FISH) directed for the SRY gene was used for confirmation of the clinical and cytogenetic suspicion. Chromosomal microarray analysis (CMA) was performed using CytoScan HD (Affimetrix) to identified gains/loses on the der(X) chromosome. The analyse revealed one abnormal X chromosome in a female karyotype. Considering the ambiguous genitália clinical information the abnormal X was considered to be compatible with a translocation X/Y. This was confirmed by the presence of signal for the SRY using FISH. CMA allowed to clarify a loss of 12.34 Mb at Xp22.33p22.2 and a gain of 7.41 Mb at Yp11.31p11.2 (ISCN = arr[GRCh37] Xp22.33p22.2(2703632_15050955)x1,Yp11.31p11.2(2650140_10059230)x1). The X deleted region includes several OMIM morbid genes, including CLCN4. Mutations in CLCN4 are associated with intellectual disability and impaired language development, and heterozygous females can be as severely affected as male. The gain on the Y encompasses nine OMIM genes, including the SRY gene, involved in the sexual male development. This additional information can be of great value for the child development. Translocations of segments of Y chromosome containing SRY are described in sexual reversion and true hermafroditism cases, which could explain the reason for referral for the newborn. Nevertheless, translocations between the X/Y chromosomes in females are expected to have a skewed inativation pattern in favour of the abnormal X and X-inativation studies could prove this likelihood. If a normal developmental of the child is observed over time this will be likely due to the preferable inativation of the abnormal X. Presently the child is about 1-year-old and she presents normal uterus, ovarian, and external genitalia, with absence of male gonads. No other clinical features have been identified.

**P62-A novel splicing***FOXP1***variant found in a patient with syndromic intellectual disability through the GenoinVar end-to-end solution**

Maria José Simões^1^, Pedro M Almeida^2,3^, Hugo Froufe^1^, Sílvia Magalhães^1^, Daniel Martins^1^, Sofia Pinto^4^, Catarina Correia^4^, Janet Pereira^5^, Alexandra Oliveira^6^, Sérgio B Sousa^2,7^, Conceição Egas^1,8^

1- Genoinseq, Next-Generation Sequencing Unit, Biocant Park, Cantanhede; 2- Medical Genetics Unit, Hospital Pediátrico, Centro Hospitalar e Universitário de Coimbra; 3- Faculty of Health Sciences, University of Beira Interior, Covilhã; 4- Coimbra Genomics, Biocant Park, Cantanhede; 5- Department of Hematology, Centro Hospitalar e Universitário de Coimbra; 6- Child Developmental Center, Hospital Pediátrico, Centro Hospitalar e Universitário de Coimbra; 7- University Clinic of Genetics, Faculty of Medicine, University of Coimbra; 8- Center for Neuroscience and Cell Biology, University of Coimbra.

Introduction: Clinical complex cases with genetic heterogeneity and/or unspecific phenotype benefit from whole exome sequencing (WES) analysis. We report such a case solved whitin the In2Genome project. Methods: GenoinVar - an end-to-end solution for the discovery of causal variants - was developed in In2Genome. GenoinVar involves Illumina WES and the IDT capture procedure, with above specifications metrics. Sequencing data is processed by an in-house pipeline, and an encrypted database of annotated variants is created. Candidate variants are prioritized in ExomeLoupe, a user-friendly software for variant selection and interpretation. ExomeLoupe interacts directly with the encrypted database enabling users to securely store, analyze and share genetic data in compliance with the GDPR. Results: We report a Portuguese 4-year-old male patient, born to healthy consanguineous parents (r = 1/128), presenting global developmental delay (with autistic features and severe speech delay), unilateral cryptorchidism, intermittent strabismus, relative macrocephaly and dysmorphic features. WES was performed in the proband and candidate variants prioritized, through GenoinVar. The heterozygous variant c.1530+1G>A in *FOXP1* was identified using ExomeLoupe with the filters: global developmental delay (HPO), MAF<0.01 (gnomAD) and deleterious according to predictive tools. The splicing *FOXP1* variant was neither observed in the in-house Portuguese control individuals nor in public databases. We confirmed this result by Sanger sequencing and excluded its presence in both unaffected parents, implying a *de novo* mutation. Discussion: *FOXP1*-related intellectual disability syndrome is a recently delineated condition with wide clinical spectrum in which the patient phenotype integrates well. As in this example, most cases have been identified by WES without previous specific clinical recognition, reinforcing the importance of this test in this group of disorders. GenoinVar has shown to be a user-friendly successful solution, as well demonstrated in this example.

**Financial support:** In2Genome CENTRO-01-0247-FEDER-017800, GenomePT POCI-01-0145-FEDER-022184 and Strategic Project POCI-01-0145-FEDER-007440.

**P63-***PPP2R5D***-related neurodevelopmental disorder - a case report**

Orlando Rodrigues^1^, Joaquim Sá^1^, Fabiana Ramos^1^, Jorge Saraiva^1,2^

1- Medical Genetics Unit, Hospital Pediátrico, Centro Hospitalar e Universitário de Coimbra, Coimbra, Portugal; 2- University Clinic of Pediatrics, Faculty of Medicine, University of Coimbra, Portugal

Context: *PPP2R5D*-related neurodevelopmental disorder is characterized by mild to severe neurodevelopmental delay. The phenotypic spectrum is characterized by pronounced hypotonia with delay in gross motor skills, severe speech impairment with a wide range of disabilities and important macrocephaly. Ataxia and autism spectrum disorder are often reported. Seizures and ophthalmologic abnormalities are present in fewer than half of individuals. Additional anomalies include skeletal, endocrine, and cardiac malformations, each linked to few individuals. At the time no more than 50 individuals have been reported. Case report: A 1-month-old girl, first child of healthy and non-consanguineous parents with no relevant family history, was referred to our Clinical Genetics consultation due prenatal macrocephaly and minor dysmorphisms. Her father has a macrocephaly (+ 2SD) and the diagnosis of Familial Macrocephaly was proposed. Two years later, the girl presented important macrocephaly (+ 4SD) and a global development delay. An extensive etiological investigation was performed, that included chromosome microarray followed by sequencing and multiplex ligation-dependent probe amplification of *PTEN* and *NSD1*. At the age of 14 she has moderate intellectual disability with severe speech delay, ocular torticollis, important macrocephaly (+ 6SD), minor dysmorphisms, toe walking and intentional tremor. At that time, it was performed an exome sequencing, which found a heterozygous de novo pathogenic variant in *PPP2R5D* gene [c.592G>A p.(Glu198Lys)] that yielded the diagnosis. Conclusion: This case report makes us aware of a condition recently associated with global development delay/intellectual disability and some consistent phenotypic aspects. Also, it reinforces the importance of a genetic consultation follow-up in patients with no molecular diagnosis, underlining the power of the new diagnostic tools in the Clinical Genetics field. At the time, it is difficult to establish a specific follow-up plan, since the number of affected individuals is limited and there is no description about the disease natural history.

**P64-A case of a novel BCL11A variant associated with Dias-Logan syndrome**

Sofia Maia^1,2^, Marta Marques^1^, Carolina Duarte^3^, Jorge M. Saraiva^1,4^

1- Medical Genetics Unit, Hospital Pediátrico, Centro Hospitalar e Universitário de Coimbra, Coimbra, Portugal; 2- University Clinic of Genetics, Faculty of Medicine, University of Coimbra, Coimbra, Portugal; 3- Neurodevelopment Unit, Pediatric Department, Centro Hospitalar do Baixo Vouga, Aveiro, Portugal; 4-University Clinic of Pediatrics, Faculty of Medicine, University of Coimbra, Coimbra, Portugal

Introduction: Dias-Logan syndrome (MIM #617101) is a novel autosomal dominant disorder caused by *de novo BCL11A* mutations. BCL11A protein is a transcriptional repressor of fetal hemoglobin and a transcription factor associated with the BAF SWI/SNF chromatin remodeling complex. The protein is highly expressed in fetal human brain, where it has significant roles (migration control of neurons, axon branching and dendrite outgrowth). Dias-Logan syndrome is characterized by global developmental delay, cognitive dysfunction, behavioral features, joint laxity, strabismus, microcephaly and significantly elevated fetal hemoglobin without recognizable dysmorphic features. Clinical description: We report a girl who is the second child of a non-consanguineous healthy couple and single case in the family. She was born prematurely at 35 weeks of pregnancy after labor induction due to oligohydramnios detected at 27 weeks of gestation and fetal echodoppler with inverted flow. She has moderate developmental delay, prenatal microcephaly [head circumference at birth and at the age of three years and five months below the 1^st^ centile (-3SD and -2.8SD, respectively)], congenital heart defect (atrial septal defect and congenital stenosis of the pulmonary artery branches), cerebellar vermis hypoplasia, joint hypermobility, facial dysmorphisms and persistence of fetal hemoglobin (16.3%). Results and discussion: A next-generation panel of 6110 genes was performed and identified the *de novo* likely pathogenic variant *BCL11A* (NM_022893.3) c.1847dup p.(Leu617Profs∗18). This frameshift variant has not been previously described in the literature and is predicted to introduce a premature STOP codon. Eleven of the 15 patients reported so far in the literature harbor frameshift or nonsense mutations. The remaining four patients harbor missense mutations and for three of them *in vitro* functional assays have been performed and results were consistent with a loss of function. To our knowledge, this is the first report of a Portuguese patient diagnosed with Dias-Logan syndrome and it will help delineate the mutational and clinical spectrum of this novel and rare syndrome.

**P65-Discordant dichorionic diamniotic (DCDA) twins: clinical and cytogenetic characterization of a foetus with structural chromosomal aberrations**

Sílvia Pires^1^, Cristina Candeias^1,2^, Maria do Céu Rodrigues^3^, Gabriela Soares^1^, Natália Oliva-Teles^1,2^

1- Centro de Genética Médica Doutor Jacinto Magalhães/Centro Hospitalar Universitário do Porto E.P.E., Porto; 2- Unidade Multidisciplinar de Investigação Biomédica/Instituto de Ciências Biomédicas Abel Salazar, Universidade do Porto (UMIB/ICBAS-UP); 3- Consulta Externa de Diagnóstico Pré-natal, Centro Materno Infantil do Norte/Centro Hospitalar Universitário do Porto E.P.E., Porto;

Introduction: Different structural chromosomal aberrations involving more than two chromosomes in the same individual are extremely rare. In these cases, particularly in prenatal diagnosis settings, a good ultrasound description is very important since carriers may display various phenotypes, ranging from normal to a foetus with different congenital abnormalities, depending on the size of deletion/duplication resulting from the chromosomal rearrangement and the chromosomes involved. These rearrangements may be *de novo* or familial. The minimum presumed risk of phenotypic abnormality for *de novo* multiple chromosome rearrangements identified prenatally may be estimated as the additive risk of the presumed number of identifiable chromosome breakpoints. Methods: A 35-year-old multiparous woman with a dichorionic diamniotic (DCDA) twin pregnancy referred to our Centre at 19 weeks’ gestation for prenatal cytogenetic studies; foetus 1 presented with severe hydrocephalus and growth restriction and foetus 2 with positive biochemical screening. Conventional GTL-banding karyotyping was performed on metaphase mitotic cells obtained from amniotic fluid according to standard procedures. ArrayCGH was requested. Results: Foetus 1 revealed a 46,XY,t(3;6)(p13;p23),del(7)(q31.2q32.3) karyotype and foetus 2 was 46,XY. ArrayCGH confirmed del(7q). Parent's karyotypes were normal. Selective feticide was successfully performed on the hydropic foetus. Discussion: The authors present the cytogenetics and clinical findings of a *de novo* balanced chromosomal translocation associated with a *de novo* interstitial deletion and compare them with previous studies presenting with similar chromosomal breakpoints and genes mapped to that region.

**P66-Non Invasive Prenatal Testing (NIPT): four years’ experience, interesting cases and challenges**

Mariana Ferreira^1^, Luísa Bastos^1^, Lurdes Caloba^1^, Ligia S. Almeida^1^, Rita Cerqueira^1^, Jorge Pinto-Basto^1^

1- Laboratório de Diagnóstico Molecular e Genómica Clínica, CGC Genetics/Unilabs

Background: In the last years, NIPT has become a widespread method of screening for common autosomal (13, 18, 21) and sex chromosome aneuploidies. It is based in the massive parallel sequencing of cell-free DNA (cfDNA) from maternal plasma, which contains a mixture of small maternal and placental DNA fragments. Although NIPT has been shown to be highly accurate for the detection of these aneuploidies, a small percentage of women have low confidence or nonreportable results. Methods: A revision of the cases received during the last 4 years is presented for evaluation of interesting and challenging data. The test was performed on maternal whole blood samples, collected in Streck tubes and received up to 5 days after collection. cfDNA was extracted and analyzed by whole genome sequencing (NextSeq_Illumina). Results: During these 4 years, several interesting cases came to our attention, with either low confidence or nonreportable results. Main reasons for low confidence results were low fetal fraction, high maternal weight and borderline results to the cutoff value (mainly for sex chromosomes). Presence of vanishing twins and maternal chromosomal alteration were also identified. Among the unreportable results, the main reason for this finding was a “no call”, meaning the analysis software was not able to give a result. Conclusion: There are several reported biological causes for these low confidences or nonreportable results that ultimately can lead to false positive and false negative results. Those include confined placental mosaicism, vanishing twins, maternal incidental findings and low fetal fraction. So far, to our knowledge in these 4 years’ experience, no false negative results were reported, and false positive results were mainly related with borderline values for sex chromosomes. Professional societies recommend offering NIPT to women at increased risk of fetal chromosome aneuploidies, as part of a contingent screening or even for all women. The knowledge of outcomes, when other diagnostic methods are performed, will increase not only the knowledge about doubtful profiles, but also reinforce the validation of this screening test.

**P67-Nonsyndromic juvenile myelomonocytic leukemia with a somatic PTPN11 mutation in a 4-year-old boy**

Lígia S Almeida^1^, Luísa Bastos^1^, Rita Cerqueira^1^, Jorge Pinto Basto^1^

1- Laboratório de Diagnóstico Molecular e Genómica Clínica, CGC Genetics/Unilabs – Porto, Portugal

Background: Juvenile myelomonocytic leukemia (JMML) is an aggressive pediatric myelodysplastic syndrome/myeloproliferative disorder characterized by malignant transformation in the hematopoietic stem cell compartment with proliferation of differentiated progeny. The only curative therapy is hematopoietic stem cell transplant. Mutations in NF1, NRAS, KRAS, PTPN11 and CBL (“Ras pathway”) currently allow for a molecular diagnosis in 85% of patients. We present a 4-year-old boy with acute myeloid leukemia (with monosomy 7) with features suggestive of primary juvenile myelomonocytic leukemia in which whole exome sequencing was performed. Method: Whole exome sequencing was performed by capture of target regions using oligonucleotide probes (V6, Agilent Technologies) and subsequent next generation sequencing (NextSeq, Illumina). Alignment and variant calling was performed using the BWA and GATK, respectively. Variants with MAF<1% were filtered and processed with bioinformatic analysis tools to assess its pathogenicity and potential to explain the clinical phenotype. Results: The NM_002834.3:c.227A>G p.(Glu76Gly) likely pathogenic variant was detected in heterozygosity in the PTPN11 gene in DNA from peripheral blood. This variant has been previously reported as a germline variant in patients with Noonan syndrome but also as a somatic mutation in colon cancer and acute myeloid leukemia. After transplant, DNA from a novel blood sample and buccal swab tested negative for the detected variant. Additionally, the variant was also not present in DNA from the parents. Conclusions: We report a JMML case with a proven somatic variant in PTPN11. The absence of the variant in a new blood sample after treatment supports that the detected variant is somatic, further reinforced by the absence of the variant in a buccal swab sample and in DNA from the parents Additionally, our case illustrates the importance of obtaining germline tissue. This is of critical importance to further clarify the origin of the variant and treatment options as in several cases, patients have inherited syndromes that predispose to the development of JMML.

**P68-X-chromosome terminal deletion including the FMR1 gene in a family with fragile X-syndrome and a full mutation.**

Liliana Gonçalves^1^, Lígia Lameiras^1^, Marisa Teixeira^1^, Rita Cerqueira^1^, Teresa Kay^2^, Jorge Pinto-Basto^1^

1- Laboratório de Diagnóstico Molecular e Genómica Clínica, CGC Genetics/Unilabs; 2- Hospital do Divino Espírito Santo Ponta Delgada, E.P.E.

Background: Most cases of fragile X syndrome result from an expansion of CGG repeats in the FMR1 gene; deletions and point mutations of FMR1 are much less common. Terminal deletions on the X chromosome in female patients have already been described as a promising explanation for POF, especially when involving the Xq28 region. Case Report: In this study we report one case of an asymptomatic female referred due to family history of three brothers with clinical diagnosis of Fragile X-syndrome. Previous molecular study by msTP-PCR for FMR1 gene, performed in the mother (also asymptomatic), revealed the presence of a normal and a full mutation allele, most probably the cause of the segregation of this condition in the affected brothers. msTP-PCR for FMR1 gene was performed and revealed the presence of an intermediate allele with 51 (± 3) CGG repeats in the analyzed region of the FMR1 gene and no amplification was obtained for methylated alleles, suggesting the presence of a deletion involving at least the promoter region of the FMR1 gene. To confirm this, deletion/duplication analysis was performed by MLPA analysis (ME029- B3, MRC Holland) and a hemizygous deletion that includes at least the FMR1 gene and other four downstream genes in the X chromosome terminal region (AFF2, IDS, MTM1 and FLNA) was detected. Considering the overall obtained results in this family, the detected deletion was most likely resultant from instability of the full mutation allele transmitted by the mother. Deletions of different sizes in the Xq27.3-Xq28 region have already been described in the literature including not only the FMR1 gene but extending to the terminal region of chromosome X, leading to a wide variability of phenotypes, not only because of the deleted region, but also because of a skewed X inactivation. In this case, despite the hemizygosity of FMR1 gene, she does not present fragile X syndrome features, since the normal Xchromosome is not subject to methylation. However, it is not possible to exclude that she may develop symptoms of premature ovarian failure (POF).

**P69-Array-CGH in prenatal diagnosis – cohort of a public laboratory in the Centre of Portugal**

Mariana Val^1^, Susana I. Ferreira^1^, Luís M. Pires ^1^, Nuno Lavoura ^1^, Alexandra Mascarenhas ^1^, Marta Pinto ^1^, Isabel M Carreira ^1,2,3^, Joana Barbosa de Melo ^1,2,3^

1- Laboratório de Citogenética e Genómica, Faculdade de Medicina da Universidade Coimbra, Coimbra, Portugal; 2 - iCBR-CIMAGO – Centro de Investigação em Meio Ambiente, Genética e Oncobiologia, Faculdade de Medicina da Universidade Coimbra, Coimbra, Portugal; 3 - CIBB - Centro de Inovação em Biomedicina e Biotecnologia (CIBB), Faculdade de Medicina da Universidade de Coimbra, Coimbra, Portugal

Context: Array comparative genomic hybridization (array-CGH) is a useful approach to detect submicroscopic imbalances, known as copy number variants (CNVs). It was shown that in prenatal cases with a normal karyotype, the array-CGH revealed additional relevant information in ∼6% of the prenatal cases with ultrasound abnormalities and in ∼1.7% of cases with indications for invasive prenatal diagnosis (PND). According to these results, array-CGH is recommended to be performed as the first tier genetic test when fetal ultrasound abnormalities are detected. Methods: A cohort of 822 prenatal cases was studied by array-CGH, due to different clinical indications (such as ultrasound abnormalities, increased nuchal translucency, positive biochemical screening). Array-CGH was performed using Agilent 4x180K or 8x60K platform. Results: 724 prenatal cases were studied by array-CGH as the remaining showed a positive result to rapid aneuploidy detection (RAD). Regarding the CNVs detected, pathogenic CNVs were identified in 7.6% of studied cases and in 12% of samples at least one likely pathogenic CNV or a variant of uncertain significance (VOUS) was observed. 12.7% of pathogenic CNVs revealed a deletion or duplication of the 16p11.2 region, being the most frequently altered region detected. The presence of ultrasound abnormalities was the most common clinical indication among the cases with pathogenic CNVs (65.5%). Array-CGH also allowed identifying mosaicism in 1.1% of studied cases. Conclusions: These results show the fundamental role of array-CGH in PND to detect clinical relevant CNVs that would not be found using conventional cytogenetic methodologies, more quickly, allowing to increase the diagnostic yield and decrease the turnaround time.

**P70-Familial 18q23 deletion: the same alteration with different phenotype - a case report**

Patrícia Paiva^1^, Susana I. Ferreira^1^, Lúcia Simões^1^, Luís M. Pires^1^, Ana Jardim^1^, Pedro Almeida^5^, Sérgio Sousa^5^, Eunice Matoso^4^, Joana B. Melo^1,2,3^, Isabel M Carreira^1,2,3^

1- Laboratório de Citogenética e Genómica, Faculdade de Medicina da Universidade Coimbra, Coimbra, Portugal; 2 - ICBR-CIMAGO – Centro de Investigação em Meio Ambiente, Genética e Oncobiologia, Faculdade de Medicina da Universidade Coimbra, Coimbra, Portugal; 3 - CIBB - Centro de Inovação em Biomedicina e Biotecnologia (CIBB), Faculdade de Medicina da Universidade de Coimbra, Coimbra, Portugal; 4 – Laboratório de Citogenética, Hospital Pediátrico de Coimbra, Centro Hospitalar e Universitário de Coimbra, Coimbra, Portugal; 5 – Serviço de Genética Médica, Hospital Pediátrico de Coimbra, Centro Hospitalar e Universitário de Coimbra, Coimbra, Portugal

We present a 18q23 terminal deletion identified in an 1 year old boy with cardiac anomalies, pulmonary stenosis moderated, short neck, facial dysmorphisms and feet malformations. Array-based comparative genomic hybridization (aCGH), using a 180K Agilent oligonucleotide microarray revealed a 18q terminal deletion of about 2.8 Mb including 2 genes reported in OMIM Morbid Map (*CTDP1* and *TXNL4A*). Cytogenetic analysis with GTG-banding was carried out in the proband and in his parents and revealed normal results. The deletion wasn’t found in the kariotypes of proband and parents, since it was below of the karyotype resolution limit. Fluorescence *in Situ* hybridization (*FISH*) analysis using the subtelomeric probe for 18q23, confirmed the deletion in the proband and showed that the father carried the same alteration as the child. Since the father has no clinical signs, array-CGH analysis was performed and the same identical deletion was observed. The phenotype associated with a 18q terminal deletion has been well described and can include mental retardation and development delay, hypotonia, short stature, congenital aural atresia, abnormal genitalia, facial dysmorphisms, foot malformations and delayed myelination. The incidence of these clinical features in different patients with 18q deletion is variable. In the present study, father and son have the same deletion, although they are phenotypically different. The proband has many of the typical clinical features associated with the 18q deletion syndrome and the father is not clinically recognizable as having a 18q-syndrome phenotype. It was previously reported a child with hypotonia and other features associated with 18q-syndrome at the age of 2, but all of them were absent at the age of 4. In conclusion, our case report demonstrates that genotype/phenotype correlation studies are very difficult to establish on the 18q- syndrome and also shows the dilemma that exists in the categorization of patients with 18q deletions.

**P71-The challenges of pseudogenes in genetic diagnosis by Next Generation Sequencing**

Andreia Pinto^1^, Filipa Melo^1^, Mariana Ferreira^1^, Luísa Bastos^1^, Sofia Marques^1^, Lígia S Almeida^1^, Rita Cerqueira^1^, Jorge Pinto Basto^1^

1- Laboratório de Diagnóstico Molecular e Genómica Clínica, CGC Genetics/Unilabs – Porto, Portugal

Background: Targeted next-generation sequencing (NGS) enables rapid identification of genetic variation in a large subset of genes with high confidence. However, since current methodology lack the sensitivity to distinguish reads that come from homologous parts of the genome, it is a challenge to work with genes with paralogues or pseudogenes. We present three case studies, with variants in *HYDIN* and *PMS2* genes, known to have pseudogenes. Methods: Next generation sequencing (Illumina) of genomic DNA was performed upon oligonucleotide-based target capture (Agilent Technologies). Alignment and base calling were performed with BWA and GATK, respectively. Results: For two different cases, NGS was performed and revealed two variants in exon 11 and 13 of the *HYDIN* gene with an allele frequency of 44.5% and 42.4%, respectively. Blast analysis was performed to identify possible mismatches and to design specific primers to confirm the result obtained in the NGS analysis; the mismatches identified were present in the NGS data with a heterozygosity of nearly 50% as well. Sanger sequencing was performed and confirmed the presence of the variants in homozygosity for both cases. Segregation analysis confirmed the heterozygosity in both parents. A similar approach was used in a third case regarding the *PMS2* gene. In the NGS data analysis, a variant with an allele frequency of 29% was detected. Specific Sanger sequencing was performed confirming the presence of the variant in heterozygosity. Conclusions: Due to high sequence similarity, sequenced reads arising from a pseudogene may be misaligned to the functional gene and result in false positive variant calls or false negative results. Furthermore, variants in regions of homology to a pseudogene are difficult to validate with Sanger sequencing. Here we show that with NGS technology one should be cautious interpreting variants in genes with pseudogenes/homology. Given the limitations of this methodology, an integrated data analysis and the use of other strategies should be addressed to reliable detect the presence and zygosity of variants identified in regions with high homology.

**P72-Non-Invasive Prenatal Test Implementation in a public laboratory in the Centre of Portugal**

Luís M Pires^1^, Daniela Oliveira^2^, Mariana Val^1^, Susana I Ferreira^1^, Cláudia Pais^1^, Nuno Lavoura^1^, Cristina Pita^3^, Joaquim Sá^2^, Maria M Venâncio^2^, Lina Ramos^2^, Luís Abreu^3^, Miguel Branco^3^, Maria Céu Almeida^3^, Ana I Rei^3^, Sofia Franco^4^, Filomena Coelho^4^, Helena Gonçalves^4^, Fabiana Ramos^2^, Jorge Saraiva^2^, Eulália Galhano^3^, Joana B Melo^1,5,6^, Isabel M Carreira^1,5,6^

1- Laboratório de Citogenética e Genómica – Faculdade de Medicina da Universidade de Coimbra, Portugal; 2- Serviço de Genética Médica, Centro Hospitalar e Universitário de Coimbra, Portugal; 3- Centro de Diagnóstico Pré-natal, Maternidade Bissaya Barreto, Centro Hospitalar e Universitário de Coimbra, Portugal; 4- Consulta de Diagnóstico Pré-natal, Maternidade Daniel de Matos, Centro Hospitalar e Universitário de Coimbra, Portugal; 5- CIBB- Centro de Inovação em Biomedicina e Biotecnologia, Universidade de Coimbra, Portugal; 6- iCBR CIMAGO – Centro Investigação em Meio Ambiente Genética e Oncobiologia

Objectives/Background: Non-invasive prenatal testing (NIPT) has been widely used to detect common fetal chromosome aneuploidies (T13, T18 and T21) and has expanded to sex chromosome aneuploidies. In this work we review the performance of the implementation of a NIPT test from blood cfDNA at our center. Methods: Retrospective analysis of NIPT Clarigo (Agilent Technologies), using MiSeq platform (Illumina), in pregnancies with moderate risk for common fetal chromosome aneuploidies. Maternal blood samples were collected from February 2019 to September 2019 in Coimbra University Hospital Center (CHUC). Control samples, in which the laboratory was aware of the aneuploidy status were not included. Results: From received NIPT samples, 2% had positive NIPT result, (80% T21 and 20% T13), 90,5% were negative and 7,5% were inconclusive. NIPT positive results were validated by invasive test in 60% of the cases, one mother decided to continue pregnancy and in one case there was a spontaneous fetal loss (T13). There weren’t known false-negative results (sensitivity = 100%). Of the 7,5% inconclusive cases, 42% were due to hemolysis; 21% to low fetal fraction; 16% to possible vanishing twin and 21% to an unknown cause. A new blood sample was required in 50% of the inconclusive cases with conclusive result in 87,5% of the resampled cases. NIPT was also performed in pregnancies with Body Mass index greater than 30 (22% of the cases) with conclusive result in 98% of them. A relevant incident finding on maternal X chromosome was identified in 2,4% cases (2% with a dup Xp; 0,4% with a maternal del Xp, confirmed by Array-CGH, with possible pathogenic impact on a male fetus and 0,4% with a turner mosaic). Conclusion: Our NIPT results, based on NGS, showed good performance for detecting T13, T18, and T21. Nevertheless, the number of samples is still very small for an accurate assessment of performance of screening. Our results demonstrate that targeted NIPT using MiSeq platform could be an alternative for centers with low NIPT sample flow. The implementation of the NIPT can contribute to reduction in the number of invasive exams and eventual associated fetal losses.

**P73-Del 8p in Chronic Lymphocytic Leukemia: a case report**

Pedro Botelho^1^, Marta Souto^1^, Regina Arantes^1^, Margarida Costa^2^, Márcia Martins^3^, Osvaldo Moutinho^4^, Manual Cunha^3^, Rosário P. Leite^1^

1- Laboratório de Genética, Centro Hospitalar Trás-os-Montes e Alto Douro, Vila Real, Portugal; 2- Serviço de Hematologia, Centro Hospitalar Trás-os-Montes e Alto Douro, Vila Real, Portugal; 3- Consulta de Genética, Centro Hospitalar Trás-os-Montes e Alto Douro, Vila Real, Portugal; 4- Serviço de Ginecologia/Obstetrícia, Centro Hospitalar Trás-os-Montes e Alto Douro, Vila Real, Portugal

Context and Objectives: In the last few years, several studies using appropriate stimulation of chronic lymphocytic leukemia (CLL) cells have enhanced the performance of conventional karyotyping, detecting additional chromosomal alterations of potential prognostic significance. Chromosomal abnormalities in CLL are detected in up to 80% of patients, they have a known prognostic value and play an important role in CLL pathogenesis and evolution, determining patient's outcome and therapeutic strategies. According to guidelines, in general practice, Fluorescence in situ Hybridization (FISH) should always be performed, identifying the most common chromosomal rearrangements (trisomy of chromosome 12 and deletions in loci located on chromosomes 13q14, 11q22-q23 and 17p13) in peripheral blood lymphocytes and conventional cytogenetic not generally indicated. Methods: The authors present a case of an 83-year old woman with CLL and previous cytogenetic study (FISH and karyotype) of trisomy 12 in 2015. FISH panel and cytogenetic analysis were performed after 4 years. FISH and conventional cytogenetics were performed according to the protocols established in the laboratory. Results and Conclusion: FISH results detected trisomy 12 in 53% of cell, with a similar incidence from the previous analysis. Conventional cytogenetic reveal the karyotype: 46,X,del(8)(p11),+12[7]/46,XX[11]. The del 8 appears in this analysis. It has not yet been determined whether del 8p plays any role in driving drug resistance, however, it was already reported to predict an increased risk for disease progression in the setting treatment. Also, a recent large study identified del 8p as recurrent event significantly associated with 17p and CLL. The present case highlights the importance of conventional cytogenetics in the study of CLL patients. FISH could not detect the del8p, which were very important to explain the partial response to the therapy and uncover the patient adverse prognostic.

**P74-Third case of Cantú Syndrome associated to a novel KCNJ8 variant**

Sofia Nunes^1^, Mafalda Melo^1^, Márcia Rodrigues^2^, Conceição Trigo^3^, Susana Barbosa^4^, João Freixo^4^, Teresa Kay^1^, Diana Antunes^1^

1- Medical Genetics Department, Hospital de Dona Estefânia, Centro Hospitalar Universitário Lisboa Central, EPE; 2- Medical Genetics Department, Hospital de Santa Maria, Centro Hospitalar Lisboa Norte, EPE; 3- Pediatric Cardiology, Hospital de Santa Marta, Centro Hospitalar Universitário Lisboa Central, EPE; 4- Centro de Genética Médica Preditiva e Preventiva

Case report: Here we report a case of a 20-month-old female referred to our clinic for prenatal polyhydramnios and facial dysmorphic features. At clinical observation it was noted coarse facies, low frontal hairline and generalized hypertrichosis predominantly on both arms. Cardiac evaluation showed dysplastic aortic valve. A possible diagnosis of lysosomal storage disease was suspected but an extensive metabolic evaluation excluded this hypothesis. Clinical reevaluation at 8 yrs, showed short stature (< 5th percentile), joint hyperlaxity, pectus carinatum, astigmatism and hyperopia, as well as learning difficulties. Facial features became more evident, resembling Cantú syndrome. Sanger gene sequencing of *ABCC9* gene was normal. After reevaluation at 14 yrs, cardiac evaluation revealed hypertrophic cardiomyopathy and pulmonary hypertension. By that age, she developed panhypopituitarism (growth hormone deficit, hypogonadotropic hypogonadism, hypothyroidism, hypocortisolism) and relapsing polychondritis. Clinical exome was performed which did not reveal pathogenic variants, however targeted reanalysis of *KCNJ8* gene showed a novel variant (p.E331K) *de novo* predicted to be pathogenic.

Discussion: Cantú syndrome is a rare autosomal dominant disorder characterized by hypertrichosis, a distinctive facial appearance, heart defects and several other abnormalities caused by mutations in *ABCC9* and *KCNJ8* genes. More than 30 patients with pathogenic variants on *ABCC9* have been previously reported. Pathogenic variants in *KCNJ8* gene, which encodes a member of the inward rectifier potassium channel family, were only previously reported in two patients. Both of them shared phenotypical characteristics with our patient like hypertrichosis, coarse facies, cardiomegaly. Endocrinological abnormalities were not previous reported. Conclusion: To our knowledge, this is the third case report of Cantú syndrome associated to *KCNJ8* gene, reinforcing its contribution to the phenotype.

**P76-Do we really need to know fetal gender for array-CGH in prenatal diagnosis?**

Susana I Ferreira^1^, Mariana Val^1^, Luís M Pires^1^, Nuno Lavoura^1^, Joana B Melo^1,2,3^, Isabel M Carreira^1,2,3^

1- Laboratório de Citogenética e Genómica – Faculdade de Medicina da Universidade de Coimbra, Portugal; 2- CIBB- Centro de Inovação em Biomedicina e Biotecnologia, Universidade de Coimbra, Portugal; 3- iCBR CIMAGO – Centro Investigação em Meio Ambiente Genética e Oncobiologia, Faculdade de Medicina da Universidade de Coimbra, Portugal

Array Comparative Genomic Hybridization (aCGH) is currently recommended for all fetus with major structural anomalies after obstetric ultrasound. The guidelines also recommend rapid aneuploidy detection (RAD) to be performed before array-CGH analysis, as it not only excludes a common aneuploidy but also indicates fetal gender, for appropriate array-CGH control hybridization. We retrospectively reviewed the 822 prenatal samples received in our laboratory, for 180K oligonucleotide array-CGH analysis, consisting of amniotic fluids (AF-498), chorionic villus (CVS-219), fibroblasts (90) and cordocentesis (15). In CVS, RAD gave a positive result in 28.7% of the samples, with 37.1% positive for trisomy 21. In AF, only 3.4% of samples revealed a RAD positive result, 47% of which for trisomy 21. All the 724 samples with a normal result for RAD proceeded to aCGH analysis with a sex matched commercial control. We observed 25 imbalances on sex chromosomes, 19 on the X chromosome, 4 on the Y chromosome, a triple X and a XYY, both in miscarriage products whose gender was determined by visual examination. Considering the Y chromosome imbalances, 3 were duplications - one paternal, 2 whose origin was not determined- and the other was an Y rearrangement previously observed by conventional cytogenetics. On the X chromosome we observed 16 imbalances on the short arm – 6 deletions (3 maternal, 2 paternal, 1 unknown) and 10 duplications (6 maternal, 4 paternal) – and 3 imbalances on Xq – 1 *de novo* deletion, 1 paternal duplication and 1 paternal triplication. Only 2 maternal deletions on Xq28 were considered to be pathogenic in male fetus, and one proceeded to pregnancy interruption. Taking into account our results we consider that: in CVS RAD should be performed before aCGH, as it gives a high positive rate for aneuploidies; in AF, RAD should be performed before array-CGH only when ultrasound anomalies are indicative of an aneuploidy. When an aneuploidy is not suspected or was excluded, fetal gender can be given by ecographic evaluation or if not determined, samples can by blindly hybridized with a control, as we can always infer copy number changes on sexual chromosomes.

**P77-Germline *ABL1* variant identified in a Nepalese girl with congenital heart defects and skeletal malformations syndrome: a case report**

Mafalda Melo^1^, Tiago Rito^2^, Isabel Freitas^2^, Jorge Pinto-Basto^3^, Teresa Kay^1^, Diana Antunes^1^;

1- Unidade de Genética Médica, Área da Mulher, Criança e Adolescente, Hospital Dona Estefânia, Centro Hospitalar Universitário de Lisboa Central, Lisboa, Portugal; 2- Serviço de Cardiologia Pediátrica, Hospital Santa Marta, Centro Hospitalar Universitário de Lisboa Central, Lisboa, Portugal; 3- CGC Genetics, Porto, Portugal

Germline variants in *ABL1* gene were recently identified in six patients with the previously unreported congenital heart defects and skeletal malformations syndrome. This is an autosomal dominant disorder characterized by congenital atrial and ventricular septal defects, with aortic root dilation in adulthood; and variable skeletal defects such as pectus excavatum, scoliosis, and finger contractures. Here, we report the case of a four-year-old girl referred to our clinic for multiple congenital anomalies, namely atrial and ventricular septal defects and pulmonary valve stenosis, bifid uvula, craniosynostosis, umbilical hernia, and finger contractures, resembling Loeys-Dietz syndrome, a hereditary connective tissue disorder (HCTD). At observation, it was noted borderline short stature and dysmorphic craniofacial features such as synophris, small nose, small deep-set eyes, micrognathia and microcephaly. Clinical exome sequencing revealed a pathogenic heterozygous variant in *ABL1* gene, c.1066G>A (p.(Ala356Thr)). The A356T variant was not observed in large population cohorts, and it has been previously reported as a *de novo* variant in an individual with features of congenital heart defects and skeletal malformations syndrome. The A356T variant leads to an amino acid substitution, which is likely to impact secondary protein structure. Functional studies indicate that the A356T variant is associated with increased tyrosine phosphorylation. The literature review showed that clinical features were similar to all other patients with *ABL* variants; our case is the first presenting with craniosynostosis. Parental genetic testing is ongoing to confirm the *de novo* origin of the mutation. This report further broadens the phenotypic spectrum of germline *ABL1* variants associated syndrome, adding craniosynostosis as a further disease-associated feature. However, we cannot completely exclude the possibility of this feature not being related to the main diagnosis. We suggest clinical and genetic reassessment of other cases with overlapping characteristics of HCTD since most next generation sequencing panels for HCTD often do not include the *ABL1* gene.

**P78-The mildest known phenotype in a patient with *GFM1* biallelic variant: case report and literature review**

Carolina Ribeiro^1^, Sérgio B. Sousa^1,2^, Maria do Carmo Macário^3^, Luísa Diogo^4^, João Mendes^1^, Maria José Simões^5^, Hugo Froufe^5^, Conceição Egas^5,6^, Sofia Pinto^7^, Catarina Correia^7^, Henriqueta C. Silva^1,8^, Jorge M. Saraiva^2,9^, Margarida Venâncio^1,2^

1- Medical Genetics Institute – UC Genomics, Faculty of Medicine, University of Coimbra, Coimbra, Portugal; 2- Medical Genetics Unit, Hospital Pediátrico, Centro Hospitalar e Universitário de Coimbra, Coimbra, Portugal; 3- Neurology Department, Centro Hospitalar e Universitário de Coimbra, Portugal; 4- Centro de Referência de Doenças Hereditárias do Metabolismo. Centro de Desenvolvimento da Criança - Hospital Pediátrico, Centro Hospitalar e Universitário de Coimbra, Coimbra, Portugal; 5- Genoinseq, Next-Generation Sequencing Unit, Biocant, Cantanhede, Portugal; 6- Center for Neuroscience and Cell Biology, University of Coimbra, Coimbra, Portugal; 7- Coimbra Genomics, Cantanhede, Portugal; 8- Center for Research in the Environment, Genetics and Oncobiology (CIMAGO), Faculty of Medicine, University of Coimbra, Coimbra, Portugal; 9- University Clinic of Pediatrics, Faculty of Medicine, Universidade de Coimbra, Coimbra, Portugal.

Introduction: In the last years, the evolution of next-generation sequencing, such as whole-genome or whole-exome sequencing (WES), revealed a growing number of metabolic disorders underlying intellectual disability (ID) syndromes, namely with milder or atypical phenotypes otherwise difficult to identify. Combined oxidative phosphorylation deficiency-1 due to biallelic pathogenic variants in the gene encoding the mitochondrial elongation factor G1 (*GFM1*) is an ultra-rare early onset progressive hepatoencephalopathy, associated with recurrent vomiting and metabolic decompensation episodes, persistent severe lactic acidemia and multiple respiratory chain deficiency, often fatal in infancy or childhood. We report on an adult patient, likely the oldest patient alive with this diagnosis. Clinical report: As part of an ID cohort study, In2Genome project, WES was performed on a 21-year-old female patient. This patient was born from a healthy couple with remote consanguinity. Gestation and delivery were uneventful. At birth, hypotonia was noticed that evolved to global psychomotor developmental delay reported since the second semester of life. The first time that the patient was referred to the Metabolic Unit, at 3y10m, she presented ataxic walking with inability to run, dysmetria, limited speech, failure to thrive and postnatal short stature. Later, she further developed seizures (onset at 5y) with abnormal EEG, pigmentary retinopathy (without significant vision defect), severe ID and osteoporosis. She had persistent increased serum lactate but further extend metabolic tests were normal, namely cerebral spinal fluid lactate, liver tests and respiratory chain studies in muscle biopsy. WES identified a known likely pathogenic homozygous missense variant in *GFM1* gene: c.2011C>T; p (Arg671Cys). Discussion: This specific variant is likely hypomorphic and has been reported previously in 3 cases, one also in homozygous state and two in compound heterozygosity. One of these patients, despite being a child at the time of report, also had a stable condition without decompensation episodes and with expected long-term survival.

**Financial support:** In2Genome project is funded by Centro Portugal Regional Operational Programme (CENTRO-01-0247-FEDER-017800), under the Portugal 2020 Partnership Agreement and the European Regional Development Fund.

**P79-Cardiospondylocarpofacial syndrome as a distinct hereditary connective tissue disorder: novel missense variant in MAP3K7 in two unrelated patients**

Joana Rosmaninho Salgado^1^, Janet Pereira^2^, Meriel McEntagart^3^, Sahar Mansour^3^, Piers Daubney^4^, Rachel Power^4^, Christine Hall^5^, Belinda Campos-Xavier^1^, Conceição Egas^6,7^, Hugo Froufe^6^, Maria José Simões^6^, Catarina Gomes^8^, Jorge M. Saraiva^1,9^, Sérgio B. Sousa^1,10^

1- Medical Genetics Unit, Hospital Pediátrico, Centro Hospitalar e Universitário de Coimbra, Coimbra, Portugal; 2_ Department of Hematology, Centro Hospitalar e Universitário de Coimbra; 3- St Georges University Hospitals NHS FT, UK; 4- Royal Brompton and Harefield Hospitals, UK; 5- Emerita, Department of Radiology, Great Ormond Street Hospital, WC1N 3JH London, UK; 6- Genoinseq, Next-Generation Sequencing Unit, Biocant, Cantanhede, Portugal; 7- Center for Neuroscience and Cell Biology, University of Coimbra, Coimbra, Portugal; 8- Coimbra Genomics, Cantanhede, Portugal; 9- University Clinic of Pediatrics, Faculty of Medicine, Universidade de Coimbra; 10- Medical Genetics Institute – UC Genomics, Faculty of Medicine, University of Coimbra, Coimbra, Portugal.

Introduction: Cardiospondylocarpofacial syndrome (CSCFS, ORPHA: 3238) firstly delineated by Sousa et al. (2010) is an autosomal dominant multisystemic condition to date reported in few cases, even after the causative has been identified (Le Goff et al., 2016): MAP3K7 that encodes TGF-β-activated kinase 1, an important regulator of p38 mitogen-activated protein kinase signaling pathway. We describe two patients from unrelated families (7th and 8th reported) with the same novel missense variant and expand the phenotypic spectrum.

Clinical report: A 12-year-old Portuguese male presented at birth with hypotonia, bilateral inguinal hernia, cryptorchidism, facial dysmorphisms (myopathic-like facies, puffy eyes, ptosis, hypertelorism, downslanting palpebral fissures) and hands with loose and wrinkled skin resembling cutis laxa. Subsequently, he had motor and speech delay and at 4 years a bilateral conductive hearing loss was diagnosed. The cardiac follow-up revealed a myxomatous mitral and tricuspid valves. At last examination, he had normal intellect and growth, pectus excavatum, flat foot, significant joint laxity, and maintained the skin, hands and facial features. Retrospective X-rays analysis revealed cervical vertebral fusions. Trio WES identified a de novo heterozygous missense variant in MAP3K7 gene, c.629G>A (p.Cys210Ser), absent in control databases and only reported in one case from DDD project at Decipher database. This patient is an 8-year-old British patient with short stature, dysmorphic features, mitral insufficiency, conductive hearing loss, carpal fusion and also connective tissue features: joint laxity, soft velvety skin, hands with redundant skin, flat foot and pectus excavatum.

Discussion: CSCF is likely underdiagnosed. WES unveiled this diagnosis in both our patients who presented significant connective tissue features, not highlighted in the initial patients described. Underlying molecular mechanisms will also be discussed, CSCF is caused by loss-of-function heterozygous MAP3K7 non-recurrent missense variants or in-frame deletions, while frontometaphyseal dysplasia type 2 is caused mainly by a recurrent gain-of-function MAP3K7 frameshift variant.

**Financial support:** First patient integrated in The In2Genome project: funded by Centro Portugal Regional Operational Programme (CENTRO-01-0247-FEDER-017800); Second patient integrated the DDD project at Decipher database

**P80-A new case of Snijders Blok-Campeau Syndrome**

João Rodrigues Alves^1^, Juliette Dupont^1^, Sílvia Jorge^2^, Isabel Cordeiro^1^, Ana Berta Sousa^1^

1- Serviço de Genética Médica, Hospital de Santa Maria, Centro Hospitalar e Universitário Lisboa Norte, Centro Académico de Medicina de Lisboa, Lisboa; 2- Unidade Funcional de Pediatria, Departamento da Criança, Hospital de Cascais, Cascais

Introduction: Multiple members of the chromodomain helicase DNA-binding (CHD) protein family have been implicated in the aetiology of a significant number of neurodevelopmental disorders. Recently, heterozygous pathogenic variants in *CHD3* have been found to cause a syndromic form of intellectual disability with a characteristic facial appearance, impaired speech and macrocephaly, known as Snijders Blok-Campeau Syndrome (SBCS). We describe a patient with a *CHD3* pathogenic variant, aiming to contribute to the phenotypic characterization of this recently described condition. Case report: We report a 30-month-old girl, first child of a healthy non consanguineous couple, with no relevant family history. Pregnancy and delivery were uneventful. Neonatal period was marked by floppiness, with preserved reflexes. Growth progressed on the 50th-75th centile. Evaluation at 30 months documented significant global developmental delay (all areas bellow 2SD, namely gross motor and language). Oromotor problems were remarkable. Physical examination revealed height and weight between the 50th and 75th centiles and head circumference in the 97th centile. Facial features included prominent forehead, deep-set eyes, periorbital fullness, narrow and downslanting palpebral fissures, and a prominent chin. Echocardiogram and cranial MRI were normal, as was arrayCGH. Whole exome sequencing identified an heterozygous pathogenic variant in *CHD3* gene (c.3130C>T p.(Arg1044Trp). Parental testing is ongoing. Discussion/Conclusions: The variant in *CDH3* gene identified in this patient is located on the surface of the Helicase C-terminal domain of CHD3 protein, and has been previously reported in five patients with SBCS. The facial features, initially suggestive of a Sotos-like syndrome, are very consistent with the cohort of 35 patients from the DDD study described by Snijders Blok et al. in 2018. This report of a new patient with SBCS raises awareness and contributes to the phenotypic characterization of this newly recognized condition, highlighting that it should be considered in the differential diagnosis of the overgrowth syndromes.

**P81-Novel *POGZ* gene truncating variant: a case report of syndromic intellectual disability**

Catarina S. Rosas^1^, Belinda Campos-Xavier^1^, Joaquim de Sá^1^, Maria José Simões^2^, Hugo Froufe^2^, Conceição Egas^2,3^, Sofia Pinto^4^, Catarina Correia^4^, Janet Pereira^5^, Lina Ramos^1,6^, Jorge M. Saraiva^1,7^, Ana Luísa Carvalho^1,8^

1- Medical Genetics Unit, Hospital Pediátrico, Centro Hospitalar e Universitário de Coimbra, Portugal; 2- Genoinseq, Next-Generation Sequencing Unit, Biocant, Cantanhede, Portugal; 3- Center for Neuroscience and Cell Biology, Universidade de Coimbra, Coimbra, Portugal; 4- Coimbra Genomics, Cantanhede, Portugal; 5- Department of Hematology, Centro Hospitalar e Universitário de Coimbra, Coimbra, Portugal; 6- Faculty of Health Sciences, Universidade da Beira Interior, Covilhã, Portugal; 7- University Clinic of Pediatrics, Faculty of Medicine, Universidade de Coimbra, Coimbra, Portugal; 8- University Clinic of Genetics, Faculty of Medicine, Universidade de Coimbra

Introduction: Numerous novel candidate genes associated with developmental disorders are being identified based on the application of large-scale cohort based whole exome sequencing. Among them, stands *POGZ* - pogo transposable element with zinc finger domain, implicated in the normal mitotic progression, as a regulator of chromatin remodeling and proper chromosome segregation. Initially associated with isolated neurodevelopmental disorders, like autism spectrum disorder and intellectual disability, it has more recently been associated to syndromic intellectual disability phenotypes. Case report: We report a case of a ten-year-old girl, first child to non-consanguineous and healthy parents, that later gave birth to two healthy sisters. She presented prenatally with augmented nuchal translucency and the diagnosis of Tetralogy of Fallot. Global developmental delay was evident in early infancy, with more prominent speech impairment, currently associated with attention deficit hyperactivity disorder and mild conductive hearing loss. Karyotype and array comparative genomic hybridization were normal. Distinct facial dysmorphic features became gradually apparent during her childhood, in addition to thoracic asymmetry and one cafe-au-lait spot on her right knee. Whole exome sequencing identified a novel heterozygous frameshift variant c.3843_3846del (p.Lys1281Asnfs∗28) in *POGZ* gene, subsequently validated by Sanger sequencing. Parental testing revealed it to be a de novo variant. Discussion: We present a novel truncating variant in *POGZ* gene associated with a syndromic phenotype, here including unusual and remarkable cardiac involvement, apart from various facial features similar to those depicted in literature, increasing the number of presently described similar cases. The presented clinical case supports the relevance of a wide molecular study approach and reinforces that truncating variants in *POGZ* may lead to an identifiable syndromic intellectual disability.

**Financial support:** The In2Genome project is funded by Centro Portugal Regional Operational Programme (CENTRO-01-0247-FEDER-017800) under Portugal 2020 Partnership Agreement through the European Regional Development Fund.

**P82-Co-occurrence of pathogenic mutations in patients at-risk for Hereditary Breast and Ovarian Cancer: two cases diagnosed using a multi-gene panel**

Natália Salgueiro^1^, Cláudia Martins^1^, Ariana Conceição^1^, Diana Pinto^1^, Nataliya Tkachenko^2^, Gabriela Soares^2^, Cláudia Falcão Reis^2^, Elsa Garcia^1^, Marta Moreira^1^, Lisandra Castro^1^, Ana Fortuna^2^, Margarida Reis-Lima^1^

1- Unidade de Biologia Molecular, Synlab-Genética Médica Porto;2- Serviço de Genética, Centro Hospitalar do Porto.

Introduction: Breast Cancer (BC) is the second most common cancer worldwide, and the most frequent cancer in women. Ovarian cancer (OC), is the fourth common cause of female cancer death in the developed world. About 10–30% of BC and OC show a familial aggregation but it is estimated that only 5–10% of BC and 20% of OC are hereditary. The main genes involved in hereditary breast and ovarian cancer (HBOC) are BRCA1 and BRCA2. Multiple recent studies have shown that a percentage of high-risk individuals have germline pathogenic mutations in cancer risk genes other than BRCA1 and BRCA2, such as ATM, BRIP1, CDH1, CHEK2, NBN, PALB2, RAD51C, RAD51D, PTEN, TP53, EPCAM, STK11.

In this study we report two cases with co-occurrence of pathogenic mutations in breast and ovarian high-risk cancer genes. Methods: We performed NGS analysis of 18 genes: BRCA1, BRCA2, ATM, BRIP1, CDH1, CHEK2, NBN, PALB2, RAD51C, RAD51D, PTEN, TP53, EPCAM, STK11, MLH1, MSH2, MSH6 e PMS2. Also, MLPA for BRCA1, BRCA2, EPCAM genes and BRCA2 c.156_157insAlu was performed. Results: We identified 2 probands with co-occurrence of pathogenic mutations. One proband had co-occurrence of pathogenic variants in BRCA1 and RAD51C genes. This proband had triple negative breast cancer at the age of 48 years old and no familial history. The other proband had co-occurrence of pathogenic variants in PALB2 and CHEK2. This proband had breast cancer at the age of 23-year-old and colorectal cancer at 55 and positive family history for breast cancer. Discussion: The use of a multigene panel is nowadays considered the best strategy to improve detection of pathogenic mutations in high-risk HBOC genes. Furthermore, this strategy enables the identification of rare situations as co-occurrence of actionable pathogenic mutations in patients. The identification of individuals with multiple clinically actionable mutations have important implications for probands and their family members. Although higher risk and aggravation of the phenotype in those patients should be expected and a specific surveillance plan implemented, further investigations is needed to better understand the implications of having multiple pathogenic mutations.

**P83-PIK3CA-related overgrowth spectrum: case report**

Sara M Ribeiro^1^, Mariana Quelhas^1^, Leonor Ramos^2^, Maria Alexandra Oliveira^3^, Pedro Figueiredo^4^, Pedro Cardoso^5^, Filipe Palavra^6^, Isabel Alonso^7,8^, Jorge M Saraiva^1,9^, Sérgio B Sousa^1,10^

1- Medical Genetics Unit, Hospital Pediátrico, Centro Hospitalar e Universitário de Coimbra, Coimbra, Portugal; 2- Dermatology Unit, Centro Hospitalar e Universitário de Coimbra, Coimbra, Portugal; 3- Paediatric Oncology Unit, Hospital Pediátrico, Centro Hospitalar e Universitário de Coimbra, Coimbra, Portugal; 4- Physical Medicine and Rehabilitation Unit, Hospital Pediátrico, Centro Hospitalar e Universitário de Coimbra, Coimbra, Portugal; 5- Orthopedics Unit, Hospital Pediátrico, Centro Hospitalar e Universitário de Coimbra, Coimbra, Portugal; 6- Child Developmental Center, Hospital Pediátrico, Centro Hospitalar e Universitário de Coimbra, Coimbra, Portugal; 7- UnIGENe and Centro de Genética Preditiva e Preventiva, Institute for Molecular and Cellular Biology, Porto, Portugal; 8- Instituto de Ciências Biomédicas Abel Salazar, Universidade do Porto, Porto, Portuga; 9- University Clinic of Pediatrics, Faculty of Medicine, University of Coimbra, Portugal; 10- University Clinic of Genetics, Faculty of Medicine, University of Coimbra, Portugal

Introduction: PIK3CA-related overgrowth spectrum (PROS) encompasses a range of complex progressive conditions associated with asymmetric overgrowth caused by mosaic postzygotic activating variants in PIK3CA gene, which encodes for the 110-kD catalytic alpha subunit of PIK3. These variants lead to increased downstream catalytic activity, promoting cell survival and proliferation through AKT and mTOR signaling, important pathways also in cancer development. Clinical Report: We report a three-year-old boy, the only child of healthy non-consanguineous parents. During pregnancy, pleural effusion with spontaneous resolution occurred. At birth, he had extensive capillary malformation in the trunk and limbs and striking large hands and feet. In the first six months of life, all growth parameters climbed in the growth charts; at 30-months-old, he had weight at 77th, height at 66th and head circumference at 97th centile. Segmental overgrowth has been affecting mainly limbs soft tissues and he further developed extensive epidermal nevi. Heart and abdominal ultrasounds were normal. Brain MRI showed focal left temporal cortical dysplasia type II, mild ventriculomegaly and a slight descent of the cerebellar tonsils without Chiari malformation. Psychomotor development has been normal. WES-based virtual panel of 12 genes from the PI3K/AKT/mTOR pathway identified the heterozygous pathogenic variant c.2176G>A p.(Glu726Lys) in PIK3CA gene at the mosaic state in DNA directly extracted from affected skin. Discussion: We report a new patient with a known PIK3CA variant, previously described in patients with Megalencephaly-Capillary Malformation (MCAP) syndrome and curiously also as somatic mutation in different types of cancer. This patient's phenotype integrates well within PROS, to date fitting better the MCAP description but evolving with components of Congenital Lipomatous Overgrowth, Vascular Malformations, Epidermal Nevi, Scoliosis/Skeletal and Spinal (CLOVES) syndrome. Due to the progression of this group of conditions and often poor long-term prognosis, the efficacy of recently described targeted therapies such as PIK3CA inhibitors constitute a real hope for these patients.

**P84-A new case with a splicing *PIEZO2* mutation causing distal arthrogryposis with distal muscle weakness, scoliosis and proprioception defects**

Pedro M Almeida^1,2^, Maria José Simões^3^, Joaquim Sá^1,5^, Janet Pereira^4^, Carolina Ribeiro^5^, Hugo Froufe^3^, Conceição Egas^3,6^, Sofia Pinto^7^, Catarina Correia^7^, Jorge M Saraiva^1,8^, Fabiana Ramos^1^

1- Medical Genetics Unit, Hospital Pediátrico, Centro Hospitalar e Universitário de Coimbra, Coimbra, Portugal; 2- Faculty of Health Sciences, University of Beira Interior, Covilhã; 3- Genoinseq, Next-Generation Sequencing Unit, Biocant, Cantanhede, Portugal; 4- Department of Hematology, Centro Hospitalar e Universitário de Coimbra; 5- Medical Genetics Institute – UC Genomics, Faculty of Medicine, University of Coimbra, Coimbra, Portugal; 6- Center for Neuroscience and Cell Biology, University of Coimbra, Coimbra, Portugal; 7- Coimbra Genomics, Cantanhede, Portugal; 8- University Clinic of Pediatrics, Faculty of Medicine, Universidade de Coimbra, Coimbra, Portugal

Introduction: Homozygous or compound heterozygous loss-of-function mutations in the piezo-type mechanosensitive ion channel component 2 (*PIEZO2*) gene were identified in patients with sensory ataxia and proprioception defects together with arthrogryposis, myopathy, scoliosis and progressive respiratory failure. The PIEZO2 protein functions as a nonselective cation channel, is expressed in sensory endings of proprioceptors innervating muscle spindles, Golgi tendon organs and Merkel cells. To date only 17 patients were described with this phenotype. Clinical Report: We report a 23-year-old girl, first child of healthy and non consanguineous parents, that was born with hypotonia, distal laxity, club feet, contractures/arthrogryposis, and feeding difficulties. She was referred to our unit due to a previous diagnosis of a congenital hypomyelinating neuropathy without molecular confirmation, intellectual disability, short stature, mild restrictive pulmonary disfunction, sleep disturbance, dysmorphic features, an asymmetric involvement of shoulder and trunk muscles, progressive severe kyphoscoliosis and mild mitral insufficiency. She is able to walk eyes open, with a wide-based, unsteady gait and without support. She performed an extensive etiological investigation that was inconclusive, and, after this, we included this patient in I2G Project for whole exome sequencing (WES) that identified a previously reported pathogenic splice site homozygous variant (c.5083-1G>A) in *PIEZO2* gene. Discussion: The observed symptoms of sensory ataxia and proprioception defect with muscle weakness, distal arthrogryposis and progressive scoliosis represent core characteristics of a recently delineated condition caused by biallelic loss-of-function *PIEZO2* mutations. As in this case, all patients have been identified by WES without previous clinical recognition, highlighting the importance of this approach in this group of disorders and suggesting that this condition is likely underdiagnosed.

**Financial support:** In2Genome project is funded by Centro Portugal Regional Operational Programme (CENTRO-01-0247-FEDER-017800), under the Portugal 2020 Partnership Agreement and the European Regional Development Fund.

**P85-A Prenatal Case of a fetus with Inverted Duplication and Terminal Deletion 5p**

Rita Monteiro^1^, André Sampaio^2^, Mafalda Lopes^1^, Paula Lindo^1^, Joana Trindade^1^, Maria João Oliveira^1^, Fernanda Baltar^1^, Alina Queirós^1^, Cláudia Alves^1^, Cecília Correia^1^, Margarida Reis-Lima^1^

1- Unidade de Citogenética, SYNLABHEALTH Genética Médica, Porto; 2- Unidade de DPN, Serviço de Obstetrícia, Hospital Divino Espírito Santo, Ponta Delgada

Introduction: Inverted duplication and deletion of 5p is a complex chromosomal rearrangement (CCR) rarely described in the literature. Phenotypic features of the carriers are variable, depending on the size of the duplicated and deleted segments. Clinical diagnosis of trisomy 5p is difficult, due to the broad spectrum of manifestations. Total duplications of 5p arm or a small segment between 5p10–5p13.3 usually determine a more severe phenotype, the latter being proposed as the critical region for trisomy 5p. Trisomy of segments distal to 5p13 mostly causes mild features. A 35-year-old pregnant woman was referred for prenatal diagnosis due to fetal cystic hygroma and increased nuchal translucency. Amniocentesis was performed at 14 weeks of gestation. Methods: Routine aneuploidy screening was performed by Multiplex-PCR, and amniotic fluid sample cultured and harvest followed in situ protocol. Parental karyotypes were analysed by conventional cytogenetics. FISH study for 5p15.2 region and array-CGH ISCA 8x60K (Oxford Gene Technology, UK) were performed. Results: Aneuploidy screening revealed absence of aneuploidies in a female fetus. Fetal karyotype showed additional material at the terminal region of chromosome 5p, suggestive of a duplication. Two co-localized signals in the short arm of the abnormal chr. 5 were observed by FISH, indicating duplication of the cri-du-chat critical region (5p15.2) and that the abnormality was an inverted duplication of 5p arm. Parental karyotypes were normal. The fetal karyotype was described as 46,XX,add(5).ish dup(5)(p15.2p15.2)(D5S23++,D5S721++)dn. Array CGH study showed a deletion of 1.64 Mb in 5p15.33 region and a contiguous duplication of 42.8 Mb at 5p15.33p12. Discussion: We present a prenatal case of a *de novo* inverted duplication and terminal deletion of chromosome 5p initially detected by conventional cytogenetic study, confirmed by FISH and fully characterized by array CGH analysis. Fetal ultrasound examination at 18 weeks showed increased nuchal translucency, large edema of the neck, hyperchogenic bowel and short long bones. Pregnancy was terminated at 19 weeks of gestation. Fetal autopsy is in progress. This case demonstrates the value of the combination of several cytogenetic techniques, mainly the usefulness of array CGH analysis for the characterization of CCR, such as inv dup del, which are rarely observed in prenatal context.

**P86-Novel mutation in addition to functional *TMPRSS6* gene polymorphisms originate an IRIDA-like phenotype in an African child**

Bárbara D. Faleiro^1^; Raquel Maia^2^; Sara Batalha^2^; Joana Mendonça^1^; Miguel P. Machado^1^; Luís Vieira^1,3^; João Lavinha^1,4^; Paula Faustino^1,5^

1- Departamento de Genética Humana, Instituto Nacional de Saúde Doutor Ricardo Jorge, Lisboa; 2- Unidade de Hematologia, Hospital de Dona Estefânia, Centro Hospitalar Universitário de Lisboa Central (CHULC), Lisboa; 3- ToxOmics, Faculdade de Ciências Médicas, Universidade Nova de Lisboa, Lisboa; 4- BioISI, Faculdade de Ciências, Universidade de Lisboa, Lisboa; 5- Instituto de Saúde Ambiental (ISAMB), Faculdade de Medicina, Universidade de Lisboa, Lisboa; Portugal

Iron-refractory iron deficiency anemia (IRIDA) is a rare autosomal recessive anemia often unresponsive to oral iron intake and partially responsive to parenteral iron treatment. The disease originates from mutations in *TMPRSS6* gene, encoding Matriptase 2, a transmembrane serine protease that plays an essential role in down-regulating hepcidin. Once *TMPRSS6* is mutated, the corresponding protein is absent or inactive at the hepatocyte membrane leading to uncontrolled high levels of hepcidin and impaired iron absorption. This study aimed to investigate a 4-year-old boy of sub-Saharan ancestry (Mozambique/Angola), presenting with microcytic hypochromic anemia, low transferrin saturation, normal ferritin, and having a partial response to intravenous iron treatment. He is a -α3.7-thalassemia carrier. *TMPRSS6* was screened for variants by Next-Generation Sequencing using Nextera XT libraries in a *MiSeq* platform (*Illumina*). Genetic variants found were validated by Sanger sequencing. *In silico* analyses were performed in HSF, SIFT, Poly-Phen2 and Missense3D softwares. A novel missense mutation (c.871G>A) was found in heterozygosity, in *TMPRSS6* exon 8. *In silico* analysis indicates the conserved amino acid change (G291S) may be damaging to the protein stability. Due to its location in the CUB1 domain, it may also affect the enzyme activation and substrate recognition. Additionally, 3 SNPs previously associated with a greater risk of developing iron deficiency anemia (K253E, V736A and Y739Y) were also identified in *TMPRSS6*. Although IRIDA is known as an autosomal recessive disease, we conclude that, in this case, the result of a digenic inheritance of the novel damaging mutation (c.871G>A; G291S) and the 3 common modulating SNPs in the same gene and a co-inheritance of the α-thalassemia *HBA* deletion may lead to an IRIDA-like phenotype. Further functional studies of the mutated protein as well as family studies should be conducted.

**P87-A complex intrachromosomal 4q rearrangement: a case report**

Marta Souto^1,2^, Pedro Botelho^1,2^, Regina Arantes^1,2^, Susana Sousa^2,3^, Márcia Martins^2,4^, Osvaldo Moutinho^2,5^, Rosário Pinto Leite^1,2^

1- Laboratório de Genética, Centro Hospitalar Trás-os-Montes e Alto Douro, Vila Real; 2- Departamento da Mulher e da Criança, Centro Hospitalar Trás-os-Montes e Alto Douro, Vila Real; 3- Serviço de Pediatria, Centro Hospitalar Trás-os-Montes e Alto Douro, Vila Real; 4- Consulta de Genética, Centro Hospitalar de Trás-os-Montes e Alto Douro, Vila Real; 5- Serviço de Ginecologia/Obstetrícia, Centro Hospitalar Trás-os-Montes e Alto Douro, Vila Real

Introduction: Deletions or duplications on the long arm of chromosome 4 are a rare event. Clinical manifestations depend on the amount of the genetic material detected or duplicated and vary from healthy to several degrees of intellectual disability and multiple congenital anomalies. 4q deletion has been associated with craniofacial dysmorphism, micrognathia and heart defects. Growth deficiency, microcephaly, facial dysmorphism, hypotonia, difficulties feeding has been related with 4q duplication. The authors present a case of a newborn with a complex intrachromosomal rearrangement on the long arm of chromosome 4

Clinical report: New born with respiratory distress syndrome, hypotonia, peculiar facies and feeding difficulties has attended a genetic consultation. Cytogenetic analysis revealed a *de novo* translocation between the long arm of chromosome 4 and the short arm of chromosome 20. Array Comparative Genomic Hybridization (aCGH) detected a deletion of 9 Mbp on 4q24 region and a gain of four copies of 1Mbp on 4q23 region and three copies of 1.7 Mbp on 4q22.3 region.

Discussion: In the present case, conventional cytogenetics detected a translocation between chromosomes 4 and 20. The array clarify a complex rearrangement in 4q24 region involved in translocation that comprise a loss of 9 Mbp and a total gain of 9.1 Mbp. The newborn has several dysmorphias consistent with 4q deletion and duplication syndrome. An isolated 4q duplication or 4q deletion are very rare structural anomalies with a heterogeneous spectrum of clinical manifestations. To our knowledge this is the second case of a duplication deletion involving chromosome 4q.

